# A basement membrane discovery pipeline uncovers network complexity, regulators, and human disease associations

**DOI:** 10.1126/sciadv.abn2265

**Published:** 2022-05-18

**Authors:** Ranjay Jayadev, Mychel R. P. T. Morais, Jamie M. Ellingford, Sandhya Srinivasan, Richard W. Naylor, Craig Lawless, Anna S. Li, Jack F. Ingham, Eric Hastie, Qiuyi Chi, Maryline Fresquet, Nikki-Maria Koudis, Huw B. Thomas, Raymond T. O’Keefe, Emily Williams, Antony Adamson, Helen M. Stuart, Siddharth Banka, Damian Smedley, Genomics England Research Consortium, David R. Sherwood, Rachel Lennon

**Affiliations:** 1 Department of Biology, Duke University, Box 90338, Durham, NC 27708, USA.; 2 Wellcome Centre for Cell-Matrix Research, Division of Cell-Matrix Biology and Regenerative Medicine, School of Biological Sciences, Faculty of Biology Medicine and Health, University of Manchester, Manchester Academic Health Science Centre, Manchester M13 9PT, UK.; 3 Manchester Centre for Genomic Medicine, Manchester University Hospitals NHS Foundation Trust, Manchester M13 9WL, UK.; 4 Division of Evolution and Genomic Sciences, School of Biological Sciences, Faculty of Biology, Medicine and Health, University of Manchester, Manchester Academic Health Science Centre, Manchester M13 9PT, UK.; 5 Genome Editing Unit Core Facility, Faculty of Biology, Medicine and Health, University of Manchester, Manchester M13 9PT, UK.; 6 William Harvey Research Institute, Charterhouse Square, Barts and The London School of Medicine and Dentistry, Queen Mary University of London, EC1M 6BQ London, UK.; 7 Genomics England, London, UK.; 8 Department of Paediatric Nephrology, Royal Manchester Children’s Hospital, Manchester University Hospitals NHS Foundation Trust, Manchester Academic Health Science Centre, Manchester M13 9WL, UK.

## Abstract

Basement membranes (BMs) are ubiquitous extracellular matrices whose composition remains elusive, limiting our understanding of BM regulation and function. By developing a bioinformatic and in vivo discovery pipeline, we define a network of 222 human proteins and their animal orthologs localized to BMs. Network analysis and screening in *C. elegans* and zebrafish uncovered BM regulators, including ADAMTS, ROBO, and TGFβ. More than 100 BM network genes associate with human phenotypes, and by screening 63,039 genomes from families with rare disorders, we found loss-of-function variants in *LAMA5*, *MPZL2*, and *MATN2* and show that they regulate BM composition and function. This cross-disciplinary study establishes the immense complexity of BMs and their impact on in human health.

## Teaser

Basement membrane, the most ancient animal extracellular matrix, has vast component complexity and human disease associations.

## INTRODUCTION

Basement membranes (BMs) are the most ancient animal extracellular matrix (ECM) and form sheet-like structures that underlie epithelia and surround most tissues (*1*). Two independent planar networks of laminin and type IV collagen molecules associate with cell surface interactors (CSIs) and provide the scaffolding structure that builds BMs along tissues (*2*). The glycoprotein nidogen and the heparan sulfate proteoglycan perlecan bridge the laminin and collagen IV scaffolds (*2*). BMs also harbor matricellular proteins, growth factors, and proteases (*1*). BMs have diverse compositions tailored to resist mechanical stress, dictate tissue shape, and create diffusion barriers (*2*–*5*). They also provide cues that direct cell polarity, differentiation, migration, and survival (*4*, *6*, *7*). Underscoring their diverse and essential functions, variants in more than 20 BM genes underlie human diseases (*8*). BM proteins are targets of autoantibodies in immune disorders (*9*) and defects in BM protein expression and turnover are a key pathogenic aspect of cancer, diabetes, and fibrosis (*10*–*12*).

The number of proteins that associate with BMs remains unclear. Gene Ontology (GO) and ECM annotations estimate between 24 and 100 BM proteins (*10*, *13*), while proteomic studies suggest that BMs contain more than 100 proteins (*13*). Challenges with protein solubility, abundance, and loss of spatial characteristics, however, have limited the use of biochemical methods in identifying BM constituents (*10*). In silico prediction has identified more than 1000 putative matrisome (ECM and ECM-associated) proteins in vertebrates and more than 700 in *Caenorhabditis elegans* (*14*–*16*). As there are many different ECMs, it is unknown whether many of these components bind to function within or regulate BMs. Validation of a candidate BM protein requires its microscopic localization to a BM in vivo (*13*), and many matrix proteins have been detected within BMs (*17*–*20*). A few large-scale efforts have attempted to establish the number of BM proteins. For example, the Matrixome project, a mouse study combining in silico computational screening, in vitro functional screening, and in vivo immunohistochemical screening identified seven previously unknown BM proteins and characterized the localization of 36 mouse BM proteins in total (*20*). Furthermore, work in *C. elegans* has characterized more than 30 fluorophore-tagged endogenous BM proteins (*3*, *21*). However, without a focused effort to curate the many studies identifying BM proteins in humans, as well as across animal model systems, and the development of strategies to identify BM proteins, our understanding of BMs in normal and disease states remains limited.

In this study, we develop a bioinformatic and in vivo localization pipeline and define a comprehensive network of 222 human genes and their animal orthologs encoding BM matrix proteins and BM CSIs (collectively referred to as BM zone genes), many of which show tissue-specific expression and BM localization in humans and mice. Using domain network analysis, we identify key regulatory hub proteins, including perlecan and papilin, which maintain BM structure and promote BM turnover. Through knockdown studies in *C. elegans* and zebrafish, we reveal that Robo receptors and transforming growth factor–β (TGFβ) signaling limit BM type IV collagen levels. We further identify human gene-phenotype associations for 112 BM network genes. By screening the rare disease cohort in the Genomics England 100,000 Genomes Project (100KGP) (*22*), we uncover disease associations for rare loss-of-function (LoF) variants in *MATN2*, *MPZL2*, and *LAMA5* and demonstrate that these variants are linked to defective BM composition, structure, and function. Collectively, this cross-disciplinary approach provides insights into BM assembly, regulation, and disease and establishes the vast complexity of BMs and their critical importance in human health.

## RESULTS

### A comprehensive BM gene network across species

**Fig. 1.**
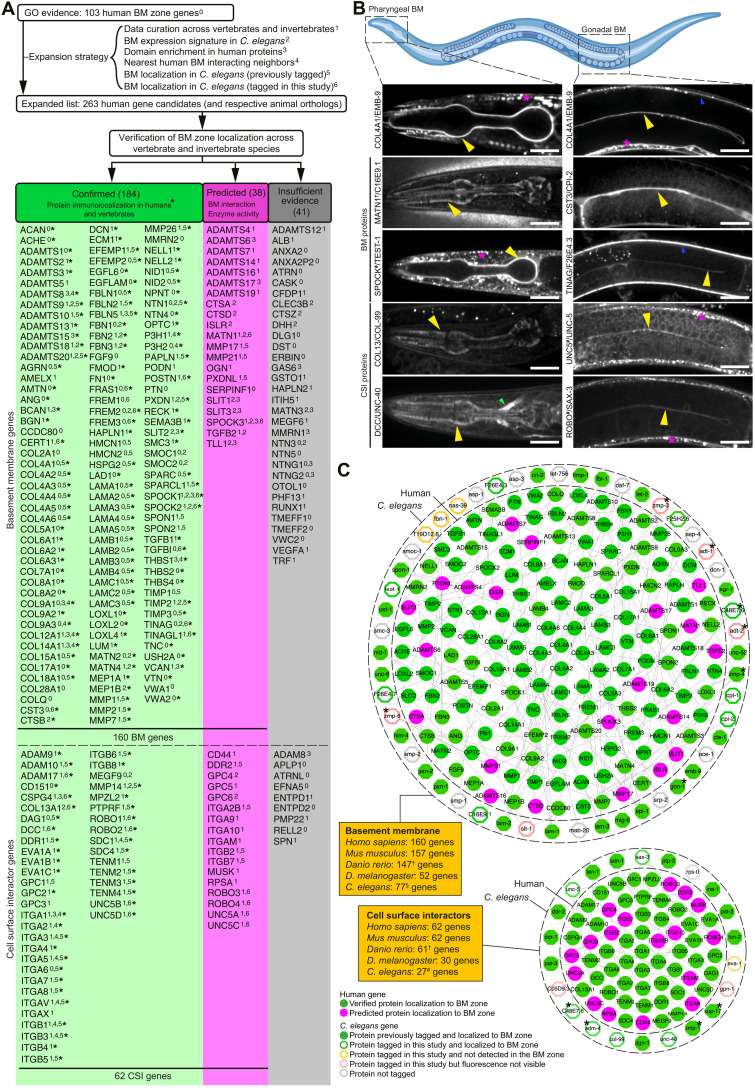
A comprehensive and conserved network of BM zone genes. (**A**) Strategy for identifying putative BM matrix protein and CSI genes in humans and other species. Protein localization to BM zone was either (i) confirmed through vertebrate tissue immunolocalization (green column) or (ii) predicted on the basis of BM protein interaction or BM-cleaving protease activity (magenta column). Fluorescent tagging studies in *C. elegans* provided further localization support for components in both columns. Genes with insufficient evidence are in the gray column. *160 components are localized to the human BM zone. (**B**) Top: Schematic representation of the pharyngeal and gonadal BMs in *C. elegans* (Biorender). Middle: Confocal mid-plane *z*-slices of adult animals expressing endogenously tagged COL4A1/EMB-9::mRuby2. Bottom: Selected BM zone candidates tagged with mNG in this study. BM zone localization in respective tissues is indicated by yellow (pharynx and gonad), blue (body wall muscle), and green (nerve ring) arrowheads. Asterisks denote punctate intracellular signal within body wall muscle tissue, a major site of BM protein synthesis. †MATN1 is a predicted human candidate and its ortholog localized to the BM zone in *C. elegans*. #Localization evidence present for two of four UNC5, one of three SPOCK, and one of four ROBO family members in humans (table S7). Scale bars, 25 μm. (**C**) Integrated BM zone network of 222 human genes (circles, 160 BM matrix and 62 CSI components) with corresponding *C. elegans* orthologs (octagons). Lines link ortholog pairs. Boxes indicate number of ortholog genes in mouse, zebrafish, *Drosophila*, and *C. elegans*. *Complex ortholog families in *C. elegans* (detailed in fig. S4). †Duplicated zebrafish genes were counted as one; worm §astacins and #tetraspanins are not included. Network is curated at https://bmbase.manchester.ac.uk/.

The number of BM-associated proteins has not been rigorously determined. To establish a comprehensive network of BM zone genes, we first identified 103 human “basement membrane”–annotated genes from the GO Resource (*23*). We then curated data across multiple animal species for known BM components and predicted candidates through gene expression, protein interaction, and domain enrichment analyses (see Materials and Methods; Supplementary Text; tables S1 to S5; and fig. S1, A and B), which yielded 160 additional BM zone candidates (table S6). Next, we confirmed BM zone localization for 184 of 263 candidates based on protein immunolocalization studies in vertebrates. Among these 184 confirmed BM components, 36 *C. elegans* orthologs that were endogenously tagged with genetically encoded fluorophores were previously localized to BMs (Fig. 1A, fig. S1A, and tables S5 to S7). Localization for 38 of the remaining candidates was predicted on the basis of protein interaction with verified BM zone proteins and BM-cleaving protease activity (Fig. 1A and fig. S1A). To provide further evidence for possible BM zone association for these candidates and proteins with limited BM immunolocalization evidence, 24 *C. elegans* orthologs were endogenously tagged with mNeonGreen (mNG) (fig. S2). We examined all postembryonic BMs and highlight the pharynx (feeding organ) and gonad BMs [Fig. 1B, EMB-9::mRuby2 (COL4A1)]. For proteins with limited BM immunolocalization evidence, TEST-1 [testican (SPOCK)], CPI-1 [cystatin-C (CST3)], CPI-2 (CST3), UNC-5 (netrin receptor UNC5), and SAX-3 (ROBO) localized to both BMs, while ADM-4 [disintegrin and metalloproteinase domain-containing protein 17 (ADAM17)], C48E7.6 [chondroitin sulfate proteoglycan 4 (CSPG4)], and F26E4.7 [periostin (POSTN)/TGFBI] localized to the pharyngeal BM, thus providing further support for these proteins as BM constituents (Fig. 1B, fig. S3A, and table S5). Among proteins with only predicted BM localization, the membrane-associated C16E9.1 (MATN1) polarized to the pharyngeal BM but was not detected in the gonad (Fig. 1B and table S5). NAS-39 [tolloid-like protein 1 (TLL1)], EVA-1 (EVA1), and FBN-1 [fibrillin (FBN)] were not detected in BMs but were visible in other ECMs (fig. S3B and table S5). Together, these fluorescent tagging studies localized 14 additional proteins to the BM zone. Forty-one candidates without verified or predicted BM localization were not considered for further analyses (Fig. 1A and tables S6 to S7). In sum, this pipeline defined an expanded network of 160 human BM matrix proteins and 62 CSIs with orthologs in rodents, *C. elegans*, zebrafish, and *Drosophila* that localize to the BM zone (Fig. 1C, figs. S1B and S4, and table S7). To curate the BM zone network, we established the basement membraneBASE database (https://bmbase.manchester.ac.uk).

### Identification of tissue-specific BM composition signatures

**Fig. 2.**
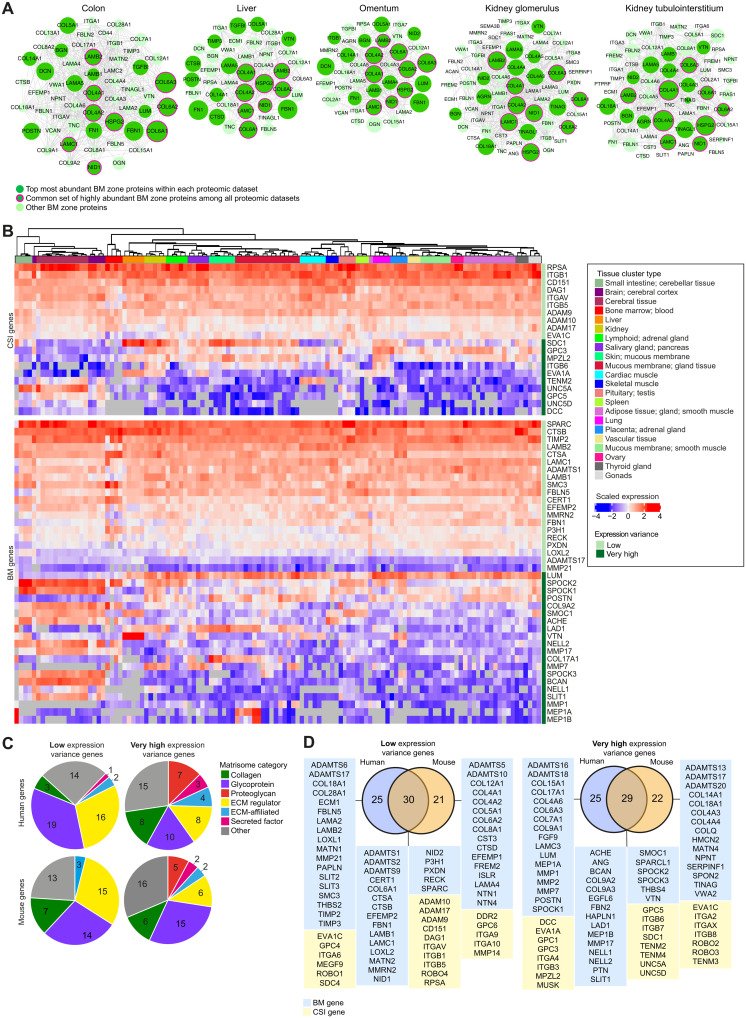
Mammalian BMs of different tissues have unique compositional signatures. (**A**) Interactomes (based on published proteomic datasets) representing the diversity of BM composition across human tissues. Proteins are depicted as nodes sized according to their log-transformed relative tissue abundance. Lines represent protein interactions determined by STRING analysis (table S4). (**B**) Heatmaps derived from published transcriptomic datasets (table S9) depict either very high or low expression variance of BM zone genes across multiple human tissues. (**C**) Classification of human and mouse BM and CSI genes with differing expression variance according to matrisome category. (**D**) Overlap between human BM zone genes and mouse orthologs for very high or low expression variance. See fig. S5 and table S10 for extended expression variance data.

To investigate BM diversity in human tissues, the relative abundance of the BM zone network within proteomic datasets for human kidney, liver, colon, and omentum was ranked (table S8). We found a common set of BM zone proteins that were abundant across all tissues, including COL4A1/2, COL6A1/A2/A3, LAMB2/C1 (laminin subunit beta-2/gamma-1), NID1 (nidogen-1), and HSPG2 [BM-specific heparan sulfate proteoglycan core protein (perlecan)], and identified proteins with highly variable abundance, such as DCN (decorin) and TINAG (tubulointerstitial nephritis antigen) (Fig. 2A). Moreover, distinct patterns of protein composition and abundance within tissues were apparent (see kidney glomerulus compared to tubulointerstitium; Fig. 2A). Analysis of 121 human and 51 mouse tissue transcriptomes was conducted to determine whether these differences were linked to gene expression (tables S9 to S10). Dynamic clustering revealed strong BM gene grouping by tissue (Fig. 2B and fig. S5, A and B). Furthermore, there was segregation by tissue compartment (e.g., cerebral cortex and cerebellum) and a distinct clustering pattern in organs with mucosal linings (e.g., intestine, fallopian tubes, gallbladder, and urinary bladder), suggesting that BM expression signatures associate with tissue functions (Fig. 2B and fig. S5A). Ranking BM genes by expression variance revealed that low-variance genes (expressed in most tissues) were predominantly glycoproteins and matrix regulators such as laminins, nidogens, and papilin (Fig. 2, C and D, and table S10). Very high-variance genes included SPARC-related genes, many collagen chains, and secreted BM-binding factors (Fig. 2, C and D). Together, these analyses identify protein composition and gene expression patterns that contribute to BM signatures for different human tissues and reveal both common BM template components and tissue-specific BM factors.

### Domain network analysis reveals BM hub proteins

**Fig. 3.**
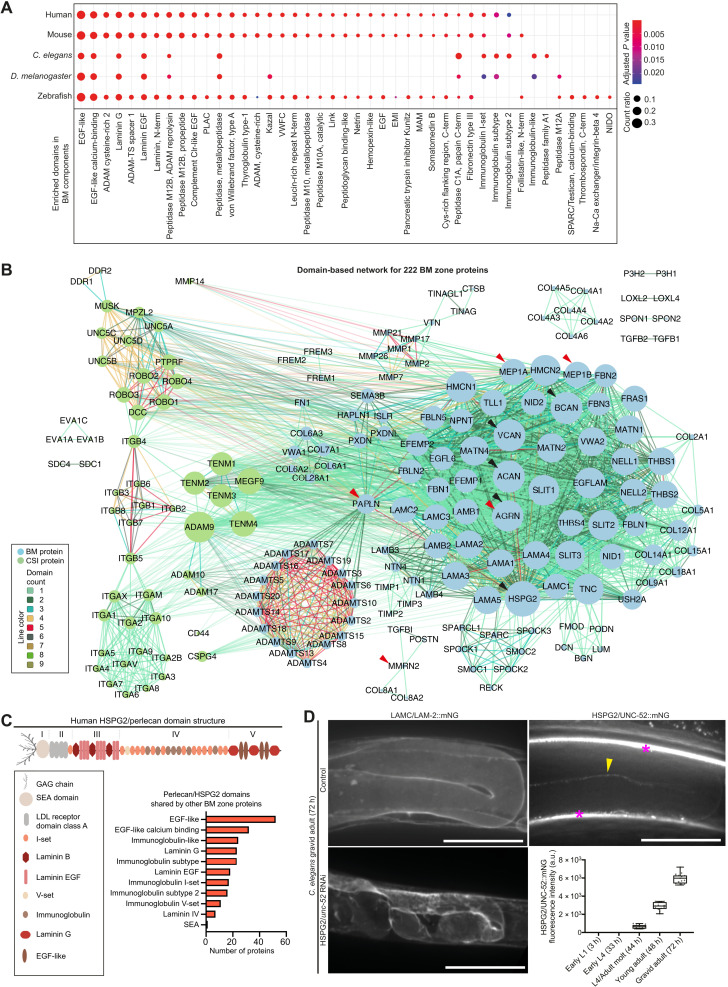
Domain network analysis reveals hub proteins in the BM zone. (**A**) Dot plot depicts conserved and enriched InterPro protein domains in BM zone components. Dots are sized according to number of domain occurrences within BM genes (count ratio; table S3). (**B**) Domain-based interactome for human BM zone genes. Nodes represent BM and CSI proteins and are sized according to network degree score. Lines connecting nodes are color-coded to indicate the number of shared domains. Black and red arrowheads highlight hub proteins with the highest degree and betweenness centrality scores, respectively (table S11). (**C**) Domain structure of the hub protein HSPG2/perlecan. Bar chart shows the number of BM zone proteins sharing specific domains with HSPG2. (**D**) Left: Representative confocal sum projections of *C. elegans* gonadal BM LAMC/LAM-2::mNG in control versus HSPG2*/unc-52* RNA interference (RNAi)–treated 72-hour adult animals (*n* = 10 animals examined each; additional supporting images are available on figshare: 10.6084/m9.figshare.c.5662348). Top right: Middle-plane confocal *z*-slice of HSPG2/UNC-52::mNG in an adult animal. Yellow arrowhead indicates fluorescence signal in the gonadal BM, and asterisks denote strong signal in the body wall muscle BM and muscle-epidermal attachment sites. Bottom right: Quantification of gonadal BM UNC-52::mNG levels throughout postembryonic development (*n* ≥ 13 for each developmental stage; fluorescence was not detected between early L1 and early L4 stages). Scale bar, 25 μm. For boxplots, edges indicate the 25th and 75th percentiles, the line in the box represents the median, and whiskers mark the minimum and maximum values. a.u., arbitrary units.

The identification of more than 200 BM zone proteins greatly expands the number of components that constitute BMs. Elucidating the function and interaction of BM proteins is crucial to understanding the specific roles and regulation of BMs in tissue development and homeostasis. Biochemical analysis of protein-protein interactions in native BMs is challenging because of the limited solubility, extensive posttranslational modifications, and the cross-linked nature of BM components (*15*). Furthermore, many proteins in our comprehensive BM network have limited or no biochemical characterization. Reflecting these shortcomings, less than 20% of interactions reported for BM zone proteins within the STRING protein-protein interaction database (*24*) were classified as high-confidence interactions (fig. S6A). Construction of a BM zone protein interaction network using these data revealed that many proteins were excluded because of a lack or scarcity of reported interaction data (fig. S6B). To bypass these limitations, we turned to protein domain analysis, as protein domains provide insight into both the function of a protein and its potential interaction with other proteins. We reasoned that an analysis of protein domains represented within BM zone proteins would help identify groups of BM proteins with similar functions, as well as BM proteins with many and diverse domains that might bridge interactions between BM proteins and regulate BM organization. We first compared our BM zone network against entire proteomes for human, mouse, zebrafish, *Drosophila*, and *C. elegans* and identified 35 protein domains enriched within BM components across these species (Fig. 3A and fig. S7A). The epidermal growth factor (EGF)–like and EGF-like calcium-binding domains were the most frequent domains in all species, likely because of their involvement in diverse functions, including signaling and adhesion and as rigid structural spacers (*25*). Next, using this list of BM-enriched domains, we generated networks for human and *C. elegans* BM zone proteins, where connections between BM zone components denote the presence of shared domains (Fig. 3B and fig. S7B). We found distinct protein subnetworks based on domain commonality and shared functions, such as integrins, ADAMTS proteases, and collagen IV chains, and a broader module including laminins, other glycoproteins, and proteoglycans (Fig. 3B). The same clustering pattern was present in *C. elegans*, indicating shared modular assembly of BM proteins (fig. S7B).

To quantify the extent of shared domains within BM network proteins, we calculated measures of network connectivity (Materials and Methods). We first determined the degree score, which indicates the number of connections between proteins based on shared domains. Among BM matrix proteins, the heparan sulfate proteoglycan perlecan (HSPG2) had the highest degree score (table S11). HSPG2 contains 10 different protein domains and shares at least one domain with 84 other BM proteins (Fig. 3C). The *C. elegans* perlecan ortholog UNC-52 also had the highest degree score among the worm BM matrix proteins (table S11). These observations suggest that perlecan could have complex functions and act as a regulatory hub within BMs. The precise role of perlecan in BMs is not clear, as its loss causes embryonic lethality in mice and *C. elegans* (*26*, *27*). In *Drosophila*, postembryonic perlecan depletion results in misshapen organs, suggesting a role within BMs in shaping tissues (*28*, *29*). We examined postembryonic perlecan (UNC-52) in *C. elegans* and found its localization in the adult gonadal BM (Fig. 3D). Depletion of UNC-52 caused a progressive compaction of the gonad from young adulthood, correlating with the onset of perlecan localization (*n* = 10 of 10 animals examined at each developmental stage; Fig. 3D and fig. S7C). Furthermore, the smooth appearance of the sheet-like BM was disrupted by fibrillar structures and aggregates of laminin, collagen IV, nidogen, and papilin (*n* = 5 of 5 examined for each BM component; Fig. 3D and fig. S7D). These data indicate a shared role for perlecan in shaping tissues and reveal a function in stabilizing BM organization.

We next calculated the betweenness centrality score, which favors BM proteins that have a hybrid domain structure: domain and domain arrangements that are found in distinct groups of BM zone proteins. For example, the proteoglycan papilin (PAPLN) and its *C. elegans* ortholog MIG-6 share domain structure with diverse classes of BM and CSI proteins, including ADAMTS proteases, peroxidasins, various proteoglycans, the netrin receptor DCC/UNC-40, and ROBO (Fig. 3B and fig. S7, B and E), and PAPLN and MIG-6 had the highest betweenness centrality scores (table S11). We reasoned that BM zone proteins such as PAPLN/MIG-6 that harbor hybrid domain structures might bridge interactions between different classes of proteins. Supporting this notion, a recent study found that *C. elegans* MIG-6 promotes collagen IV turnover by restricting the BM localization of ADAMTS proteases and peroxidasin (*21*). Additional BM matrix and CSI proteins with high network connectivity scores were identified, including agrin (AGRN), meprin A subunit alpha (MEP1A), aggrecan core protein (ACAN), teneurins, DCC, and ROBO, suggesting that they could have roles in BM regulation (Fig. 3B and table S11). Collectively, these domain network analyses pinpoint BM constituents that are candidate organizers of multiple interactions within BMs, regulating key BM properties such as growth, shape, and integrity.

### Functional in vivo studies uncover regulators of BM composition

**Fig. 4.**
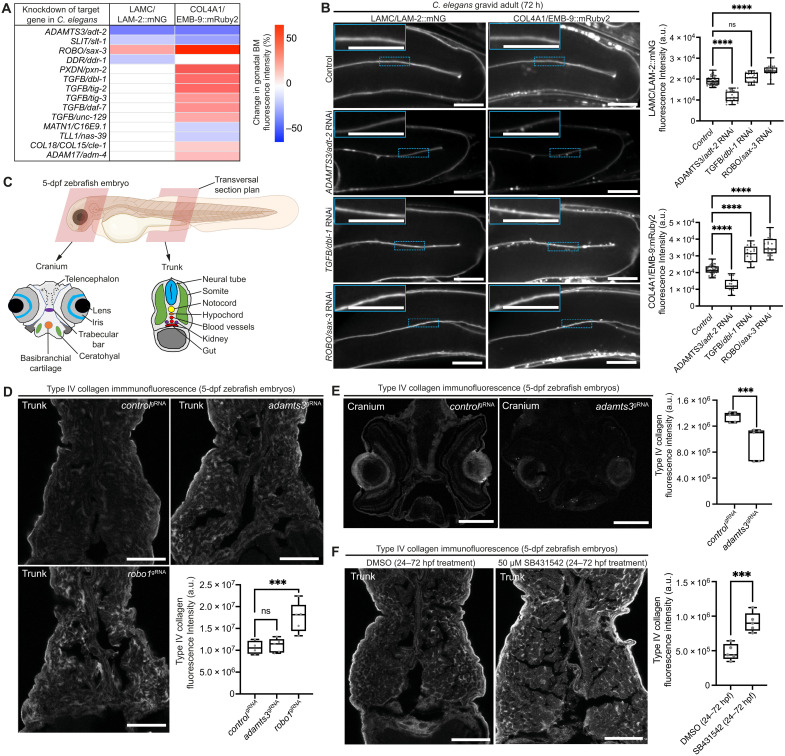
Regulators of BM composition in *C. elegans* and zebrafish. (**A**) Heatmap summarizing changes in LAMC/LAM-2::mNG and COL4A1/EMB-9::mRuby2 fluorescence in the gonadal BM upon target gene knockdown in *C. elegans*. (**B**) Confocal middle-plane *z*-slices of gonadal BM LAMC/LAM-2::mNG and COL4A1/EMB-9::mRuby2 in control and RNAi-targeted *adt-2*, *dbl-1*, and *sax-3* 72-hour adult animals (boxed regions are magnified in insets) with quantifications of fluorescence intensity shown on the right (*n* ≥ 20 each). ****P < 0.0001; ns, not significant; one-way analysis of variance (ANOVA) with post hoc Dunnett’s test. Scale bars, 25 μm. (**C**) Schematic of a zebrafish embryo [5 days postfertilization (dpf); Biorender] with transversal cross sections depicting tissues in the cranium and the trunk. (**D**) Confocal images of type IV collagen immunofluorescence in trunk sections of *control*^gRNA^-, *adamts3*^gRNA^-, and *robo1*^gRNA^-injected 5-dpf embryos. Fluorescence intensity within the entire section is quantified on the bottom right (*n* = 5 for each treatment). ***P < 0.001, one-way ANOVA with post hoc Dunnett’s test. Scale bars, 30 μm. (**E**) Collagen IV immunofluorescence in cranial sections of *control*^gRNA^- and *adamts3*^gRNA^-injected 5-dpf embryos with quantification of fluorescence intensity on the right (*n* = 5 for each treatment). ***P < 0.001, unpaired Student’s *t* test. Scale bars, 30 μm. (**F**) Collagen IV immunofluorescence in trunk sections of 5-dpf embryos treated with dimethyl sulfoxide (DMSO; control) or SB431542 (TGFBR1 inhibitor) on the left [2-day treatment between 24 and 72 hours postfertilization (hpf)]; quantification of fluorescence intensity on the right (*n* = 5 each). ***P < 0.001, unpaired Student’s *t* test. Scale bars, 30 μm. For boxplots, edges indicate the 25th and 75th percentiles, the line in the box represents the median, and whiskers mark the minimum and maximum values.

Little is known about how BM composition is controlled during tissue growth. We thus performed an RNA interference (RNAi) screen targeting 77 *C. elegans* BM zone orthologs and examined laminin and collagen IV in the gonadal BM, which expands ~100-fold in surface area during larval development (table S12). Knockdown of 19 genes affected BM composition, including previously reported integrin receptor and papilin genes (Fig. 4, A and B, and table S12) (*3*, *21*). Of the newly identified regulatory genes, knockdown of *adt-2* (*ADAMTS3*) caused a 40% reduction in BM laminin and collagen IV levels and led to misshapen gonads (Fig. 4B and table S12). Depletion of SAX-3 (ROBO), which strongly polarizes to the gonadal BM (Fig. 1B), increased laminin and collagen IV levels (Fig. 4B). Loss of the ROBO ligand SLT-1/SLIT, however, did not show a similar phenotype, suggesting an SLT-independent function for ROBO (Fig. 4A and table S12). Last, loss of the TGFβ ligand–encoding genes *dbl-1*, *tig-2*, *tig-3*, *daf-7*, and *unc-129* and the two TGFβ type I receptor–encoding genes *daf-1* and *sma-6* led to elevated collagen IV levels without affecting laminin (Fig. 4B; fig. S8, A and B; and table S12). Consistent with a cell-autonomous role for TGFβ signaling in regulating collagen IV levels, the sole *C. elegans* TGFβ type II receptor DAF-4 polarizes toward the gonadal BM (fig. S8C).

We next investigated whether ADAMTS3, ROBO, and TGFβ have roles in regulating vertebrate BM composition. A*damts3* and *robo1* CRISPR-knockdown (crispant) (*30*) zebrafish exhibited intracerebral hemorrhaging, which also occurs after *col4a1* knockdown due to vascular BM disruption, and defective fin folds, which is associated with BM dysfunction (fig. S8, D to G) (*31*). In addition, we examined kidney physiology, which depends on BMs for filtration function (*32*). Using the *NL:D3* excretion assay (*33*), proteinuria was observed in *adamts3* and *robo1* crispants, suggesting defects in BM (fig. S8H). To visualize BM type IV collagen in many tissues, immunofluorescence analysis was performed in trunk sections of zebrafish larvae (Fig. 4C). These studies revealed elevated collagen IV levels in *robo1* crispant zebrafish, similar to ROBO loss in *C. elegans* (Fig. 4D). As collagen IV levels were unchanged in *adamts3* crispant zebrafish trunks, we further examined cranial sections (Fig. 4C). Collagen IV levels were reduced in the head regions of *adamts3* crispant zebrafish, consistent with cerebral hemorrhaging in these animals (Fig. 4E). These findings suggest a tissue-specific role for ADAMTS3 in the regulation of BM collagen IV levels. Last, to investigate TGFβ signaling, zebrafish larvae were treated with the TGFβ type I receptor inhibitor SB431542, which resulted in increased BM collagen IV levels, also mirroring *C. elegans* (Fig. 4F). Together, these in vivo screens support the emerging role of ADAMTS proteins in BM regulation (*21*, *34*) and uncover a function for the Robo receptor and TGFβ signaling in limiting BM collagen IV levels.

### Expansion of human BM disease associations

**Fig. 5.**
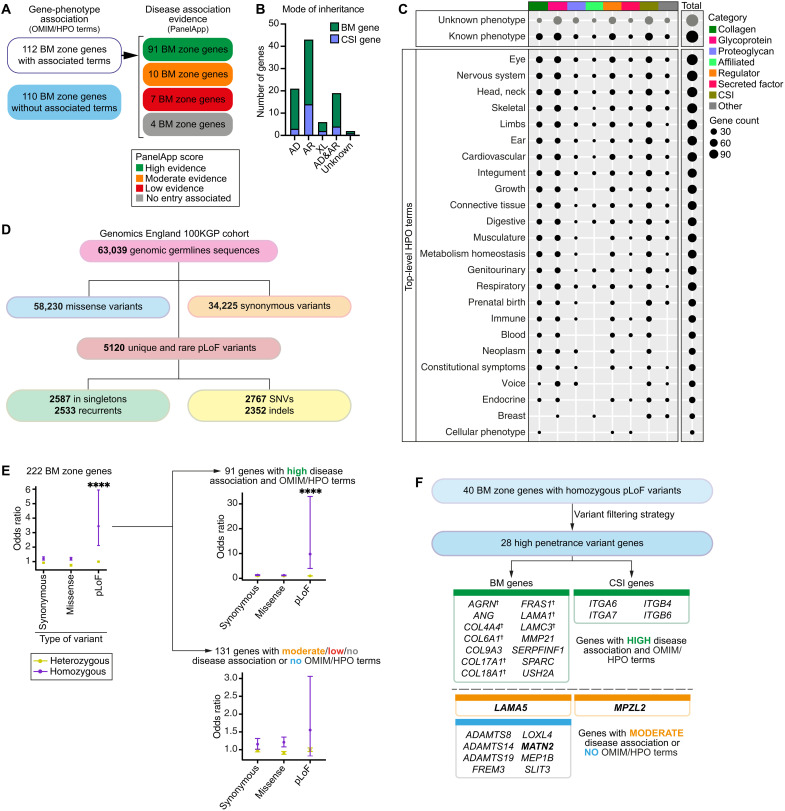
Expansion of human disease associations and identification of candidate disease-causing variants in BM zone genes. (**A**) Classification of BM zone genes according to phenotypes within OMIM and HPO databases (left) and level of evidence for disease causation within the Genomics England PanelApp database (right) (see also tables S13 and S14). (**B**) Bar chart depicting mode of inheritance for 112 BM zone genes with disease associations (AD, autosomal dominant; AR, autosomal recessive; XL, X-linked). (**C**) Dot plot illustrates phenotypic abnormalities (HPO terms) associated with BM zone genes grouped by their matrisome classifications. Dot size indicates gene count in each category. (**D**) Landscape of BM zone gene variants identified in the Genomics England 100KGP cohort (table S16). SNVs, single-nucleotide variants. (**E**) Odds ratio (OR) plots (comparing individuals with disease to unaffected relatives) for all variants (left) or variants grouped according to disease association evidence for the respective BM zone genes (right). ****P < 0.0001, Fisher’s exact test. (**F**) High-penetrance homozygous predicted LoF (pLoF) variants were identified for 28 BM zone genes, including **†**components previously associated with BM-linked disease, and disease candidates. Potential disease-causing mechanisms were investigated for genes highlighted in bold.

Previous studies established that germline variants in ~30 BM genes cause genetic disorders, approximately half of which encode collagen IV and laminin chains (*8*). We used our BM zone network to search for disease associations in the Human Phenotype Ontology (HPO) (*35*), Genomics England PanelApp (*36*), and Online Mendelian Inheritance in Man (OMIM) (*37*) databases. Gene-phenotype associations with varying degrees of evidence were found for 112 BM zone genes, and most were associated with autosomal recessive inheritance (Fig. 5, A and B, and tables S13 to 14). Collagen- and glycoprotein-encoding genes such as *COL2A1*, *COL4A1*, *COL7A1*, *FBN1*, and *TGFBI* and the proteoglycan *HSPG2* showed the highest number of associations with phenotypic abnormalities (top-level HPO terms; Fig. 5C and table S14). The eye, nervous system, head and neck, skeletal system (bones and joints), and limbs had disease phenotype associations with more than 100 BM zone genes, suggesting sensitivity of these organ systems to BM component loss (Fig. 5C and table S14). Together, this analysis greatly expands BM gene association with human disease and reflects BMs’ diverse roles in supporting tissues.

### Genomic studies identify candidate disease variants in BM zone genes

The Genome Aggregation Database (gnomAD) is the largest collection of publicly available data on population-level variation in human genes (*38*). To further explore human disease associations with BM zone genes, we examined gnomAD metrics [probability of LoF intolerance (pLI) and LoF observed/expected upper bound fraction (LOEUF) scores; Materials and Methods] for tolerance to predicted LoF (pLoF) variation for the 222 BM zone gene loci. Fifty-one genes had low tolerance (LOEUF score, <0.2), suggesting that loss of one functional copy of these genes results in inviability or drives rare dominantly inherited disorders (table S15). Next, to determine whether BM zone genes could contribute to recessively inherited disorders, we used whole-genome sequencing data in the 100KGP. We examined germline genomic sequences for 34,842 individuals affected with rare disease and 28,197 unaffected relatives and identified 34,225 synonymous, 58,230 missense, and 5120 pLoF heterozygous or homozygous rare (minor allele frequency, <0.01) variants in BM zone genes (Fig. 5D and table S16). Odds ratio (OR) analysis revealed that homozygous pLoF variants in 40 BM zone genes were significantly enriched in affected individuals [OR = 3.4, 95% confidence interval (CI) = 2.1 to 5.9, *P* < 0.001; Fig. 5E, table S17, and fig. S9A]. Notably, homozygous pLoF variants in collagens and glycoproteins showed increased ORs (fig. S9B), mirroring the disease phenotype analysis (Fig. 5C). We next stratified the BM zone genes by known disease association evidence and found the OR for homozygous pLoF variants in high evidence genes was greatly elevated for affected 100KGP individuals (OR = 9.8, 95% CI = 4.0 to 33.0, *P* < 0.001; Fig. 5E). We also observed a trending OR increase for homozygous pLoF variants in genes with moderate to no disease associations (OR = 1.5, 95% CI = 0.8 to 3.1; Fig. 5E), suggesting that these could be drivers of human disease associated with BM pathology.

### Disease-associated variants in *MATN2* perturb secretion and translation

**Fig. 6.**
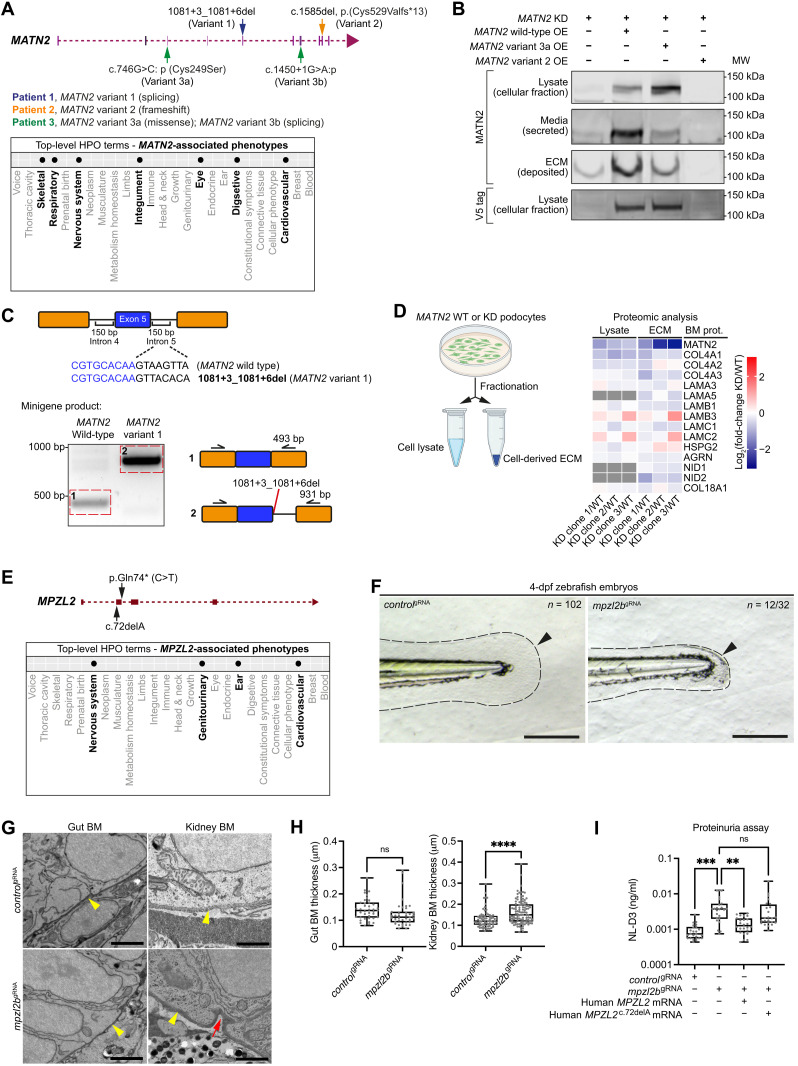
LoF *MATN2* variants are associated with human disease, and MPZL2 loss disrupts zebrafish BMs. (**A**) *MATN2* genomic structure indicating four pLoF variants (top) and associated phenotypic abnormalities represented as HPO terms (bottom, bolded). (**B**) Western blots of MATN2 in lysate and ECM fractions derived from endogenous MATN2-depleted human podocytes overexpressing V5-tagged wild-type or variant *MATN2*. Note that the faint band in the ECM fraction for the *MATN2*-KD lane is likely residual endogenous MATN2 due to variability in *MATN2* knockdown. Immunoblotting source data are available on figshare: 10.6084/m9.figshare.c.5662348. (**C**) In vitro minigene splicing assay demonstrating altered splicing of *MATN2* variant 1. bp, base pairs. (**D**) Fold change in BM component levels upon *MATN2* knockdown as determined by fractional proteomic analysis of podocyte-derived ECM (see table S18; additional quantification data are available on figshare: 10.6084/m9.figshare.c.5662348). (**E**) *MPZL2* genomic structure indicating two 100KGP pLoF variants (top) and associated phenotypic abnormalities (bottom, bolded). (**F**) Bright-field images of tail regions (dashed lines) in *control*^gRNA^- and *mpzl2b*^gRNA^-injected 4-dpf zebrafish embryos. Arrowheads highlight reduced fin fold extension in *mpzl2b* crispants. Scale bars, 100 μm. (**G**) Transmission electron microscopy (TEM) of gut and kidney BMs (yellow arrowheads) in control and *mpzl2b* crispant embryos. Red arrows indicate BM irregularities. Scale bars, 2 μm (gut) and 1 μm (kidney). (**H**) Quantification of gut (*n* = 39 each) and kidney (*n* = 100 each) BM thickness. ****P < 0.0001, unpaired Student’s *t* test. (**I**) Assessment of proteinuria (NL-D3 levels) in *mpzl2b* crispants injected with wild-type human *MPZL2* mRNA or *MPZL2*^c.72del^ variant mRNA (*n* = 24 each). ***P < 0.001 and **P < 0.01, one-way ANOVA with post hoc Dunnett’s test. For boxplots, edges indicate the 25th and 75th percentiles, the line in the box represents the median, and whiskers mark the minimum and maximum values.

Among the 40 homozygous pLoF variant genes, 28 fitted a high-penetrance variant filtering strategy (table S17 and Materials and Methods). Notably, 10 of these genes had limited or no known disease associations, and we focused on 3 of these that appeared to be possible candidates underlying BM disease (Fig. 5F). First, we examined variants in *MATN2*, as three families were identified carrying unique biallelic *MATN2* variants with a range of multisystem phenotypes (Fig. 6A, fig. S10A, and Supplementary Text). The matrilin MATN2 is a poorly understood matrix protein that has not been linked to human genetic disease. To determine whether these variants disrupt MATN2 translation and secretion, we used human podocytes that secrete endogenous MATN2 and assemble a BM-like ECM (*39*). CRISPR-Cas9 knockdown of *MATN2* nearly abolished the ECM fraction of MATN2, which was rescued by overexpression of wild-type V5-tagged *MATN2* (Fig. 6B and fig. S10B). In contrast, overexpression of the *MATN2*^c.746G>C, p.Cys249Ser^*-V5* missense variant resulted in MATN2 accumulation in the cellular fraction, suggesting a defect in secretion (Fig. 6B). Structural modeling showed disruption of a disulfide bond in the EGF1 domain of MATN2^c.746G>C, p.Cys249Ser^, consistent with a misfolded protein (fig. S10C). In a similar rescue experiment, the MATN2^c.1585del, p.Cys529Valfs*13^-V5 frameshift variant protein was not detected in the cellular or ECM fractions, implying defective translation (Fig. 6B and fig. S10D). In addition, a minigene splicing assay (*40*) confirmed aberrant splicing and the introduction of a premature stop codon for the predicted splicing variant *MATN2*^c.1081+3-1081+6del^ (Fig. 6C and fig. S10E). These analyses suggest that the *MATN2* variants perturb proper protein production, thus validating our variant filtering strategies. As *C. elegans* and zebrafish lack MATN2 orthologs, we next analyzed podocyte-derived matrix by proteomics to determine whether loss of *MATN2* affects BM composition. Levels of core BM components nidogen and collagen IV were decreased; laminin chains were variably affected, and perlecan was increased (Fig. 6D and table S18). Supporting these findings, immunofluorescence analysis of the podocyte matrix also revealed reduced deposition of nidogen and type IV collagen upon knockdown of *MATN2* (fig. S10F). Together, these studies establish that LoF *MATN2* variants could be associated with human disease and that loss of *MATN2* likely alters BM composition.

### BM dysfunction linked to *MPZL2* and *LAMA5* variants

Next, we examined two genes with high-penetrance variants but limited BM-associated disease phenotypes: *MPZL2* and *LAMA5* (Fig. 5F). MPZL2 (myelin protein zero-like protein 2) is a transmembrane glycoprotein (*41*) with genomic variants associated with hearing loss (*42*). We identified one 100KGP individual carrying a homozygous pLoF variant *MPZL2*^c.72del^ with kidney cysts, cerebral aneurysms, and hypertension: phenotypes connected to BM defects (Fig. 6E, fig. S11A, and Supplementary Text). To investigate whether *MPZL2* loss affects BM structure or function, we CRISPR-depleted *mpzl2b* in zebrafish and observed abnormal fin development and thickened kidney BMs (Fig. 6, F to H). Consistent with impaired BM function, we also observed elevated proteinuria (Fig. 6I). Notably, coinjection of human *MPZL2* mRNA rescued kidney function, while the *MPZL2*^c.72del^ variant mRNA failed to rescue filtration defects (Fig. 6I). Together, these results indicate that MPZL2 regulates BM structure and function and suggest the *MPZL2*^c.72del^ variant could be a driver of BM-associated disease.

**Fig. 7.**
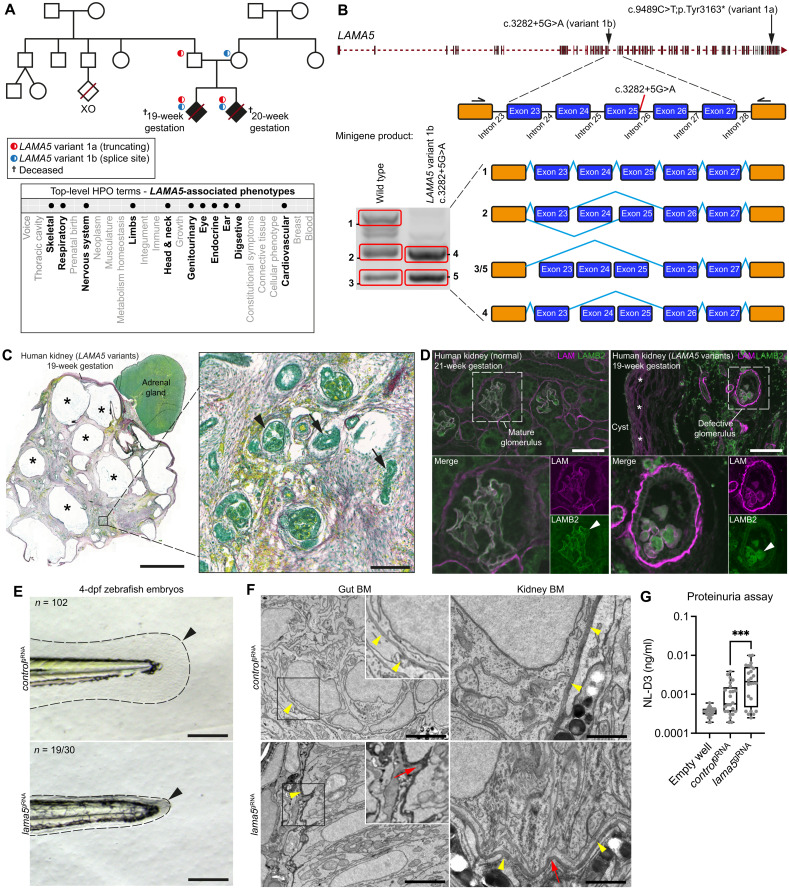
Genomic variants in *LAMA5* affect BM structure and function. (**A**) Top: Genetic pedigree for a 100KGP family carrying two *LAMA5* pLoF variants. Bottom: Phenotypic abnormalities observed in two *LAMA5* variant–carrying fetuses (19- and 20-week gestation) represented as bolded HPO terms. (**B**) Top: *LAMA5* genomic structure and variant locations. Bottom: In vitro minigene splicing assay demonstrating altered splicing of *LAMA5* variant 1b. (**C**) Left: Picrosirius red/Fast Green staining of the 19-week dysplastic kidney; asterisks indicate cysts. Boxed area is magnified on the right; arrowhead and arrows denote abnormal glomerulus and tubules, respectively. Scale bars, 5 mm (left) and 100 μm (right). (**D**) Pan-laminin (LAM; magenta) and laminin β2 (LAMB2; green) immunofluorescence in normal (left) and dysplastic (right) fetal kidney sections. Asterisks indicate laminin surrounding a cyst. Boxed regions are magnified below. Arrowheads, see text. Scale bars, 50 μm. Full uncropped images are available on figshare: 10.6084/m9.figshare.c.5662348. (**E**) Bright-field images of zebrafish tail regions (dashed lines) in *control*^gRNA^- and *lama5*^gRNA^-injected 4-dpf embryos. Arrowheads highlight reduced fin fold extension in *lama5* crispants. Scale bars, 100 μm. (**F**) TEM of gut and kidney BMs (yellow arrowheads) in control and *lama5* crispants. Boxed regions are magnified in insets. Red arrows indicate BM morphological irregularities. Scale bars, 2 μm (gut) and 1 μm (kidney). (**G**) Proteinuria in control versus *lama5* crispants (*n* = 24 each). ***P < 0.001, unpaired Student’s *t* test. For boxplots, edges indicate the 25th and 75th percentiles, the line in the box represents the median, and whiskers mark the minimum and maximum values.

LAMA5 is a laminin alpha chain that, together with LAMB2 and LAMC1, forms the laminin-521 network in BMs (*43*). Its loss results in skin and kidney defects in mice (*44*), and in humans, biallelic missense variants are associated with phenotypes across multiple systems (*45*, *46*). We identified a family with two early fetal losses where both fetuses carried two pLoF *LAMA5* variants: a truncating variant *LAMA5*^c.9489C>T;p.Tyr3163*^ in trans to a predicted splice variant *LAMA5*^c.3282+5G>A^ (Fig. 7A and Supplementary Text). Minigene analysis confirmed altered splicing for *LAMA5*^c.3282+5G>A^ (Fig. 7B). Both fetuses had abnormalities in multiple systems, including absent or dysplastic kidneys (Fig. 7A, fig. S11B, and Supplementary Text). We acquired the 19-week fetal kidney tissue and observed multiple cysts and defective glomerular architecture (Fig. 7C). Visualization of the laminin-521 network with LAMB2 immunofluorescence revealed diffuse intracellular LAMB2 signal within abnormal glomeruli in the dysplastic kidney, which contrasted to crisp glomerular BM localization in a normal fetus (Fig. 7D). Furthermore, pan-laminin (LAM) immunofluorescence was markedly thickened around abnormal structures in the dysplastic kidney, indicating that loss of *LAMA5* disrupts regulation of other BM laminin molecules (Fig. 7D). To further investigate the impact of *LAMA5* loss, we depleted *lama5* in zebrafish and observed abnormal fin fold development, ultrastructural irregularities and indentations in the gut and kidney BMs, and increased proteinuria (Fig. 7, E to G). Together, our findings demonstrate that *LAMA5* is crucial for vertebrate kidney BM structure and function and establish that biallelic LoF variants in *LAMA5* cause an early-onset human developmental disorder, thus providing a likely diagnosis for the family.

## DISCUSSION

**Fig. 8.**
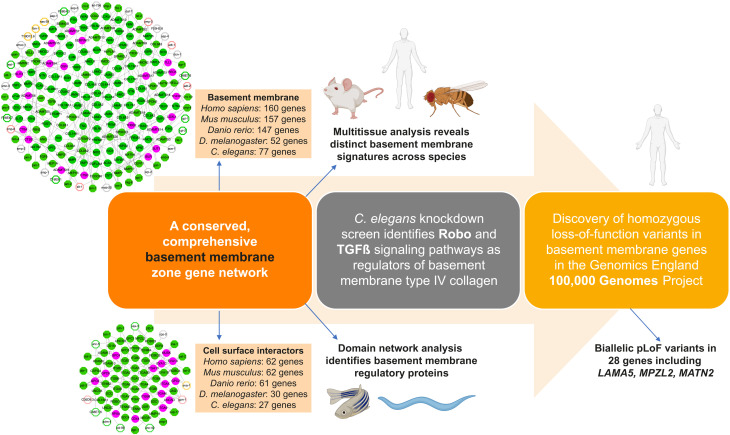
A BM discovery pipeline. Summary of multidisciplinary approaches and model systems used in this study that established a comprehensive network of BM matrix proteins and their CSIs, uncovered regulators of BM structure and function, and identified BM component genomic variants associated with phenotypic abnormalities in 100KGP.

We developed a pipeline combining bioinformatic approaches and in vivo localization to comprehensively annotate BM composition (Fig. 8). Our analyses define the makeup of the BM zone: 160 human BM matrix proteins and 62 CSIs with orthologs in rodents, *C. elegans*, zebrafish, and *Drosophila*. We show that 57 human BM proteins and 45 CSIs are shared with mice, zebrafish, *Drosophila*, and *C. elegans*. More than 50 are found solely in vertebrates and consist largely of proteoglycans, collagens, collagen-modifying enzymes, and expanded ADAMTS protease family members. Using human transcriptomic and proteomic datasets, we identified tissue-level signatures of BM gene expression and composition, which could have clinical utility as organ health readouts and disease biomarkers. The clustering of BM gene expression according to tissues suggests that BM component synthesis may be transcriptionally regulated to accommodate the distinct requirements of tissues. Notably, growth factors and signaling molecules such as fibroblast growth factor 9, EGFL6, and SLIT1 were among the BM components with the highest gene expression variance, likely reflecting their signaling functions in specific tissues. Although most known central BM components such nidogen and laminin have low expression variance between tissues, type IV collagen had highly variable expression. As type IV collagen levels can dictate tissue-specific mechanical and signaling properties, it is likely that expression levels of collagen IV genes are variable to meet precise tissue needs (*47*). Analysis of proteomic datasets revealed that there were only five abundant and common BM zone proteins across analyzed tissues: laminin, collagen IV, collagen VI, nidogen, and perlecan. This suggests that a limited core BM scaffolding supports vast tissue-specific combinations of other BM residents, which likely provide tissues’ unique mechanical and signaling support and help account for the many functions of BMs. A caveat is that some core BM scaffolding proteins may not be detected in proteomic datasets because of challenges associated with ECM protein solubility, especially for proteoglycans (*48*). For example, papilin, which has sulfated glycosaminoglycan chains (*49*), was detected in only two of the five examined proteomic datasets (kidney compartments) at low abundance, although it is expressed in most human tissues that we examined. However, recent localization studies of its ortholog MIG-6 in *C. elegans* showed that it is a ubiquitous and highly abundant BM component, suggesting that papilin is likely a core BM protein (*21*).

Biochemical experiments and studies of embryonic BM assembly have provided insights into how the core BM scaffold is first assembled (*4*, *50*, *51*). BM regulation during tissue growth, shaping, remodeling, and homeostasis, however, is less well understood (*52*). By constructing domain-based networks, we identified conserved candidate regulatory hub proteins, of which the HSPG2 shares the most domains with other BM zone proteins. We found a role for perlecan late in *C. elegans* larval development in maintaining gonadal tissue shape. Perlecan loss in *Drosophila* also results in tissue deformation (*28*, *29*), suggesting tissue sculpting is a general perlecan function. In addition, we found that perlecan loss resulted in fibrillar and globular BM aggregates. Given perlecan’s many shared domains, it might help maintain tissue form, in part, by connecting BM constituents. Other BM regulatory hub candidates include signaling molecules, proteases, and heparan sulfate proteoglycans. Some of these have known postembryonic roles in BM growth [e.g., GON-1 (ADAMTS9) and MIG-6 (PAPLN)] (*21*, *53*), and we establish here an unexpected role for SAX-3/ROBO in regulating BM composition. Thus, network connectivity analysis provides a new approach to uncover potential BM regulatory proteins.

To further investigate mechanisms of postembryonic BM regulation, we performed a comprehensive RNAi screen of 77 BM zone genes on the growing *C. elegans* gonadal BM. Loss of ADT-2 (ADAMTS3) protease caused a sharp reduction in laminin and collagen IV levels and misshapen gonads. Moreover, depletion of ADAMTS3 in zebrafish embryos resulted in a reduction in type IV collagen levels in the cranial regions, accompanied by cerebral hemorrhage. As *ADAMTS3* variants are associated with abnormally expanded lymphatic vessels in Hennekam syndrome (*54*), ADAMTS3 might control tissue shape and integrity by regulating BM composition. We found nine genes that function to restrict BM collagen IV levels. Precise levels of type IV collagen within BMs facilitate its complex and essential functions. For example, collagen levels influence TGFβ signaling during kidney tubule growth in *Drosophila*, control tissue shaping of the *Drosophila* wing disc and egg chamber, and help constrict the mouse salivary gland (*28*, *55*–*57*). The interplay between mechanisms that add, remove, and limit collagen IV may thus allow cells and tissues to finely control BM collagen IV levels according to tissue needs. Moreover, these mechanisms might break down during senescence and disease, as altered collagen IV levels are a hallmark of aging, diabetes, and Alzheimer’s disease (*12*, *58*–*60*). We found the TGFβ pathway and ROBO restrict collagen IV accumulation in *C. elegans* and zebrafish. Collagen IV binds and regulates the ligands of these receptors (*6*, *61*). Thus, our findings indicate that interactions between these pathways and collagen IV are reciprocal and may be a mechanism to set and maintain collagen IV levels and buffer levels of signaling.

Using our BM zone gene network, we revealed human phenotype associations for more than 100 genes that affect all major organ systems, thus consolidating the growing link between BM genes and human disease (*8*). Analysis of constraint metrics in gnomAD showed that 51 BM zone genes are intolerant to pLoF variation. These include type IV collagen and laminin gamma chains, dystroglycan, and integrin subunits, components with critical roles in early BM formation and embryonic development (*2*). Several TGFβ, SLIT, and ROBO genes, which we have implicated in the regulation of BM collagen IV levels in this study, appear to be intolerant as well, suggesting that variants in these genes could be pathogenic in humans. Through genomic analyses of the 100KGP rare disease cohort, we also identified high-penetrance homozygous pLoF variants in 10 BM zone genes with limited or no previous associations to BM pathology and human disease, and we characterized *MATN2*, *MPZL2*, and *LAMA5* variants. We report a disease association for MATN2 and demonstrate that LoF variants in *MATN2* affect translation, secretion, and splicing. Biochemical analyses suggest that MATN2 interacts with many BM proteins (*62*), and we found that depletion of MATN2 affected levels of core BM components in human podocyte-derived ECM. MPZL2 and LAMA5 are BM zone proteins with limited connections to disease and BM pathology. A LoF variant in the adhesion protein MPZL2 (*MPZL2*^*c*.72delA^) was previously associated with hearing loss (*42*), and we identified a 100KGP patient with additional nervous system, cardiovascular, and kidney defects, thus extending the phenotypic spectrum of *MPZL2*^*c*.72delA^. We used the zebrafish kidney to assay BM structure and BM filtration function and found that disruption of MPZL2 resulted in thicker, irregular kidney BMs and kidney filtration defects. MPZL2 is localized to the ear BM zone (*42*), and it is likely that similar BM defects could lead to sensorineural hearing loss. Consistent with this notion, collagen IV (*COL4A3/4/5*) variants disrupt BMs and are also associated with hearing impairment (*63*). *LAMA5* is a laminin alpha chain whose knockout is embryonic lethal in mice (*64*). Two missense variants *LAMA5*^V3140M^ and *LAMA5*^R286L^ are associated with a range of human phenotypes but have not been connected to BM pathology (*45*, *46*). We identified LoF *LAMA5* variants linked to human fetal lethality and defects in kidney BMs and kidney formation. Depletion of *lama5* in zebrafish also resulted in irregular BM morphology and BM function in the kidney. Together, these findings provide compelling evidence that variants in *MATN2*, *MPZL2*, and *LAMA5* could lead to BM pathology in humans.

The network of more than 200 BM-associated components defined in this study provide BMs’ astonishing complexity, which likely underlies the diversity of BM functions that are vital to human health. The multidisciplinary investigative pipeline established here to identify BM components, sites of localization, key BM regulatory nodes, and disease association provides a framework for discovering mechanisms that dictate BM function and regulation. We expect that this will ultimately facilitate earlier disease detection, improve prognosis prediction, and inform therapies for BM-associated human disease.

## MATERIALS AND METHODS

### Generation of BM zone network

To build a comprehensive BM zone network of genes encoding BM and CSI components, we adopted the following strategy: (i) initial identification of BM zone candidates via GO, (ii) expansion of the BM gene network, and (iii) verification of BM zone localization for candidates (detailed in Supplementary Text). Briefly, to obtain a primary list of candidate genes, we used the GO Resource [release 2020-07 (*23*)] to search for human genes cataloged under the BM cellular component term (GO:0008003/GO:0005605) and retrieved 103 genes. Next, we applied a network expansion strategy to include candidate genes through (i) data curation, (ii) analysis of BM expression signature in *C. elegans*, (iii) conservation and enrichment analysis for BM protein domains, (iv) identification of nearest BM interacting neighbors, and (v) endogenous fluorescent tagging of BM zone candidates in *C. elegans* (tables S1 to S7). This expansion strategy added 160 candidates to our network. Next, we confirmed protein localization for 184 of 263 total candidates based on published immunolocalization studies in humans and other vertebrates and studies on orthologs in multiple animal model systems (table S7). We predicted localization to the BM zone for 38 candidates based on evidence for protein-protein interaction with other BM/CSI candidates and BM substrate-cleaving activity for proteases (fig. S1A). Fluorescent protein tagging studies in *C. elegans* (both in this and previous work) provided additional support for BM localization (table S5). We further categorized 41 candidates for which we could not confirm or predict BM zone localization under “insufficient evidence” and identified 16 GO misannotated genes, all of which were removed, and the final integrated verified BM zone network comprised 160 BM components and 62 CSIs. We adopted a color code to differentiate the localization evidence for the components in our network: green for confirmed localization to BM zone, magenta for predicted to localize to the BM zone, and gray for insufficient localization evidence. To curate this list, we created an open access, interactive resource: basement membraneBase (https://bmbase.manchester.ac.uk/), which details background, BM gene lists, localization data, component descriptions, human disease descriptions, experimental systems (*C. elegans*, *Drosophila*, zebrafish, mouse, and human), reagents, and protocols.

### Hierarchical clustering of gene expression and variance analysis

Baseline gene expression datasets screened for multitissue and nondisease gene expression were downloaded from Expression Atlas (*65*) [www.ebi.ac.uk/gxa/; release 31, for both human and mouse (table S9)]. Tissue expression profiles of the BM and CSI genes were clustered using hierarchical clustering on Spearman distance in R v3.6.4 (RRID:SCR_001905). We calculated gene expression variance across all tissue assays using R and binned BM and CSI genes into groups based on variance quartiles (low, medium, high, and very high; table S10).

### Construction of BM protein-protein interaction network

Curated interactions between BM zone proteins were obtained from the STRING (https://string-db.org/; version 11.0) database (*24*). We noted the CIs for all interactions, which are defined by STRING based on the quality of the interaction data (lowest confidence, <0.15; low confidence, 0.15 to 0.39; medium confidence, 0.4 to 0.69; high confidence, 0.7 to 0.9; and highest confidence, >0.9). The BM zone protein-protein interaction network was built using only high-confidence interaction data (combined score, >0.7). Cytoscape v.3.8.1 (RRID:SCR_003032) (*66*) was used to visualize the network, where nodes represent BM zone proteins and a single line connecting two nodes represents interaction between the two proteins.

### BM domain–based network analysis

To construct a domain-based BM network, we mapped the complement of putative BM protein domains obtained in the domain enrichment analysis (see Supplementary Text and table S3) onto our verified BM zone gene network using Cytoscape where nodes in the network represent BM zone proteins and a single line connecting two nodes represents a set of BM domains common to both. We used the NetworkAnalyzer plugin for Cytoscape v.2.8 (*67*) to compute two network topological parameters to reveal potential organization and interaction of BM zone proteins: network degree and betweenness centrality. The network degree score measures the number of connections for a given node (i.e., lines radiating out of the node) and indicates the number of proteins in the network that share one or more domains with a given protein. Biologically, a high network degree score could indicate the different roles of a BM zone protein. The betweenness centrality score measures how central a given node is based on the extent of the shortest paths across the network that pass through that node. Thus, a protein with a high betweenness centrality score may contain domains typically found in distinct subnetworks, suggesting that the protein could bridge interactions between diverse groups of BM zone proteins.

### *C. elegans* and zebrafish strains

*C. elegans* and zebrafish strains used in this study are listed in table S19 and are available upon request. Worms were reared at 20°C on nematode growth medium plates seeded with OP50 *Escherichia coli* according to standard procedures (*68*). Zebrafish were maintained and staged according to established protocols (*69*) and in accordance with the project license P1AE9A736 under the current guidelines of the U.K. Animals Act 1986. Embryos were collected from group-wise matings of y-crystallin:mcherry/l-fabp10:NL-D3 fish.

### CRISPR-Cas9 endogenous fluorescent tagging in *C. elegans*

To generate endogenous mNG or mRuby2 tags for 17 BM and 8 CSI candidate genes in *C. elegans*, we used CRISPR-Cas9–mediated genome editing with a self-excising hygromycin selection cassette as described previously (*3*, *21*). Position of tags and fluorophores used for each genomic locus are detailed in fig. S2.

### RNAi in *C. elegans*

All RNAi constructs were obtained from the Vidal (*70*) and Ahringer (*71*) libraries, except for the following clones: *sax-3*, *zmp-4*, *cpi-2*, *Y64G10A.7*, *daf-1*, *daf-4*, *tig-3*, *daf-7*, *sma-6*, *smp-2*, and *mab-20*, which were constructed from polymerase chain reaction (PCR) fragments corresponding to the longest transcripts of the respective genes, as described previously (*3*). RNAi experiments were performed using the feeding method and according to protocols detailed by Jayadev *et al.*, (*3*). RNAi was initiated in synchronized L1 larvae, and animals were fed for 24 to 72 hours at 20°C depending on the experiment. For the RNAi screen, we first assessed laminin (LAM-2::mNG) and type IV collagen (EMB-9::mRuby2) gonadal BM fluorescence levels visually. Quantifications were then performed for all knockdown conditions other than positive controls where visible changes in fluorescence intensity were observed. At least 20 animals each were examined for both visual and quantitative assessments of BM fluorescence.

### CRISPR-Cas9 knockdown in zebrafish

CRISPR-Cas9 knockdown was performed as previously described (*30*). Four guide RNAs (gRNAs) for each gene (table S20) were used to generate crispant embryos with knockout-like phenotypes in the *F*_0_ generation. gRNAs (Merck) were resuspended to 20 μM and aliquoted for storage. On the day of injection, the ribonucleoprotein injection mix [5 μM EnGen Spy Cas9 NLS (New England BioLabs, M0646T) mixed with 2.5 μM of each gRNA and Cas9 buffer] was freshly prepared, and 5.0 nl was injected into one-cell stage wild-type zebrafish embryos, which were then maintained and processed for further analyses when they reached the required stage of development.

### Kidney filtration assay in zebrafish

The *NL:D3* transgenic zebrafish (*33*) were injected at the one-cell stage with gRNAs targeting *adamts3* or *robo1* (table S20) and Cas9 protein. Crispant *NL:D3* embryos were grown to 4 days postfertilization (dpf) and then placed in wells of a 96-well plate. Three embryos were placed in a single well, and 12 wells were set up per experimental sample. Embryo medium (E3) was removed and replaced with 200 μl of fresh E3. These embryos were then left for 24 hours before 50 μl of E3 was extracted from each well and placed into corresponding wells of an opaque 96-well plate. Fifty microliters of Nano-Glo Luciferase reporter buffer and substrate (Promega, N1120) was added to the cultured E3 medium. The 96-well plate was spun at 700 rpm for 1 min before luminescence intensity was determined on a FlexStation 3 multimode microplate reader (Molecular Devices).

### TGFBR1 inhibition

Zebrafish embryos were grown to 24 hours postfertilization (hpf) and manually dechorionated. Embryos were then placed in 50 μM SB431542 (Calbiochem, 616461) or 0.1% dimethyl sulfoxide vehicle control and cultured for a further 48 hours with a change in media (containing drug or vehicle) after 24 hours. Embryos were fixed in 4% paraformaldehyde (PFA) at 72 hpf.

### Human tissue samples

Postmortem fetal tissue from the *LAMA5* cases was used with prior parental consent for use in research. The control 21-week human fetal kidney sections were acquired in a previous study (*72*) and were initially provided by the Joint MRC/Wellcome Trust Human Developmental Biology Resource (http://hdbr.org; REC reference: 08/H0712/34+5). All experimental protocols were approved by the institute’s Ethical Committee (reference 010/H0713/6) and performed in accordance with institutional ethical and regulatory guidelines. We were unable to obtain further clinical details or tissue samples from the reported individuals with *MPZL2* and *MATN2* variants.

### Histological staining and immunofluorescence

For histological staining of human fetal kidneys, formalin-fixed paraffin-embedded (FFPE) human fetal kidney sections were dewaxed using xylene and hydrated using a graded series of ethanol solutions (100 to 70%) to water. The slides were then stained using Picrosirius red with Fast Green counterstain for 1 hour and washed with 1% acetic acid. Slides were then dehydrated using ethanol and xylene before coverslipping.

For pan-laminin and LAMB2 immunofluorescence, FFPE human fetal kidney sections were dewaxed, hydrated, and heated in 10 mM sodium citrate buffer (pH 9.0) for 15 min for antigen retrieval. After blocking with 1% bovine serum albumin (BSA) [prepared in phosphate-buffered saline (PBS)] for 1 hour at room temperature, sections were stained with a rabbit anti-laminin (1:200 dilution; Abcam, ab11575, RRID:AB_298179) and mouse anti-S/β2 (1:50 dilution; clone CL2979; Novus Biologicals, NBP-42387) antibodies overnight at 4°C. For type IV collagen immunofluorescence in zebrafish, 5-dpf zebrafish embryos were fixed in 4% PFA overnight at 4°C and embedded in optimal cutting temperature (OCT) compound. Cryosections (thickness, 5 μm) were air-dried, permeabilized with acetone at −20°C for 8 min, blocked with 10% FBS and 5% donkey serum in PBS–Triton X for 1 hour at room temperature, and stained with a rabbit anti–type IV collagen (1:250 dilution; Abcam, ab6586, RRID:AB_305584) overnight at 4°C. For type IV collagen and nidogen immunofluorescence analysis of podocyte matrix, proliferating AB podocytes (wild type and *MATN2* knockdown) were fixed with 4% PFA for 20 min at room temperature, blocked with 1% BSA for 1 hour at room temperature, and stained overnight at 4°C with rabbit anti–collagen IV (1:100 dilution) and mouse anti-nidogen (1:100 dilution; Invitrogen, 302117, RRID:AB_2609420) antibodies. Species-specific secondary antibodies conjugated with either Alexa Fluor 488 (1:400 dilution; Invitrogen Antibodies, A11008, RRID:AB_143165), Alexa Fluor 594 (1:400 dilution; Invitrogen Antibodies, A21203, RRID:AB_141633), or Alexa Fluor 647 (1:400 dilution; Invitrogen Antibodies, A-21235, RRID:AB_2535804) were used, and the slides mounted with ProLong Diamond antifade (Thermo Fisher Scientific, P36961).

### Microscopy and image processing

For *C. elegans* experiments, fluorescence images were acquired at 20°C on an Axio Imager A1 microscope (Carl Zeiss) controlled by the μManager software v.1.4.23 (RRID:SCR_016865) (*73*) and equipped with an electron multiplying charge-coupled device camera (Hamamatsu Photonics), a 40× Plan Apochromat (1.4 numerical aperture) objective, a spinning disc confocal scan head (CSU-10, Yokogawa Electric Corp.), and 488- and 561-nm laser lines. Worms were mounted on 5% noble agar pads containing 0.01 M sodium azide for imaging. For the BM zone gene tagging experiments and the RNAi screen, we captured single-slice images at the middle focal plane of animals where most or all the gonadal and pharyngeal tissue cross sections were in focus. For the perlecan knockdown experiments, we acquired *z*-stacks at 0.37-μm intervals spanning the surface of the gonad and generated sum projections. All quantifications of mean fluorescence intensity were done on raw images using the Fiji/ImageJ software v.2.0 (RRID:SCR_002285) (*74*). We drew ∼30-pixel-long line scans along the BM to obtain raw values of mean fluorescence intensity. Background intensity values were obtained by averaging two lines scans of similar length in regions adjacent to the BM with no visible fluorescence signal.

For zebrafish experiments, images were collected on a Leica TCS SP8 acousto-optical beam splitter (AOBS) inverted confocal using a 20×/0.50 Plan Fluotar objective and ×3 confocal zoom. The confocal settings were as follows: pinhole, 1 airy unit; scan speed, 1000 Hz unidirectional; format, 1024 × 1024. Images were collected using hybrid detectors with the following detection mirror settings: Alexa Fluor 494 to 530 nm using the white light laser with 488-nm (10%) laser line. For quantification of fluorescence, we obtained raw integrated density measurements for the whole field of view in Fiji/ImageJ. Bright-field images of zebrafish embryos were acquired using a Leica M205 FA upright stereofluorescence microscope. For electron microscopy, samples were prepared according to protocols described previously (*75*). Images were taken on T12 BioTwin transmission electron microscope. Distances were measured in Fiji/ImageJ using grid method measurements and normalized to the length of the glomerular BM.

For human histological staining and immunofluorescence, slides were imaged using a 3DHISTECH Pannoramic 250 microscope slide scanner. Fluorescence images were acquired using a PCO.edge camera with a ×20/0.80 Plan Apochromat objective (Zeiss) and the fluorescein isothiocyanate (FITC) and Texas Red filter sets. Transmitted light images were acquired using a CIS VCC-FC60FR19CL camera with a ×20/0.80 Plan Apochromat objective (Zeiss). Snapshots of the slide scans were taken using the Case Viewer software v.2.4.0.119028 (3DHISTECH). Slides were imaged using a 3DHISTECH Pannoramic 250 slide scanner microscopy. For human podocyte experiments, fluorescence images were collected on a Zeiss Axioimager.D2 upright microscope using a ×40/0.75 EC Plan Neofluar objective and captured using a CoolSNAP HQ2 camera (Photometrics) through the μManager software v1.4.23. Specific band-pass filter sets for 4′,6-diamidino-2-phenylindole, FITC, and Far red (Alexa Fluor 647) were used to prevent bleed-through from one channel to the next. Images were then processed and analyzed using Fiji/ImageJ.

For immunofluorescence analysis of podocyte matrix, images were acquired with a Leica TCS SP8 AOBS inverted confocal microscope using hybrid detectors. When acquiring three-dimensional (3D) optical stacks, the confocal software was used to determine the optimal number of *z* sections. 3D image stacks were analyzed with Fiji/ImageJ. Collagen IV and nidogen deposition patterns were quantified with the TWOMBLI Fiji macro (*76*). We used three metrics: (i) the proportion of high-density matrix (% HDM), which indicates the proportion of pixels in a given image that corresponds to immunofluorescence signal within the podocyte matrix; (ii) area of the HDM; and (iii) matrix fiber thickness, which is derived from the area measurement and the length of fibers.

### Human disease phenotypes

We manually searched for each of the BM and CSI genes within the following databases: HPO database (https://hpo.jax.org/app/) (*35*), the Genomics England PanelApp (https://panelapp.genomicsengland.co.uk) (*36*), and the OMIM database (McKusick-Nathans Institute of Genetic Medicine, Johns Hopkins University Baltimore, MD; https://omim.org/). Top-level HPO terms associated with each OMIM disease entry listed were grouped under individual BM genes.

### Genomic analysis

We examined constraint metrics for pLoF variation for the verified BM zone gene network within the gnomAD (gnomAD v2.1.1) (*38*). Two metrics were used: the pLI score and the LOEUF score. The pLI score is a measure of the tolerance of a gene to pLoF, taking into account the number of pLoF variants found in control databases for that gene. The pLI score is weighted by gene sequence coverage and gene size, and a score close to >0.9 suggests intolerance to pLoF variants. The LOEUF score is an estimate of the observed/expected ratio of pLoF variants based on the upper bound of a Poisson-derived CI around the ratio. A LOEUF score of <0.2 suggests that a gene is intolerant to pLoF variants.

We screened whole-genome sequencing data from the Genomics England 100KGP (*22*) for variants in the verified BM zone gene network. The project was formally registered and approved (Genomics England Research Registry, RR320). We extracted all BM and CSI genomic variants from an aggregated set of variants available for 63,039 individuals recruited to the rare disease arm of the 100KGP; this data is composed of 34,842 individuals affected with a rare disease across broad disease areas and 28,197 unaffected relatives at the time of recruitment. The minor allele frequency was calculated from Genomics England aggregated variant calling format, and pLoF variants were identified after annotation through Ensembl variant effect predictor to a predefined list of transcripts for the BM and CSI gene network. Variants matching one or more of the following criteria were included: stop_lost, start_lost, stop_gained, transcript_ablation, splice_acceptor_variant, splice_donor_variant, and frameshift_variant. Gene burden testing was performed using Test Rare Variants with Public Data (*77*) and identified only modest signals for previously known disease-causing genes under dominant and recessive models. Because of the heterogeneous nature of the cohort, we used a logical filtering strategy to identify rare/new pLoF variants enriched in affected individuals under the assumption of high penetrance: ≤1 homozygous pLoF variant affecting canonical transcript in gnomAD v2.1 with the high-confidence LoF flag and no individuals with homozygous pLoF variants in unaffected 100KGP cohort; genes with multiple affected individuals were prioritized. Genes fitting these criteria were further evaluated for individuals carrying heterozygous pLoF variants in-trans to another potential damaging mutation; these were highlighted through Exomiser (*78*) results available in the Genomics England research embassy. Statistical analyses and graphics were created in R software. We report OR and *P* values from the Fisher exact test using the oddsratio function in the “epitools” package for R. Case histories for individuals carrying *MATN2*, *MPZL2*, or *LAMA5* variants are provided in Supplementary Text.

### Minigene splicing assay

Minigene vectors for both wild-type and variant sequences for *MATN2* and *LAMA5* were assembled using the SK3 minigene vector (a derivative of the pSpliceExpress minigene splice reporter vector, gifted by S. Stamm; Addgene, 32485, RRID:Addgene_32485) as previously described (*40*). Human embryonic kidney 293 cells were cultured to 40 to 60% confluency in 2 ml of high-glucose Dulbecco’s modified Eagle’s medium (Sigma-Aldrich), supplemented with 10% fetal bovine serum (Sigma-Aldrich) in a six-well cell culture plate at 37°C with 5% CO_2_. Cells were transiently transfected with 2.5 μg of minigene vector using Lipofectamine 3000/LXT (Thermo Fisher Scientific) using the manufacturer’s protocol. Following 18-hour incubation at 37°C with 5% CO_2_, RNA was extracted using 1 ml of TRI Reagent (Sigma-Aldrich) per well and further purified using an RNeasy cleanup kit (QIAGEN). An equal amount of RNA for each sample was converted to cDNA using the PrimeScript RT Reagent Kit (Takara Bio) or Superscript IV (Thermo Fisher Scientific). cDNA for *MATN2* and *LAMA5* minigenes was amplified using Q5 (New England BioLabs) or KOD polymerase with the following forward and reverse primers: 5′-GCACCTTTGTGGTTCTCACT-3′ and 5′-GGGCCTAGTTGCAGTAGTTCT-3′. Last, PCR products were run on an agarose gel (1 to 4%) supplemented with SYBR Safe (Thermo Fisher Scientific) for visualization on an ultraviolet transilluminator. PCR products were purified using a QIAquick PCR purification kit (QIAGEN, 28104) and sequenced by Sanger sequencing (Eurofins Genomics).

### 3D structure prediction

The MATN2 (O00339) protein sequence was obtained from the UniProtKB database. EGF1 domain (amino acids 238 to 278) sequences of either the wild-type MATN2 or MATN2 variant 3a were submitted to the Phyre2 web portal to generate 3D models (*79*). Templates (c1lpkA for MATN2_EGF1) were selected on the basis of maximum identity and query coverage. Visualization of the variant EGF domain was performed using PyMOL v.1.2r3pre (Schrödinger LLC, RRID:SCR_000305).

### *MATN2* gene knock down and rescue

A *MATN2* knockdown line was generated in human immortalized podocytes (RRID:CVCL_W186) by CRISPR-Cas9. Cas9 expressing podocytes were established by transduction with pLentiCas9_v2 (Addgene, plasmid 52961), followed by puromycin selection. These Cas9 podocytes were transfected twice with two single gRNA targeting *MATN2* (5′-GTCACGATCATTATGACCCG-3′ and 5′-CTTGACCTTTGCATAGTCAT-3′; Merck) using RNAiMAX (Thermo Fisher Scientific) in a six-well plate and depletion of MATN2 confirmed by Western blot. Lentiviral vectors expressing *MATN2* coding sequences (wild type, variant 2 or 3a) under the control of the human elongation factor 1 alpha (EF-1a) promoter were designed, cloned, and packaged (VectorBuilder). Vector sequences are available on Addgene and on figshare: 10.6084/m9.figshare.c.5662348. *MATN2*-KD podocytes (10^6^) were seeded in 10-cm dishes and transduced; briefly, to 964 μl of serum-free media, 16 μl of polybrene (stock, 5 mg/ml), and 20 μl of 3.78 × 10^8^ TU/ml virus were added and incubated for 6 hours. The virus was then removed; cells were washed, and fresh complete RPMI 1640 medium was added. Stably transduced cells were differentiated for 8 days before harvesting cell lysate, media, and deposited ECM. Western blotting was performed using 4 to 12% bis-tris gradient gels with the MES buffer system according to the manufacturer’s instructions (Life Sciences). Reduced protein samples were transferred to nitrocellulose membranes and then blotted using an anti-MATN2 antibody (1:1000 dilution; Proteintech Ltd., 24064-1-AP, RRID:AB_2879422) with anti-rabbit 680 conjugate (1:10,000 dilution; the Jackson Laboratory, 111-625-144, RRID: AB_2338085) as a secondary antibody. Protein bands were visualized using the Odyssey Imaging System (LI-COR) at 700 nm. For PCR amplification of the V5 tag, cDNA was extracted from cells and a PCR was performed using a QIAquick PCR purification kit (QIAGEN) and the following primer sequences: forward, 5′-GCCTATCCCTAACCCTCTCC-3′ and reverse, 5′CATTCTTGACAGTGCTGCCA-3′.

### Sample preparation for mass spectrometry

Three clones were generated from a pool of a human podocyte cells that were knocked down for *MATN2*. The cells were then diluted and plated up in 96-well plate to obtain colonies from a single cell (pure population of cells with the same phenotype, i.e., *MATN2*-KD). Human immortalized podocytes (wild type and the three *MATN2*-KD clones) (*39*) were cultured for 14 days at 37°C in monoculture on uncoated 10-cm dishes using RPMI 1640 medium (Sigma-Aldrich, R-8758) supplemented with 10% FBS (Life Technologies), 1% insulin, transferrin, selenium (Sigma-Aldrich, I-1184), and 1% penicillin-streptomycin. Samples were enriched for ECM proteins as previously described (*39*). Briefly, cells and matrix were scraped into lysis buffer (10 mM tris, 150 mM NaCl, 1% Triton X-100, 25 mM EDTA, and protease inhibitor) and incubated for 1 hour, followed by centrifugation at 14,000*g* for 10 min. The supernatant (lysate) was kept and added with 5% SDS with 50 mM triethylammonium bicarbonate (TEAB). The pellet was resuspended in alkaline detergent buffer (20 mM NH_4_OH and 0.5% Triton X-100 in PBS) for 30 min to disrupt cell-matrix bonds. Following centrifugation, the remaining pellet enriched for ECM proteins was resuspended in 5% SDS with 50 mM TEAB.

The lysate and the ECM-rich fractions were processed for in-solution digestion as described previously (*72*). To extract proteins, samples were lysed using a Covaris LE220+ Focused Ultrasonicator (Covaris). The protein samples were reduced with 5 mM dithiothreitol for 10 min at 60°C and alkylated with 15 mM iodoacetamide for 30 min in the dark. Protein concentration was quantified using a Direct Detect spectrometer (Millipore, DDHW00010-WW), and 40 μg of the proteins/sample was loaded onto S-Trap columns (ProtiFi) and digested overnight with 1 μg of sequencing-grade modified trypsin (Promega, V5111) at 37°C. Peptides were eluted from the column with 30% acetonitrile and 0.1% formic acid aqueous solution and collected by centrifugation at 4000*g* for 2 min. After desalting, the peptide samples were dried to completeness by vacuum centrifugation and submitted to analysis by mass spectrometry.

### Mass spectrometry data acquisition and analysis

Samples were analyzed by liquid chromatography–tandem mass spectrometry using a Thermo Exploris 480 mass spectrometer (Thermo Fisher Scientific). Mass spectrometry analysis was carried out using Proteome Discoverer v.2.4 SP1 (Thermo Fisher Scientific, RRID:SCR_014477). Raw spectra data were searched against human Swiss-Prot and TrEMBL (v.2017-10-25) using the SEQUEST HT search tool. Tryptic peptides with up to two missed cleavage sites and mass tolerance of 10 parts per million for precursor ions and 0.02 Da for fragment ions were set for the search. Carbamidomethylation of cysteine was selected as fixed modification and oxidation of proline, lysine, and methionine and N-terminal acetylation as dynamic modifications. Protein false discovery rate was set to 1%, and protein validation was performed using the target/decoy strategy. Protein abundances were determined by label-free quantification based on precursor ion intensity. Results were filtered for significant false discovery rate (>0.1) master proteins identified with >1 unique peptide.

### Statistical analysis

For *C. elegans*, zebrafish, and human podocyte experiments, statistical analysis was performed in GraphPad Prism v7 (RRID:SCR_002798). Sample sizes were validated a posteriori through assessments of normality by log-transforming all datasets and then using the Shapiro-Wilk test. For comparisons of mean fluorescence intensities between two populations, we used an unpaired two-tailed Student’s *t* test (with Welch’s correction in cases of unequal variance between samples). To compare mean fluorescence intensities between three or more populations, we performed one-way analysis of variance (ANOVA), followed by a post hoc Dunnett’s multiple comparisons test. For OR comparisons in genomic analyses, we performed the Fisher’s exact test in R software (RRID:SCR_001905). Boxplots were prepared in GraphPad Prism v7. Figure legends indicate sample sizes (*n*), statistical tests used, and *P* values.

## Supplementary Materials

### This PDF file includes:

Supplementary Text Genomics England Research Consortium Index for tables S1 to S20 Figs. S1 to S11 References

## Notes

View/request a protocol for this paper from *Bio-protocol*.

## References

[1] A. Pozzi, P. D. Yurchenco, R. V. Iozzo, The nature and biology of basement membranes. Matrix Biol. 57–58, 1–11 (2017). 10.1016/j.matbio.2016.12.009 PMC5387862 28040522

[2] P. D. Yurchenco, Basement membranes: Cell scaffoldings and signaling platforms. Cold Spring Harb. Perspect. Biol. 3, a004911 (2011). 21421915 10.1101/cshperspect.a004911 PMC3039528

[3] R. Jayadev, Q. Chi, D. P. Keeley, E. L. Hastie, L. C. Kelley, D. R. Sherwood, α-Integrins dictate distinct modes of type IV collagen recruitment to basement membranes. J. Cell Biol. 218, 3098–3116 (2019). 31387941 10.1083/jcb.201903124 PMC6719451

[4] S. Li, Y. Qi, J. Liu, K. Mckee, Integrin and dystroglycan compensate each other to mediate laminin-dependent basement membrane assembly and epiblast polarization. Matrix Biol. 57-58, 272–284 (2017). 27449702 10.1016/j.matbio.2016.07.005 PMC5250580

[5] R. Jayadev, D. R. Sherwood, Basement membranes. Curr. Biol. 27, R207–R211 (2017). 28324731 10.1016/j.cub.2017.02.006

[6] X. Wang, R. E. Harris, L. J. Bayston, H. L. Ashe, Type IV collagens regulate BMP signalling in *Drosophila*. Nature 455, 72–77 (2008). 18701888 10.1038/nature07214

[7] D. R. Sherwood, Basement membrane remodeling guides cell migration and cell morphogenesis during development. Curr. Opin. Cell Biol. 72, 19–27 (2021). 34015751 10.1016/j.ceb.2021.04.003 PMC8530833

[8] A. Nyström, O. Bornert, T. Kühl, Cell therapy for basement membrane-linked diseases. Matrix Biol. 57–58, 124–139 (2017). 10.1016/j.matbio.2016.07.012 27609402

[9] M. H. Foster, Basement membranes and autoimmune diseases. Matrix Biol. 57–58, 149–168 (2017). 10.1016/j.matbio.2016.07.008 PMC5290253 27496347

[10] A. Naba, K. R. Clauser, C. A. Whittaker, S. A. Carr, K. K. Tanabe, R. O. Hyne, Extracellular matrix signatures of human primary metastatic colon cancers and their metastases to liver. BMC Cancer 14, 518 (2014). 25037231 10.1186/1471-2407-14-518 PMC4223627

[11] E. C. Tsilibary, Microvascular basement membranes in diabetes mellitus. J. Pathol. 200, 537–546 (2003). 12845621 10.1002/path.1439

[12] M. Randles, F. Lausecker, Q. Kong, H. Suleiman, G. Reid, M. Kolatsi-Joannou, P. Tian, S. Falcone, B. Davenport, P. Potter, T. Van Agtmael, J. Norman, D. Long, M. Humphries, J. Miner, R. Lennon, Identification of an altered matrix signature in kidney aging and disease. J. Am. Soc. Nephrol. 32, 1713–1732 (2021). 34049963 10.1681/ASN.2020101442 PMC8425653

[13] M. J. Randles, M. J. Humphries, R. Lennon, Proteomic definitions of basement membrane composition in health and disease. Matrix Biol. 57–58, 12–28 (2017). 10.1016/j.matbio.2016.08.006 27553508

[14] A. C. Teuscher, E. Jongsma, M. N. Davis, C. Statzer, J. M. Gebauer, A. Naba, C. Y. Ewald, The in-silico characterization of the *Caenorhabditis elegans* matrisome and proposal of a novel collagen classification. Matrix Biol. Plus 1, 100001 (2019). 33543001 10.1016/j.mbplus.2018.11.001 PMC7852208

[15] R. O. Hynes, A. Naba, Overview of the matrisome—An inventory of extracellular matrix constituents and functions. Cold Spring Harb. Perspect. Biol. 4, a004903 (2012). 21937732 10.1101/cshperspect.a004903 PMC3249625

[16] P. Nauroy, S. Hughes, A. Naba, F. Ruggiero, The in-silico zebrafish matrisome: A new tool to study extracellular matrix gene and protein functions. Matrix Biol. 65, 5–13 (2018). 28739138 10.1016/j.matbio.2017.07.001

[17] H. Suleiman, L. Zhang, R. Roth, J. E. Heuser, J. H. Miner, A. S. Shaw, A. Dani, Nanoscale protein architecture of the kidney glomerular basement membrane. eLife 2, e01149 (2013). 24137544 10.7554/eLife.01149 PMC3790497

[18] M. Uhlén, L. Fagerberg, B. M. Hallström, C. Lindskog, P. Oksvold, A. Mardinoglu, Å. Sivertsson, C. Kampf, E. Sjöstedt, A. Asplund, I. M. Olsson, K. Edlund, E. Lundberg, S. Navani, C. A.-K. Szigyarto, J. Odeberg, D. Djureinovic, J. O. Takanen, S. Hober, T. Alm, P.-H. Edqvist, H. Berling, H. Tegel, J. Mulder, J. Rockberg, P. Nilsson, J. M. Schwenk, M. Hamsten, K. von Feilitzen, M. Forsberg, L. Persson, F. Johansson, M. Zwahlen, G. von Heijne, J. Nielsen, F. Pontén, Proteomics. Tissue-based map of the human proteome. Science 347, 1260419 (2015). 25613900 10.1126/science.1260419

[19] K. Tsutsui, H. Machida, A. Nakagawa, K. Ahn, R. Morita, K. Sekiguchi, J. H. Miner, H. Fujiwara, Mapping the molecular and structural specialization of the skin basement membrane for inter-tissue interactions. Nat. Commun. 12, 2577 (2021). 33972551 10.1038/s41467-021-22881-y PMC8110968

[20] R. Manabe, K. Tsutsui, T. Yamada, M. Kimura, I. Nakano, C. Shimono, N. Sanzen, Y. Furutani, T. Fukuda, Y. Oguri, K. Shimamoto, D. Kiyozumi, Y. Sato, Y. Sado, H. Senoo, S. Yamashina, S. Fukuda, J. Kawai, N. Sugiura, K. Kimata, Y. Hayashizaki, K. Sekiguchi, Transcriptome-based systematic identification of extracellular matrix proteins. Proc. Natl. Acad. Sci. U.S.A. 105, 12849–12854 (2008). 18757743 10.1073/pnas.0803640105 PMC2529034

[21] D. P. Keeley, E. Hastie, R. Jayadev, L. C. Kelley, Q. Chi, S. G. Payne, J. L. Jeger, B. D. Hoffman, D. R. Sherwood, Comprehensive endogenous tagging of basement membrane components reveals dynamic movement within the matrix scaffolding. Dev. Cell 54, 60–74.e7 (2020). 32585132 10.1016/j.devcel.2020.05.022 PMC7394237

[22] M. Caulfield, J. Davies, M. Dennys, L. Elbahy, T. Fowler, S. Hill, T. Hubbard, L. Jostins, N. Maltby, J. Mahon-Pearson, G. M. Vean, K. Nevin-Ridley, M. Parker, V. Parry, A. Rendon, L. Riley, C. Turnbull, K. Woods, The national genomics research and healthcare knowledgebase. Figshare 10.6084/m9.figshare.4530893.v5 (2019).

[23] The Gene Ontology Consortium, The gene ontology resource: Enriching a gold mine. Nucleic Acids Res. 49, D325–D334 (2021). 33290552 10.1093/nar/gkaa1113 PMC7779012

[24] D. Szklarczyk, A. L. Gable, D. Lyon, A. Junge, S. Wyder, J. Huerta-Cepas, M. Simonovic, N. T. Doncheva, J. H. Morris, P. Bork, L. J. Jensen, C. von Mering, STRING v11: Protein-protein association networks with increased coverage, supporting functional discovery in genome-wide experimental datasets. Nucleic Acids Res. 47, D607–D613 (2019). 30476243 10.1093/nar/gky1131 PMC6323986

[25] M. A. Wouters, I. Rigoutsos, C. K. Chu, L. L. Feng, D. B. Sparrow, S. L. Dunwoodie, Evolution of distinct EGF domains with specific functions. Protein Sci. 14, 1091–1103 (2005). 15772310 10.1110/ps.041207005 PMC2253431

[26] M. Costell, E. Gustafsson, A. Aszódi, M. Mörgelin, W. Bloch, E. Hunziker, K. Addicks, R. Timpl, R. Fässler, Perlecan maintains the integrity of cartilage and some basement membranes. J. Cell Biol. 147, 1109–1122 (1999). 10579729 10.1083/jcb.147.5.1109 PMC2169352

[27] T. M. Rogalski, B. D. Williams, G. P. Mullen, D. G. Moerman, Products of the unc-52 gene in *Caenorhabditis elegans* are homologous to the core protein of the mammalian basement membrane heparan sulfate proteoglycan. Genes Dev. 7, 1471–1484 (1993). 8393416 10.1101/gad.7.8.1471

[28] J. C. Pastor-Pareja, T. Xu, Shaping cells and organs in *Drosophila* by opposing roles of fat body-secreted collagen IV and perlecan. Dev. Cell 21, 245–256 (2011). 21839919 10.1016/j.devcel.2011.06.026 PMC4153364

[29] A. J. Isabella, S. Horne-Badovinac, Dynamic regulation of basement membrane protein levels promotes egg chamber elongation in *Drosophila*. Dev. Biol. 406, 212–221 (2015). 26348027 10.1016/j.ydbio.2015.08.018 PMC4639450

[30] R. S. Wu, I. I. Lam, H. Clay, D. N. Duong, R. C. Deo, S. R. Coughlin, A rapid method for directed gene knockout for screening in G0 zebrafish. Dev. Cell 46, 112–125.e4 (2018). 29974860 10.1016/j.devcel.2018.06.003

[31] T. J. Carney, N. M. Feitosa, C. Sonntag, K. Slanchev, J. Kluger, D. Kiyozumi, J. M. Gebauer, J. C. Talbot, C. B. Kimmel, K. Sekiguchi, R. Wagener, H. Schwarz, P. W. Ingham, M. Hammerschmidt, Genetic analysis of fin development in zebrafish identifies furin and hemicentin1 as potential novel fraser syndrome disease genes. PLOS Genet. 6, e1000907 (2010). 20419147 10.1371/journal.pgen.1000907 PMC2855323

[32] J. H. Suh, J. H. Miner, The glomerular basement membrane as a barrier to albumin. Nat. Rev. Nephrol. 9, 470–477 (2013). 23774818 10.1038/nrneph.2013.109 PMC3839671

[33] R. W. Naylor, E. Lemarie, A. J.-Crawford, J. Bernard Davenport, A. Mironov, M. Lowe, R. Lennon, A novel nanoluciferase transgenic reporter to measure proteinuria in zebrafish. bioRxiv 10.1101/2021.07.19.452884 [**Preprint**]. 2021. PMC7614274 35716957

[34] J. B. Skeath, B. A. Wilson, S. E. Romero, M. J. Snee, Y. Zhu, H. Lacin, The extracellular metalloprotease AdamTS-A anchors neural lineages in place within and preserves the architecture of the central nervous system. Development 144, 3102–3113 (2017). 28760813 10.1242/dev.145854 PMC5611953

[35] S. Köhler, M. Gargano, N. Matentzoglu, L. C. Carmody, D. L.-Smith, N. A. Vasilevsky, D. Danis, G. Balagura, G. Baynam, A. M. Brower, T. J. Callahan, C. G. Chute, J. L. Est, P. D. Galer, S. Ganesan, M. Griese, M. Haimel, J. Pazmandi, M. Hanauer, N. L. Harris, M. J. Hartnett, M. Hastreiter, F. Hauck, Y. He, T. Jeske, H. Kearney, G. Kindle, C. Klein, K. Knoflach, R. Krause, D. Lagorce, J. A. Mc Murry, J. A. Miller, M. C. M.-Torres, R. L. Peters, C. K. Rapp, A. M. Rath, S. A. Rind, A. Z. Rosenberg, M. M. Segal, M. G. Seidel, D. Smedley, T. Talmy, Y. Thomas, S. A. Wiafe, J. Xian, Z. Yüksel, I. Helbig, C. J. Mungall, M. A. Haendel, P. N. Robinson, The human phenotype ontology in 2021. Nucleic Acids Res. 49, D1207–D1217 (2021). 33264411 10.1093/nar/gkaa1043 PMC7778952

[36] A. R. Martin, E. Williams, R. E. Foulger, S. Leigh, L. C. Daugherty, O. Niblock, I. U. S. Leong, K. R. Smith, O. Gerasimenko, E. Haraldsdottir, E. Thomas, R. H. Scott, E. Baple, A. Tucci, H. Brittain, A. de Burca, K. Ibañez, D. Kasperaviciute, D. Smedley, M. Caulfield, A. Rendon, E. M. McDonagh, PanelApp crowdsources expert knowledge to establish consensus diagnostic gene panels. Nat. Genet. 51, 1560–1565 (2019). 31676867 10.1038/s41588-019-0528-2

[37] J. S. Amberger, C. A. Bocchini, F. Schiettecatte, A. F. Scott, A. Hamosh, OMIM.org: Online Mendelian Inheritance in Man (OMIM), an online catalog of human genes and genetic disorders. Nucleic Acids Res. 43, D789–D798 (2015). 25428349 10.1093/nar/gku1205 PMC4383985

[38] K. J. Karczewski, L. C. Francioli, G. Tiao, B. B. Cummings, J. Alföldi, Q. Wang, R. L. Collins, K. M. Laricchia, A. Ganna, D. P. Birnbaum, L. D. Gauthier, H. Brand, M. Solomonson, N. A. Watts, D. Rhodes, M. S.-Berk, E. M. England, E. G. Seaby, J. A. Kosmicki, R. K. Walters, K. Tashman, Y. Farjoun, E. Banks, T. Poterba, A. Wang, C. Seed, N. Whiffin, J. X. Chong, K. E. Samocha, E. Pierce-Hoffman, Z. Zappala, A. H. O’Donnell-Luria, E. V. Minikel, B. Weisburd, M. Lek, J. S. Ware, C. Vittal, I. M. Armean, L. Bergelson, K. Cibulskis, K. M. Connolly, M. Covarrubias, S. Donnelly, S. Ferriera, S. Gabriel, J. Gentry, N. Gupta, T. Jeandet, D. Kaplan, C. Llanwarne, R. Munshi, S. Novod, N. Petrillo, D. Roazen, V. Ruano-Rubio, A. Saltzman, M. Schleicher, J. Soto, K. Tibbetts, C. Tolonen, G. Wade, M. E. Talkowski; Genome Aggregation Database Consortium, B. M. Neale, M. J. Daly, D. G. MacArthur, The mutational constraint spectrum quantified from variation in 141,456 humans. Nature 581, 434–443 (2020). 32461654 10.1038/s41586-020-2308-7 PMC7334197

[39] A. Byron, M. J. Randles, J. D. Humphries, A. Mironov, H. Hamidi, S. Harris, P. W. Mathieson, M. A. Saleem, S. C. Satchell, R. Zent, M. J. Humphries, R. Lennon, Glomerular cell cross-talk influences composition and assembly of extracellular matrix. J. Am. Soc. Nephrol. 25, 953–966 (2014). 24436469 10.1681/ASN.2013070795 PMC4005312

[40] H. B. Thomas, K. A. Wood, W. A. Buczek, C. T. Gordon, V. Pingault, T. Attié-Bitach, K. E. Hentges, V. C. Varghese, J. Amiel, W. G. Newman, R. T. O’Keefe, EFTUD2 missense variants disrupt protein function and splicing in mandibulofacial dysostosis Guion-Almeida type. Hum. Mutat. 41, 1372–1382 (2020). 32333448 10.1002/humu.24027

[41] G. Ramena, Y. Yin, Y. Yu, V. Walia, R. C. Elble, CLCA2 interactor EVA1 is required for mammary epithelial cell differentiation. PLOS ONE 11, e0147489 (2016). 26930581 10.1371/journal.pone.0147489 PMC4773014

[42] M. Wesdorp, S. M.-Cuesta, T. Peters, A. M. Celaya, A. Oonk, M. Schraders, J. Oostrik, E. G.-Rosas, A. J. Beynon, B. P. Hartel, K. Okkersen, H. J. P. M. Koenen, J. Weeda, S. Lelieveld, N. C. Voermans, I. Joosten, C. B. Hoyng, P. Lichtner, H. P. M. Kunst, I. Feenstra, S. E. de Bruijn; DOOFNL Consortium, R. J. C. Admiraal, H. G. Yntema, E. van Wijk, I. del Castillo, P. Serra, I. Varela-Nieto, R. J. E. Pennings, H. Kremer, MPZL2, Encoding the epithelial junctional protein myelin protein zero-like 2, is essential for hearing in man and mouse. Am. J. Hum. Genet. 103, 74–88 (2018). 29961571 10.1016/j.ajhg.2018.05.011 PMC6037131

[43] H. Colognato, P. D. Yurchenco, Form and function: The laminin family of heterotrimers. Dev. Dyn. 218, 213–234 (2000). 10842354 10.1002/(SICI)1097-0177(200006)218:2<213::AID-DVDY1>3.0.CO;2-R

[44] L. Ritié, C. Spenlé, J. Lacroute, A.-L. B.-Bellemin, O. Lefebvre, C. B.-Feysot, B. Jost, A. Klein, C. Arnold, M. Kedinger, D. Bagnard, G. Orend, P. S.-Assmann, Abnormal Wnt and PI3Kinase signaling in the malformed intestine of lama5 deficient mice. PLOS ONE 7, e37710 (2012). 22666383 10.1371/journal.pone.0037710 PMC3364287

[45] L. K. Jones, R. Lam, K. K. Mc Kee, M. Aleksandrova, J. Dowling, S. I. Alexander, A. Mallawaarachchi, D. L. Cottle, K. M. Short, L. Pais, J. H. Miner, A. J. Mallett, C. Simons, H. M. Carthy, P. D. Yurchenco, I. M. Smyth, A mutation affecting laminin alpha 5 polymerisation gives rise to a syndromic developmental disorder. Development 147, dev189183 (2020). 32439764 10.1242/dev.189183 PMC7540250

[46] S. Sampaolo, F. Napolitano, A. Tirozzi, M. G. Reccia, L. Lombardi, O. Farina, A. Barra, F. Cirillo, M. A. B. Melone, F. Gianfrancesco, G. D. Iorio, T. Esposito, Identification of the first dominant mutation of LAMA5 gene causing a complex multisystem syndrome due to dysfunction of the extracellular matrix. J. Med. Genet. 54, 710–720 (2017). 28735299 10.1136/jmedgenet-2017-104555

[47] Y. Wu, G. Ge, Complexity of type IV collagens: From network assembly to function. Biol. Chem. 400, 565–574 (2019). 30864416 10.1515/hsz-2018-0317

[48] A. Woods, J. R. Couchman, Proteoglycan isolation and analysis. Curr. Protoc. Cell Biol. 80, e59 (2018). 29927085 10.1002/cpcb.59

[49] A. G. Campbell, L. I. Fessler, T. Salo, J. H. Fessler, Papilin: A *Drosophila* proteoglycan-like sulfated glycoprotein from basement membranes. J. Biol. Chem. 262, 17605–17612 (1987). 3320045

[50] P. D. Yurchenco, B. L. Patton, Developmental and pathogenic mechanisms of basement membrane assembly. Curr. Pharm. Des. 15, 1277–1294 (2009). 19355968 10.2174/138161209787846766 PMC2978668

[51] Y. Matsubayashi, A. Louani, A. Dragu, B. J. Sánchez-Sánchez, E. Serna-Morales, L. Yolland, A. Gyoergy, G. Vizcay, R. A. Fleck, J. M. Heddleston, T.-L. Chew, D. E. Siekhaus, B. M. Stramer, A moving source of matrix components is essential for de novo basement membrane formation. Curr. Biol. 27, 3526–3534.e4 (2017). 29129537 10.1016/j.cub.2017.10.001 PMC5714436

[52] M. A. Morrissey, D. R. Sherwood, An active role for basement membrane assembly and modification in tissue sculpting. J. Cell Sci. 128, 1661–1668 (2015). 25717004 10.1242/jcs.168021 PMC4446735

[53] Y. Kubota, K. Nagata, A. Sugimoto, K. Nishiwaki, Tissue architecture in the *Caenorhabditis elegans* gonad depends on interactions among fibulin-1, type IV collagen and the ADAMTS extracellular protease. Genetics 190, 1379–1388 (2012). 22298704 10.1534/genetics.111.133173 PMC3316650

[54] P. Brouillard, L. Dupont, R. Helaers, R. Coulie, G. E. Tiller, J. Peeden, A. Colige, M. Vikkula, Loss of ADAMTS3 activity causes Hennekam lymphangiectasia-lymphedema syndrome 3. Hum. Mol. Genet. 26, 4095–4104 (2017). 28985353 10.1093/hmg/ddx297

[55] S. Bunt, C. Hooley, N. Hu, C. Scahill, H. Weavers, H. Skaer, Hemocyte-secreted type IV collagen enhances BMP signaling to guide renal tubule morphogenesis in *Drosophila*. Dev. Cell 19, 296–306 (2010). 20708591 10.1016/j.devcel.2010.07.019 PMC2941037

[56] J. Crest, A. Diz-Muñoz, D.-Y. Chen, D. A. Fletcher, D. Bilder, Organ sculpting by patterned extracellular matrix stiffness. eLife 6, e24958 (2017). 28653906 10.7554/eLife.24958 PMC5503509

[57] J. S. Harunaga, A. D. Doyle, K. M. Yamada, Local and global dynamics of the basement membrane during branching morphogenesis require protease activity and actomyosin contractility. Dev. Biol. 394, 197–205 (2014). 25158168 10.1016/j.ydbio.2014.08.014 PMC4174355

[58] M. S. Thomsen, L. J. Routhe, T. Moos, The vascular basement membrane in the healthy and pathological brain. J. Cereb. Blood Flow Metab. 37, 3300–3317 (2017). 28753105 10.1177/0271678X17722436 PMC5624399

[59] J. H. Miner, Type IV collagen and diabetic kidney disease. Nat. Rev. Nephrol. 16, 3–4 (2020). 31728033 10.1038/s41581-019-0229-1

[60] O. Uspenskaia, M. Liebetrau, J. Herms, A. Danek, G. F. Hamann, Aging is associated with increased collagen type IV accumulation in the basal lamina of human cerebral microvessels. BMC Neurosci. 5, 37 (2004). 15387892 10.1186/1471-2202-5-37 PMC523851

[61] T. Xiao, W. Staub, E. Robles, N. J. Gosse, G. J. Cole, H. Baier, Assembly of lamina-specific neuronal connections by slit bound to type IV collagen. Cell 146, 164–176 (2011). 21729787 10.1016/j.cell.2011.06.016 PMC3136219

[62] D. Piecha, C. Wiberg, M. Mörgelin, D. P. Reinhardt, F. Deák, P. Maurer, M. Paulsson, Matrilin-2 interacts with itself and with other extracellular matrix proteins. Biochem. J. 367, 715–721 (2002). 12180907 10.1042/BJ20021069 PMC1222949

[63] D. F. Barker, S. L. Hostikka, J. Zhou, L. T. Chow, A. R. Oliphant, S. C. Gerken, M. C. Gregory, M. H. Skolnick, C. L. Atkin, K. Tryggvason, Identification of mutations in the COL4A5 collagen gene in Alport syndrome. Science 248, 1224–1227 (1990). 2349482 10.1126/science.2349482

[64] J. H. Miner, J. Cunningham, J. R. Sanes, Roles for laminin in embryogenesis: Exencephaly, syndactyly, and placentopathy in mice lacking the laminin alpha5 chain. J. Cell Biol. 143, 1713–1723 (1998). 9852162 10.1083/jcb.143.6.1713 PMC2132973

[65] I. Papatheodorou, P. Moreno, J. Manning, A. M.-P. Fuentes, N. George, S. Fexova, N. A. Fonseca, A. Füllgrabe, M. Green, N. Huang, L. Huerta, H. Iqbal, M. Jianu, S. Mohammed, L. Zhao, A. F. Jarnuczak, S. Jupp, J. Marioni, K. Meyer, R. Petryszak, C. A. P. Medina, C. T.-López, S. Teichmann, J. A. Vizcaino, A. Brazma, Expression atlas update: From tissues to single cells. Nucleic Acids Res. 48, D77–D83 (2020). 31665515 10.1093/nar/gkz947 PMC7145605

[66] P. Shannon, A. Markiel, O. Ozier, N. S. Baliga, J. T. Wang, D. Ramage, N. Amin, B. Schwikowski, T. Ideker, Cytoscape: A software environment for integrated models of biomolecular interaction networks. Genome Res. 13, 2498–2504 (2003). 14597658 10.1101/gr.1239303 PMC403769

[67] Y. Assenov, F. Ramírez, S.-E. Schelhorn, T. Lengauer, M. Albrecht, Computing topological parameters of biological networks. Bioinformatics 24, 282–284 (2008). 18006545 10.1093/bioinformatics/btm554

[68] T. Stiernagle, Maintenance of C. elegans. WormBook 11, 1–11 (2006). 10.1895/wormbook.1.101.1 PMC4781397 18050451

[69] C. B. Kimmel, W. W. Ballard, S. R. Kimmel, B. Ullmann, T. F. Schilling, Stages of embryonic development of the zebrafish. Dev. Dyn. 203, 253–310 (1995). 8589427 10.1002/aja.1002030302

[70] J.-F. Rual, J. Ceron, J. Koreth, T. Hao, A.-S. Nicot, T. Hirozane-Kishikawa, J. Vandenhaute, S. H. Orkin, D. E. Hill, S. van den Heuvel, M. Vidal, Toward improving *Caenorhabditis elegans* phenome mapping with an ORFeome-based RNAi library. Genome Res. 14, 2162–2168 (2004). 15489339 10.1101/gr.2505604 PMC528933

[71] R. S. Kamath, A. G. Fraser, Y. Dong, G. Poulin, R. Durbin, M. Gotta, A. Kanapin, N. le Bot, S. Moreno, M. Sohrmann, D. P. Welchman, P. Zipperlen, J. Ahringer, Systematic functional analysis of the *Caenorhabditis elegans* genome using RNAi. Nature 421, 231–237 (2003). 12529635 10.1038/nature01278

[72] R. S. Kamath, A. G. Fraser, Y. Dong, G. Poulin, R. Durbin, M. Gotta, A. Kanapin, N. L. Bot, S. Moreno, M. Sohrmann, D. P. Welchman, P. Zipperlen, J. Ahringer, Kidney organoids recapitulate human basement membrane assembly in health and disease. eLife 11, e73486 (2022). 35076391 10.7554/eLife.73486 PMC8849328

[73] A. Edelstein, N. Amodaj, K. Hoover, R. Vale, N. Stuurman, Computer control of microscopes using μManager. Curr. Protoc. Mol. Biol. 14, Unit14.20 (2010). 10.1002/0471142727.mb1420s92 PMC3065365 20890901

[74] J. Schindelin, I. A.-Carreras, E. Frise, V. Kaynig, M. Longair, T. Pietzsch, S. Preibisch, C. Rueden, S. Saalfeld, B. Schmid, Fiji: An open-source platform for biological-image analysis. Nat. Methods 9, 676–682 (2012). 22743772 10.1038/nmeth.2019 PMC3855844

[75] M. J. Randles, S. Collinson, T. Starborg, A. Mironov, M. Krendel, E. Königshausen, L. Sellin, I. S. D. Roberts, K. E. Kadler, J. H. Miner, R. Lennon, Three-dimensional electron microscopy reveals the evolution of glomerular barrier injury. Sci. Rep. 6, 35068 (2016). 27725732 10.1038/srep35068 PMC5057164

[76] E. Wershof, D. Park, D. J. Barry, R. P. Jenkins, A. Rullan, A. Wilkins, K. Schlegelmilch, I. Roxanis, K. I. Anderson, P. A. Bates, E. Sahai, A FIJI macro for quantifying pattern in extracellular matrix. Life Sci. Alliance. **4**, e202000880 (2021). 33504622 10.26508/lsa.202000880 PMC7898596

[77] M. H. Guo, L. Plummer, Y.-M. Chan, J. N. Hirschhorn, M. F. Lippincott, Burden testing of rare variants identified through exome sequencing via publicly available control data. Am. J. Hum. Genet. 103, 522–534 (2018). 30269813 10.1016/j.ajhg.2018.08.016 PMC6174288

[78] D. Smedley, J. O. B. Jacobsen, M. Jäger, S. Köhler, M. Holtgrewe, M. Schubach, E. Siragusa, T. Zemojtel, O. J. Buske, N. L. Washington, W. P. Bone, M. A. Haendel, P. N. Robinson, Next-generation diagnostics and disease-gene discovery with the Exomiser. Nat. Protoc. 10, 2004–2015 (2015). 26562621 10.1038/nprot.2015.124 PMC5467691

[79] L. A. Kelley, S. Mezulis, C. M. Yates, M. N. Wass, M. J. E. Sternberg, The Phyre2 web portal for protein modeling, prediction and analysis. Nat. Protoc. 10, 845–858 (2015). 25950237 10.1038/nprot.2015.053 PMC5298202

[80] E. A. Malone, J. H. Thomas, A screen for nonconditional dauer-constitutive mutations in Caenorhabditis elegans. Genetics 136, 879–886 (1994). 8005442 10.1093/genetics/136.3.879 PMC1205893

