# Supplementary material for: A basement membrane discovery pipeline uncovers network complexity, regulators, and human disease associations
Source: Sci Adv. 2022 May 18;8(20):eabn2265. doi: 10.1126/sciadv.abn2265 (PMC9116610; doi:10.1126/sciadv.abn2265)
Supplement: Supplementary file 1 — Supplementary Text Genomics England Research Consortium Index for tables S1 to S20 Figs. S1 to S11 References [file sciadv.abn2265_sm.pdf]

Science Advances
AAAS

Supplementary Materials for

A basement membrane discovery pipeline uncovers network complexity, new regulators, and human disease associations

Ranjay Jayadev, Mychel R. P. T. Morais, Jamie M. Ellingford, Sandhya Srinivasan, Richard W. Naylor, Craig Lawless, Anna S. Li, Jack F. Ingham, Eric Hastie, Qiuyi Chi, Maryline Fresquet, Nikki-Maria Koudis, Huw B. Thomas, Raymond T. O'Keefe, Emily Williams, Antony Adamson, Helen M. Stuart, Siddharth Banka, Damian Smedley, Genomics England Research Consortium David R. Sherwood*, Rachel Lennon*

*Corresponding author. Email: rachel.lennon@manchester.ac.uk (R.L.); david.sherwood@duke.edu (D.R.S.)

Published 18 May 2022, *Sci. Adv.* **8**, eabn2265 (2022)
DOI: 10.1126/sciadv.abn2265

The PDF file includes:

Supplementary Text
Genomics England Research Consortium
Index for tables S1 to S20
Figs. S1 to S11
References

Other Supplementary Material for this manuscript includes the following:

Tables S1 to S20

Supplementary Text

Generation of BM Zone Network Extended:

Identification of basement membrane and cell surface interactor genes

To build a comprehensive network of basement membrane (BM) zone genes encoding BM proteins and cell surface interactors (CSI), we adopted the following sequential strategy: (i) initial identification of BM zone candidates through gene ontology; (ii) expansion of the BM gene zone network using five independent approaches; and (iii) verification of BM zone localization for candidates (detailed below and illustrated in **Fig. 1A**).

a) Gene ontology retrieval

Gene Ontology (GO) Resource (release 2020-07) (*22*) was used to search for human genes catalogued under the 'Basement membrane' cellular component term (#GO:0008003/GO:0005605). A list of 103 human genes was obtained and used as the initial set of candidate BM zone genes for further analyses in this study. Within the 103 genes, 84 were present in the human matrisome gene list, including 64 structural genes, 17 matrix-associated components, and 3 BM receptors.

b) Network expansion strategy

To expand the GO BM zone list, we established a curating strategy combining five approaches to capture other BM zone candidates not yet included in the human GO list, and strategies to predict potential new BM zone proteins. The approaches included:

(1) *Data curation*: We performed a systematic review of the literature and protein databases (listed in **Table S1**) to search for other putative BM components and cell surface interactors in vertebrates (human, rodents, zebrafish) and invertebrates (*C. elegans*, *Drosophila*) not included in the initial set of BM zone candidates. We used GO resource to screen for additional BM genes in mouse, and then identified and added the respective human orthologs. FASTA sequences for proteins encoded by candidate genes were obtained from UniProtKB and verified for (a) prediction of a signal peptide with PredSi (http://www.predisi.de/index.html); (b) transmembrane domains with the online tools TMHMM server v. 2.0 (http://www.cbs.dtu.dk/services/TMHMM/) and Phobius (https://phobius.sbc.su.se); and (c) evidence of secretion to the extracellular space and/or shedding from the cell surface for transmembrane proteins.

(2) *BM gene expression signature in C. elegans*: Most BM protein encoding genes in *C. elegans* are synthesized in distant tissues, secreted into the extracellular fluid, and recruited to BMs for incorporation. In particular, we noticed that 15 of the 18 known BM components in the worm (all BM genes listed previously (*21*) except for *agr-1*, *pxn-2*, and *lam-1*) are synthesized in either the body wall muscles, or the body wall muscles and the epidermis (data derived from

https://wormbase.org). We hypothesized that genes within the *C. elegans* matrisome (*14*), that possess this expression signature, could be BM gene candidates. Of 467 conserved matrisome genes, we identified 81 with the BM gene expression signature (**Table S2**).

(3) *Conservation and enrichment analysis of BM protein domains*: We obtained a list of annotated putative BM protein domains for the genes in the expanding BM zone list from Interpro (https://www.ebi.ac.uk/interpro/; see **Table S3**). Using R software (R Core Team, 2020, https://www.R-project.org/), and the enricher function of the ClusterProfiler package (v3.14.3), we performed a domain enrichment analysis, taking the domain annotations for the entire human proteome as background. We identified 20 domains that were significantly over-represented in the expanding BM zone genes (adjusted p-value < 0.05, **Table S3**) and applied these domains as a filter onto the whole human proteome. We identified 25 additional BM zone gene candidates based on BM domain conservation (**Table S4**). Moreover, to determine whether human protein domains enriched in our BM zone list are conserved, we performed similar analyses for mouse, zebrafish, *Drosophila* and *C. elegans* ortholog genes.

(4) *Nearest interacting neighbors of BM proteins*: To identify new BM zone gene candidates based on protein interaction, we searched for interacting partners of the proteins in the expanding BM zone list using the STRING (https://string-db.org/, version 11.0) database (*24*) at a combined score threshold $\geq$ 70% (indicating high confidence interactions). This analysis returned 1533 BM interacting partners that were further filtered for presence of a signal peptide, enriched BM protein domains found in the previous analysis, and for direct interactions with at least 20 other BM proteins (**Table S4**). False positives (e.g., COL1A1, COL1A2, COL3A1) were manually removed and this analysis added 16 novel BM zone candidate genes.

(5) *BM zone localization in C. elegans*: 36 genes have previously been localized to the BM zone in *C. elegans* (**Table S5**, see genes tagged in previous studies), and human orthologs of these genes that were not already curated from the above analyses were added as candidates.

Together, these network expansion approaches identified 160 additional genes, resulting in a combined BM zone gene network comprising 263 candidates (**Table S6**).

c) Verification of BM zone localization

To verify BM zone localization for the 263 genes in the expanded network, we first reviewed the literature and protein databases for evidence of tissue protein immunolocalization to BM zone in human, rodents, zebrafish, *Drosophila,* and *C. elegans*. We used the article search tools NCBI PubMed (https://pubmed.ncbi.nlm.nih.gov/) and Google Scholar (https://scholar.google.com/) to find publications documenting BM zone localization. We also visually assessed immunohistochemical images of human tissues in the Human Protein Atlas (https://www.proteinatlas.org) and mouse tissues the Matrixome Project database (*20*) (http://dbarchive.biosciencedbc.jp/archive/matrixome/bm/home.html) to look for BM zone immunolocalization of candidates in the expanded network. Next, we fluorescently tagged a subset of *C. elegans* orthologs to investigate their localization, focusing on small gene families with clear orthology to human genes where none or only some of the family members have been localized to the BM zone in previous studies. We generated endogenous mNeonGreen or

mRuby2 tags for 17 BM and 8 CSI candidate genes (see **Materials and Methods** and **Fig. S2**) and localized 14 of these genes to the BM zone (**Table S5**). Together, our verification strategies confirmed BM zone localization of 184 genes in the network (see **Fig. S1A**).

Among the remaining candidates without direct BM zone immunolocalization evidence, 38 were predicted to be in BM zone based on two metrics: high confidence interaction with one or more BM zone candidates (interaction data obtained from STRING at a combined score threshold ≥ 70%); and/or evidence for proteolytic activity on BM substrates (data collected from BRENDA, https://www.brenda-enzymes.org/; MEROPS, https://www.ebi.ac.uk/merops/; and publications; see **Fig. S1A**).

We could not confirm or predict BM zone localization for 41 candidates due to insufficient evidence and/or GO mis-annotations (e.g., ALB, ANXA2, see **Fig. 1A**). Thus, the integrated verified BM zone network comprised 163 BM components and 62 CSI. To validate this finalized BM zone list, we repeated the analysis for conservation and enrichment of BM protein domains (as described earlier). The validated list (**Table S7**) was used for subsequent analyses in this study. However, three components that were in later analyses were modified during the final proofing of the BM zone gene network prior to publication—PHF13 was a mis-annotation and was removed from the network; and HAPLN2 and MEGF6, which were erroneously classified under confirmed localization evidence, were transferred to the insufficient evidence category.

We adopted a colour code to distinguish components with confirmed (green) or predicted (magenta) localization to BM zone (**Fig. 1A**). Components in the insufficient evidence category were coloured in grey. Finally, to curate this network, we created an open access, interactive resource: ***b****asement* ***m****embrane*BASE (https://bmbase.manchester.ac.uk/).

d) Identification of vertebrate and invertebrate orthologs

Orthologs for the 222 human BM zone genes were identified in mouse (*Mus musculus*) using MouseMine (http://www.mousemine.org/mousemine/begin.do), zebrafish (*Danio rerio*) using ZFIN (http://zfin.org/), fruit fly (*Drosophila melanogaster*) using FlyBase (flybase.org), and worm (*Caenorhabditis elegans*) using OrthoList 2 (http://ortholist.shaye-lab.org/) and WormBase (wormbase.org/). We also used Ensembl (http://ensembl.org/index.html), NCBI (https://www.ncbi.nlm.nih.gov), and DIOPT (https://www.flyrnai.org/cgi-bin/DRSC_orthologs.pl) databases for verification. All orthologs are listed in **Table S7**.

Clinical histories

LAMA5: *LAMA5* compound heterozygous variants c.3282+5G>A, and c.9489C>T;p.Tyr3163* were identified in two early fetal losses. Chromosome array comparative genomic hybridization was normal for both fetuses, 7-dehydrocholesterol was normal in the first fetus. The first fetus was noted to have spina bifida, hypoplastic cerebellum, absent corpus callosum, ventricular septal defect, enlarged multicystic solitary left kidney, absent right kidney and urinary bladder, absent thymus, bilateral cleft lip and palate, laryngeal atresia, small ears with absent canals, cutaneous syndactyly 3-4th fingers and 2-3rd toes, exomphalos, imperforate anus and bilateral

talipes. The second fetus was noted to have spina bifida, ventricular septal defect, bilateral kidney agenesis, absent thymus, laryngeal stenosis, cutaneous syndactyly 2-3rd and partial syndactyly 3-4th fingers, syndactyly 2-3-4th toes and anteriorly placed anus. There was negative panel screening for CAKUT v1.4 and specifically Fraser syndrome, and also negative screening for arthrogryposis v3, clefting v2.0 disorder of sex development v2.1, hydrocephalus v2.1, intellectual disability v3.0, limb disorder v2.0 malformations of cortical development v2.2, non-syndromic familial congenital anorectal malformations v1.6, and VACTERL-like phenotype v1.25. Both pregnancies were terminated due to fetal malformations.

MPZL2: The individual with the homozygous c.72del variant was the father of the individual recruited to the 100KGP. The father was diagnosed with cystic kidney disease at the age of 35. The daughter (c.72del heterozyote) had multiple kidney cysts diagnosed at the age of 15 and no other phenotypes described. It was not possible to obtain further clinical information from the recruiting clinician.

MATN2: Individual 1 (C.1081+3_1081+6del): male proband with early onset or familial intestinal pseudo-obstruction at the age of >1 year; Individual 2 (c.1585del): male proband recruited at the age of >1 year into Ultra-rare disorder Disease Group (from the Genomics England 100,000 genomes project); Individual 3 (c.746G>C and C.1450+1G>A case 3): male proband diagnosed with intellectual disability at the age of 6. It was not possible to obtain further clinical information from the recruiting clinicians.

Genomics England Research Consortium

The Genomics England Research Consortium

Ambrose, J. C. [1]; Arumugam, P.[1]; Bevers, R.[1]; Bleda, M. [1]; Boardman-Pretty, F. [1,2]; Boustred, C. R. [1]; Brittain, H.[1]; Brown, M.A.; Caulfield, M. J.[1,2]; Chan, G. C. [1]; Fowler, T. [1]; Giess A. [1]; Hamblin, A.[1]; Henderson, S.[1,2]; Hubbard, T. J. P. [1]; Jackson, R. [1]; Jones, L. J. [1,2]; Kasperaviciute, D. [1,2]; Kayikci, M. [1]; Kousathanas, A. [1]; Lahnstein, L. [1]; Leigh, S. E. A. [1]; Leong, I. U. S. [1]; Lopez, F. J. [1]; Maleady-Crowe, F. [1]; McEntagart, M.[1]; Minneci F. [1]; Moutsianas, L. [1,2]; Mueller, M. [1,2]; Murugaesu, N. [1]; Need, A. C. [1,2]; O'Donovan P. [1]; Odhams, C. A. [1]; Patch, C. [1,2]; Perez-Gil, D. [1]; Pereira, M. B.[1]; Pullinger, J. [1]; Rahim, T. [1]; Rendon, A. [1]; Rogers, T. [1]; Savage, K. [1]; Sawant, K. [1]; Scott, R. H. [1]; Siddiq, A. [1]; Sieghart, A. [1]; Smith, S. C. [1]; Sosinsky, A. [1,2]; Stuckey, A. [1]; Tanguy M. [1]; Taylor Tavares, A. L.[1]; Thomas, E. R. A. [1,2]; Thompson, S. R. [1]; Tucci, A. [1,2]; Welland, M. J. [1]; Williams, E. [1]; Witkowska, K. [1,2]; Wood, S. M. [1,2].

1. Genomics England, London, UK

2. William Harvey Research Institute, Queen Mary University of London, London, EC1M 6BQ, UK.

3rd February 2022

Table Index

Table S1. Protein databases used for the expansion of the BM zone network and verification of candidate BM localization.

Table S2. Conserved *C. elegans* matrisome genes with the BM gene expression signature.

Table S3. Interpro protein domains enriched in BM zone protein candidates.

Table S4. Candidate BM zone proteins identified by BM protein domain enrichment analysis and from predicted interaction with BM proteins.

Table S5. BM zone localization data for *C. elegans* orthologs of BM and CSI candidates fluorescently tagged in this and previous studies.

Table S6. Expanded list of BM zone gene network candidates.

Table S7. Verified BM zone gene network.

Table S8. BM zone protein abundance derived from human proteomic datasets.

Table S9. Human and mouse gene expression datasets used in this study.

Table S10. Expression variance of BM zone genes in human and mouse transcriptomic datasets.

Table S11. Connectivity metrics for human and *C. elegans* BM domain-based networks.

Table S12. Post-embryonic RNAi screen of 77 *C. elegans* BM zone gene orthologs.

Table S13. Human disease phenotype associations for BM zone genes within OMIM and PanelApp databases.

Table S14. Top-level HPO terms associated with BM zone genes.

Table S15. gnomAD constraint metrics for tolerance to pLoF variation within BM zone genes.

Table S16. BM zone gene variants identified in the 100KGP rare disease cohort.

Table S17. BM zone genes with homozygous pLoF variants in 100KGP.

Table S18. Proteomic analysis of core BM protein levels in podocyte-derived matrix upon *MATN2* knockdown.

Table S19. *C. elegans* and zebrafish strains used in this study.

Table S20. gRNA sequences used for CRISPR-Cas9-mediated knockdown in zebrafish.

Fig. S1

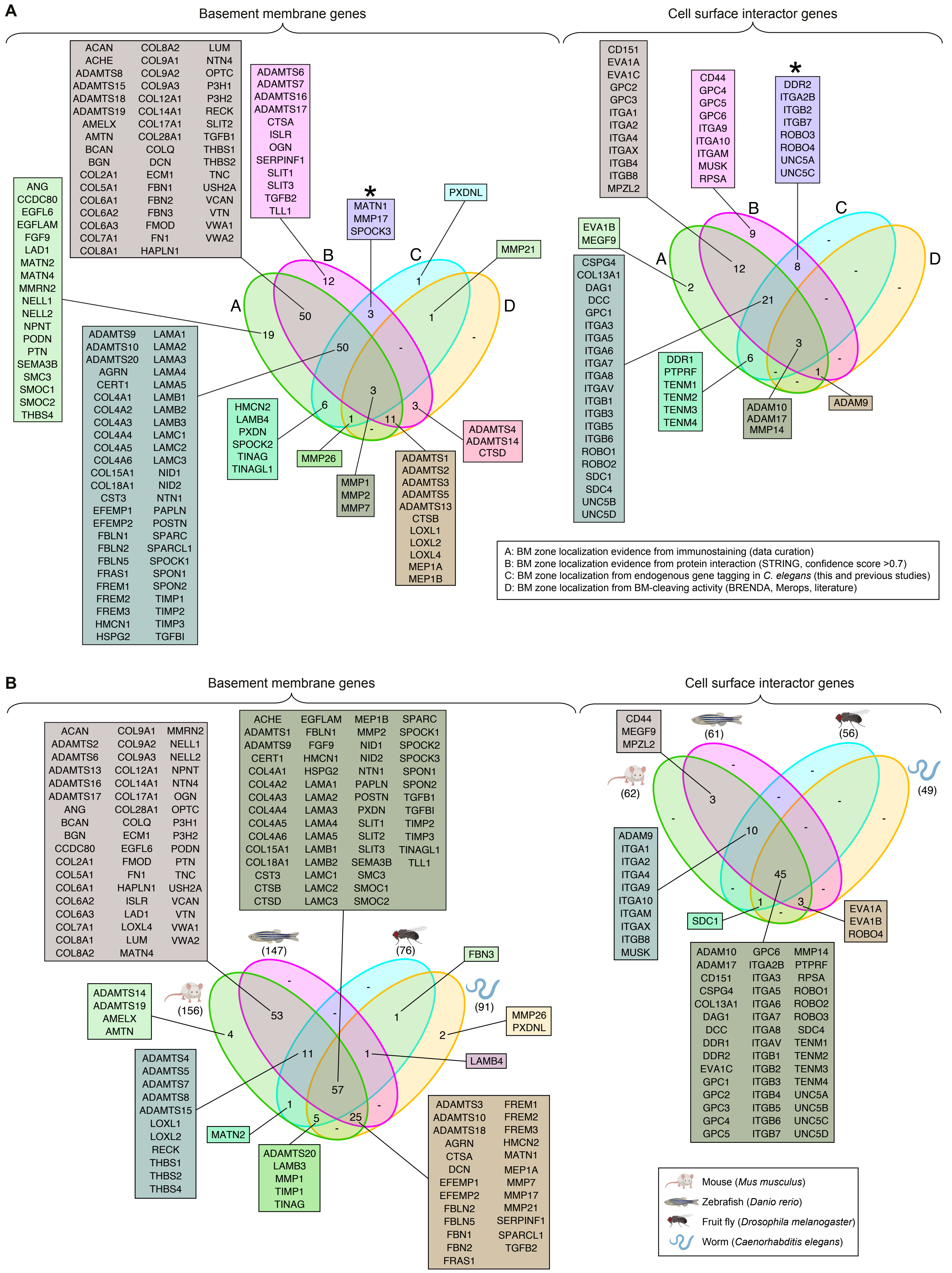

Fig. S1. Verification strategies for basement membrane zone localization and conservation of network genes. (**A**) Venn diagrams illustrate how the localization of human proteins in the integrated basement membrane zone network was verified. Asterisks indicate proteins with predicted localization whose *C. elegans* orthologs were detected in the BM zone through fluorescent tagging. (**B**) Human basement membrane and cell surface interactor genes are shown in boxes and the presence of corresponding orthologs in mouse, zebrafish, *D. melanogaster*, and *C. elegans* is represented in Venn diagrams. See also **Fig. S4** and **Table S7**. Animal illustrations made with https://biorender.com.

Fig. S2

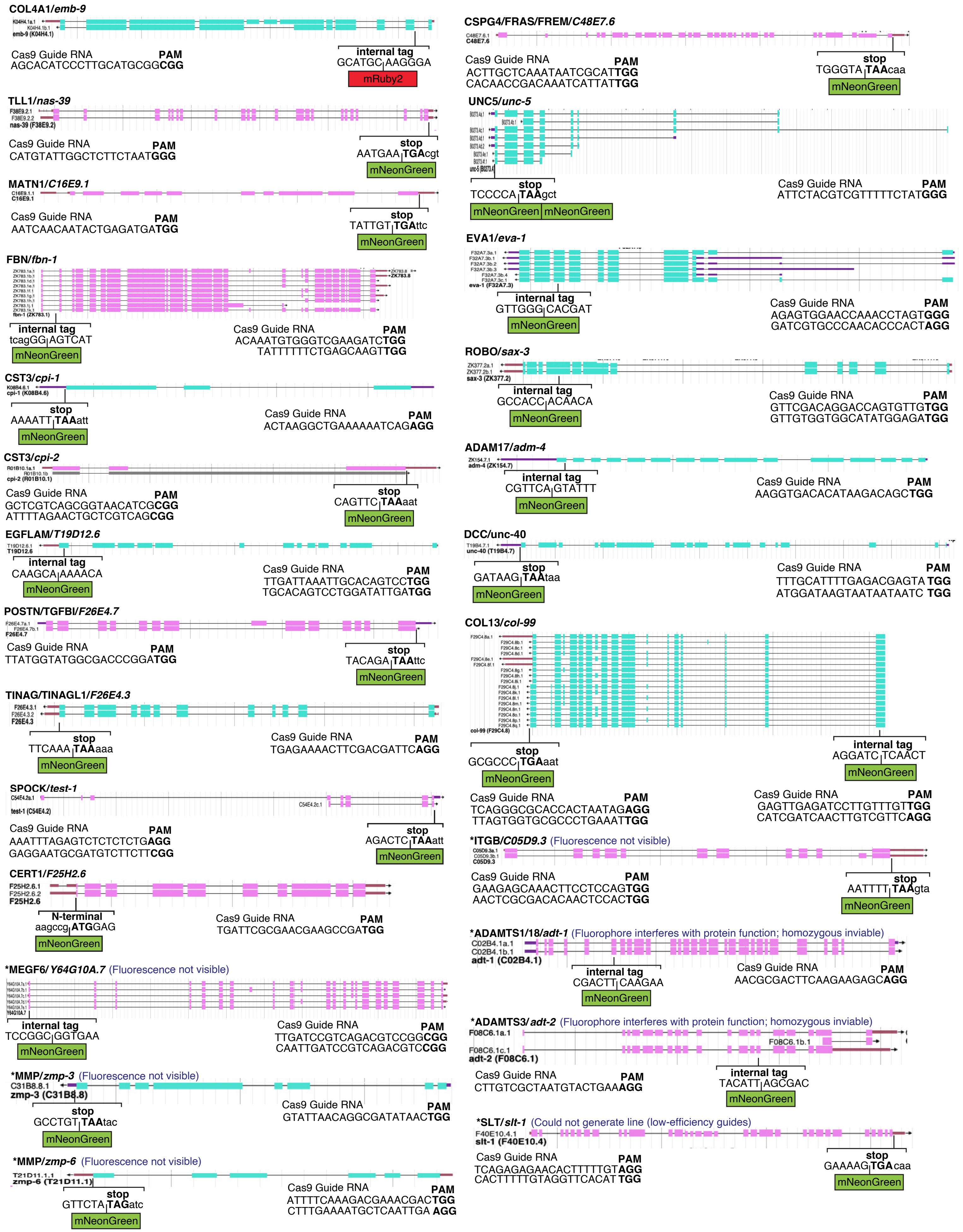

Fig. S2. Fluorescent protein insertions at endogenous gene loci of basement membrane zone candidates in *C. elegans*.
Worm gene schematics (adapted from https://wormbase.org) indicate genomic locations where mNeonGreen (green box), two tandem mNeonGreen (two green boxes), or mRuby2 (red box) fluorophores were inserted for BM and CSI candidates tagged in this study. For each gene, sgRNA, PAM site, and insertion site sequences are provided. Asterisks indicate loci for which challenges were encountered during tagging attempts. Every gene was tagged at a single locus, except for *col-99*, which was tagged at two separate loci. Note that exons are colored magenta or cyan depending on alignment to the forward or reverse strand of the reference genome, respectively.

Fig. S3

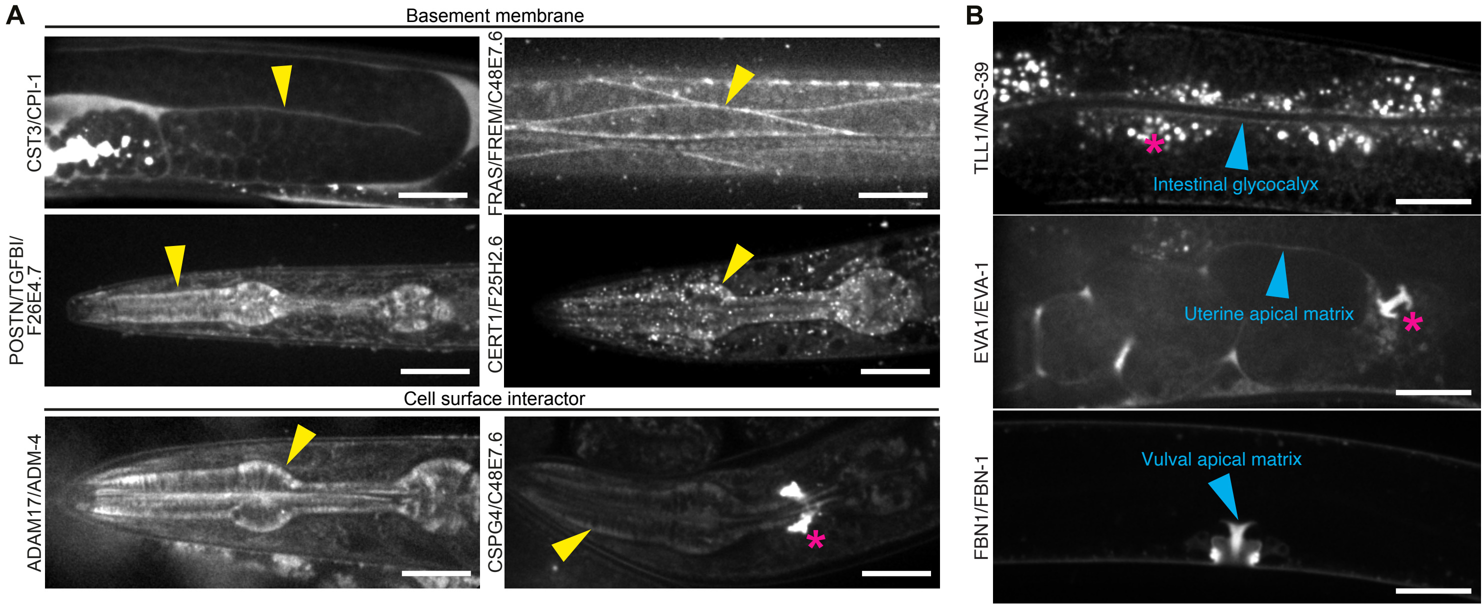

Fig. S3. Visualization of basement membrane zone candidate localization in *C. elegans*.
(**A**) Confocal middle-plane z-slices of tagged candidates in adult animals showing BM zone localization (yellow arrowheads) to either the pharyngeal or gonadal BM, except for FRAS/FREM/C48E7.6, which localizes to the body wall muscle BM. (**B**) Confocal z-slices of candidates not detected in the BM zone but present in other matrices (blue arrowheads). Asterisks in panels **A** and **B** indicate fluorescence signal in the nerve ring for C48E7.6, spermathecal-uterine valve for EVA-1, and autofluorescence within gut granules for NAS-39. Scale bar represents 25 µm.

Fig. S4

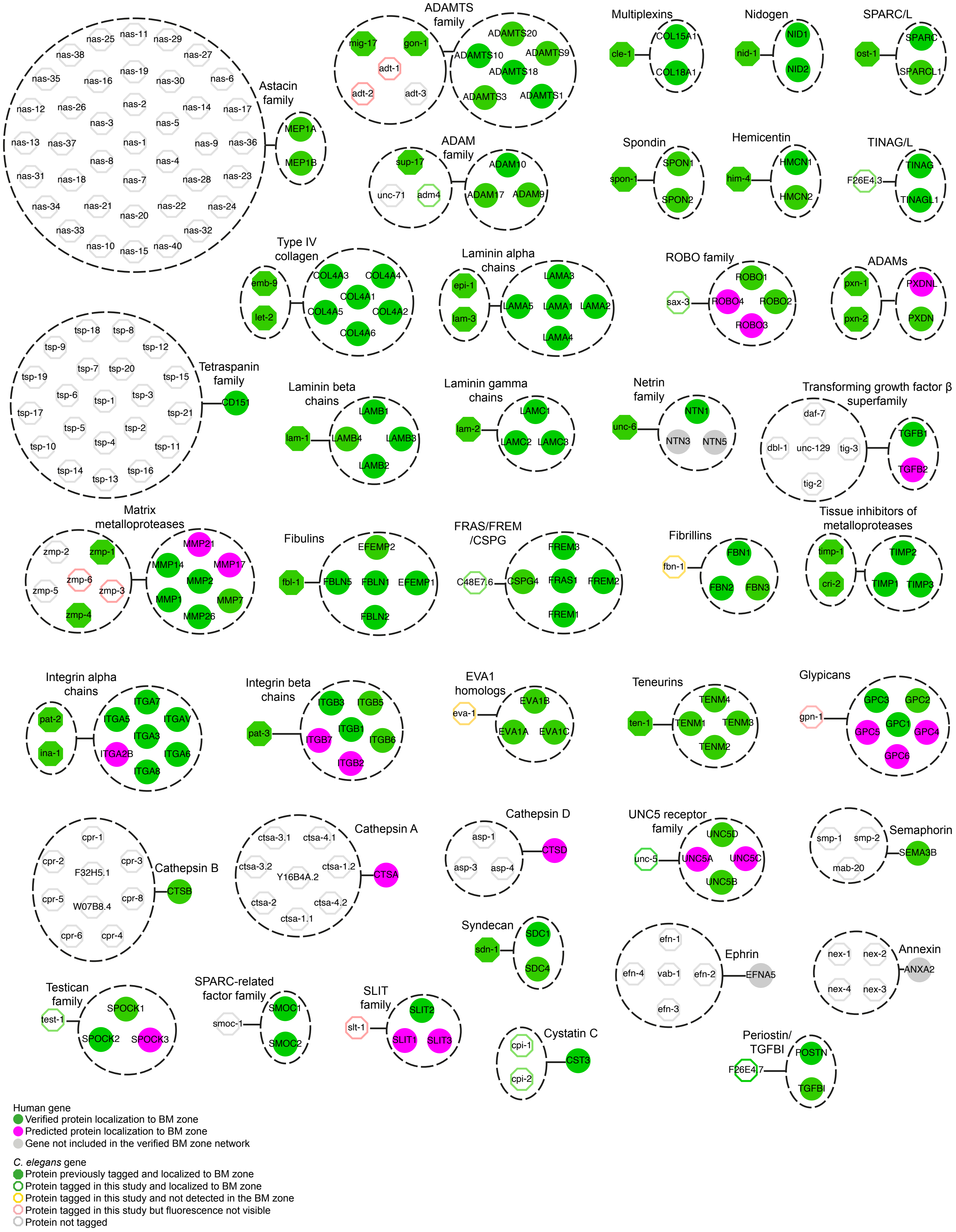

Fig. S4. Basement membrane zone gene families in *C. elegans*.
Diagrams illustrate the orthologous relationships of *C. elegans* genes and gene families (on the left) with human BM zone genes (on the right). Note the various one-to-many and many-to-many relationships between several genes for both species.

Fig. S5

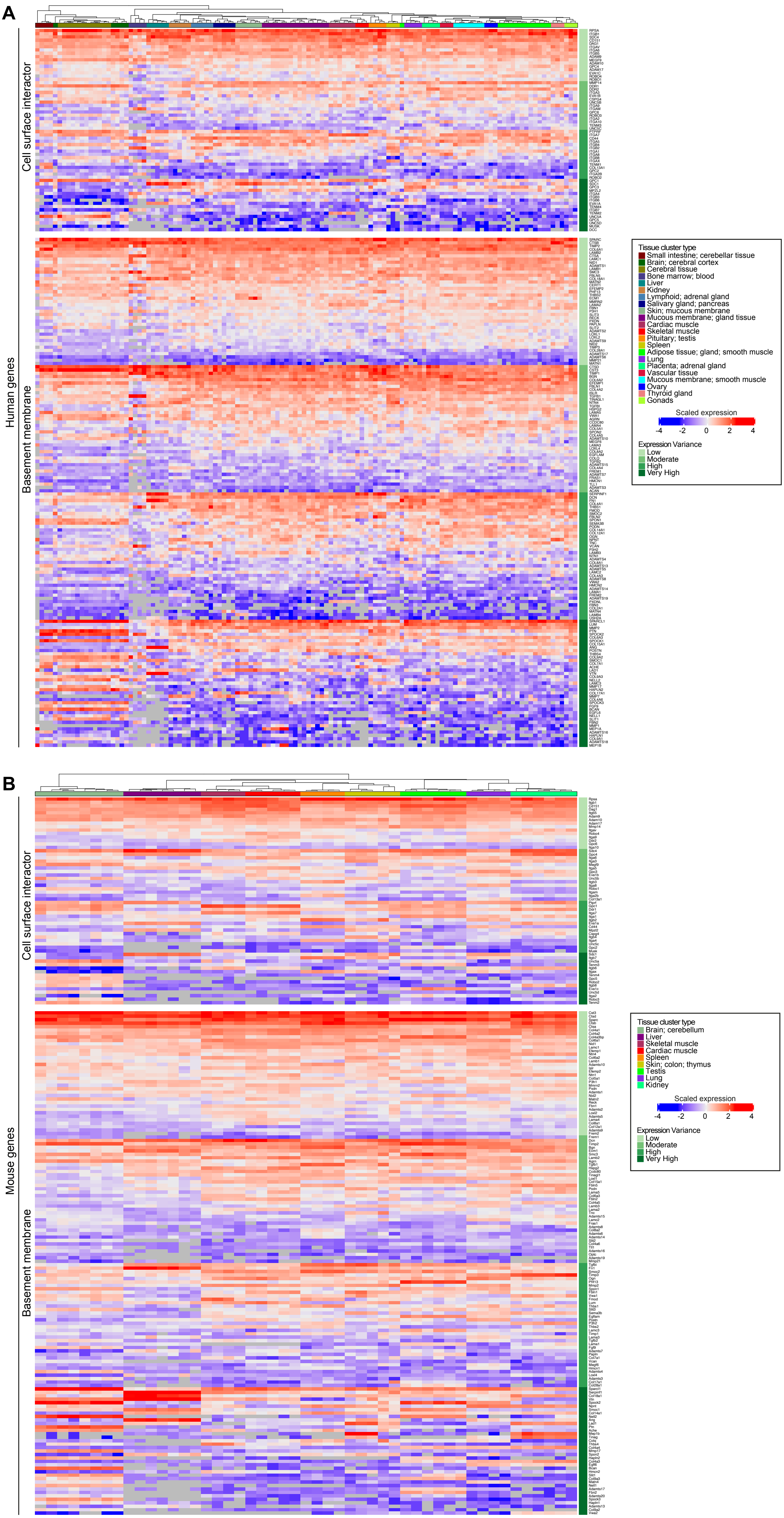

Fig. S5. Expression variance of mammalian basement membrane zone genes. Heatmaps indicate scaled gene expression of BM matrix and CSI genes across different (**A**) human and (**B**) mouse tissues (transcriptomic datasets used in this study detailed in **Table S9**). Genes are binned according to expression variance (low, moderate, high, very high; **Table S10**). Full-sized heatmaps available on figshare: https://doi.org/10.6084/m9.figshare.c.5662348.

Fig. S6

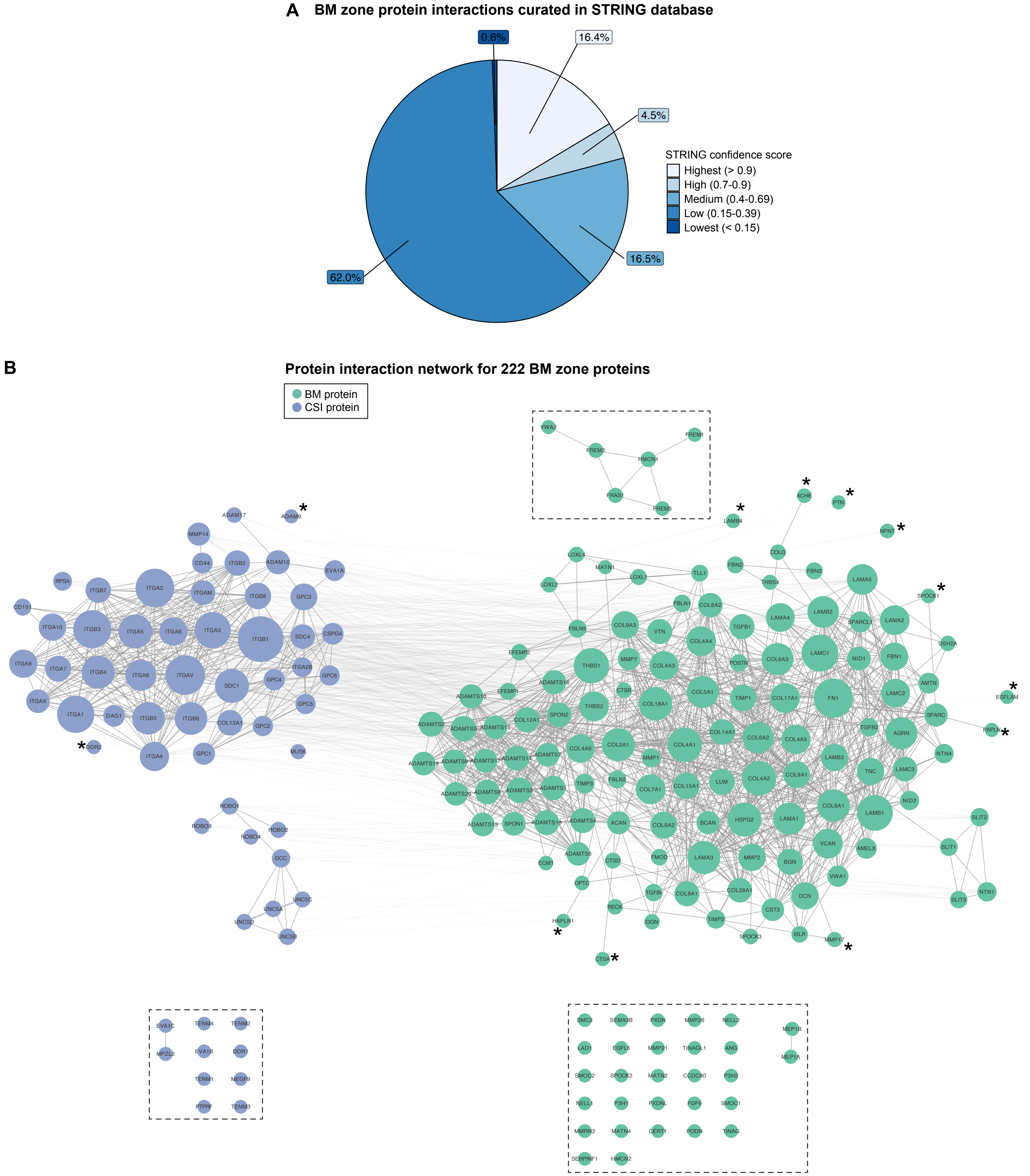

Fig. S6. BM zone protein interaction network.
(**A**) Pie chart illustrating the stratification of BM zone protein interactions according to their interaction confidence scores in the STRING database (supporting data available on figshare: https://doi.org/10.6084/m9.figshare.c.5662348). (**B**) Interactome for human BM zone proteins derived from interaction data curated in the STRING database. Nodes represent BM and CSI proteins and are sized according to degree of interactions with other proteins in the network. Lines connecting nodes represent respective protein interactions. Asterisks indicate examples of proteins that are at the periphery of the network with very few reported interactions. Boxes denote BM zone proteins excluded from the main network due to lack or scarcity of interaction data. (supporting data available on figshare: https://doi.org/10.6084/m9.figshare.c.5662348).

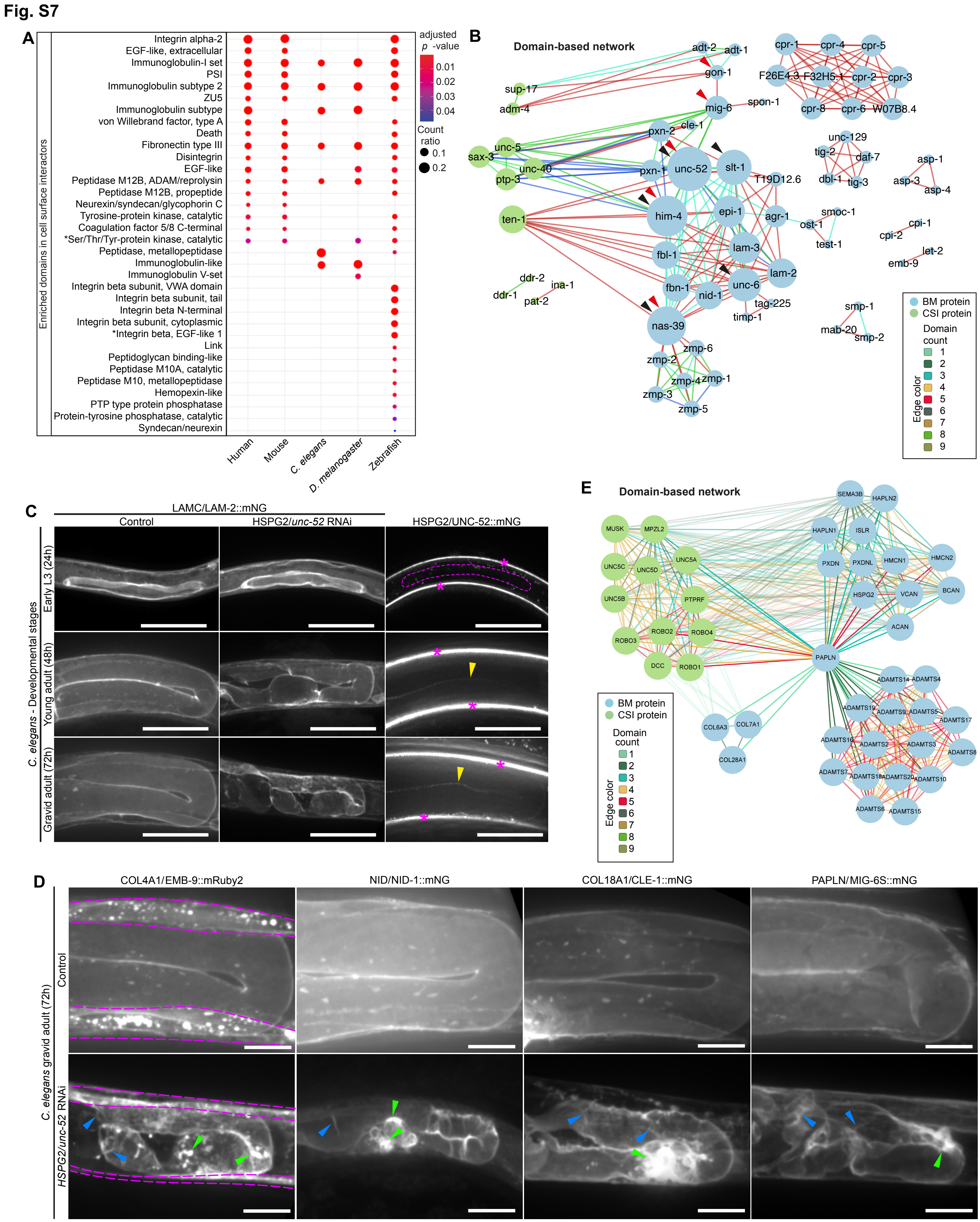

Fig. S7. Perlecan/UNC-52 and papilin/MIG-6 are hub proteins and depletion of *C. elegans* UNC-52 disrupts basement membrane organization.
(**A**) Conservation and enrichment of Interpro protein domains (as described in **Fig. 3A**) in verified CSI components. (**B**) A domain-based interactome (as described in **Fig. 3B**) for *C. elegans* BM zone genes. (**C**) Left, representative confocal sum projections of gonadal BM LAMC/LAM-2::mNG in control and *unc-52* RNAi-treated early L3, young adult, or gravid adult animals (*n* = 10 animals examined each). Additional supporting images available on figshare: https://doi.org/10.6084/m9.figshare.c.5662348. Right, confocal middle-plane z-slices of HSPG2/UNC-52::mNG, which is not detected at the gonadal BM (outlined in magenta) in the early L3 stage, but is visible in the young adult and gravid adult stages (yellow arrowheads, *n* ≥ 13 animals examined each). Asterisks indicate strong signal in the body wall muscle BM and muscle-epidermal attachment sites. Scale bar represents 25 µm. (**D**) Representative confocal sum projections of gonadal BM COL4A1/EMB-9::mRuby2, NID/NID-1::mNG, COL18A1/CLE-1::mNG, and PAPLN/MIG-6S::mNG in control and *unc-52* RNAi-treated 72-h adult animals (*n* = 5 animals examined each). Note the fibrillar structures (blue arrowheads) and aggregates (green arrowheads) of fluorescence within the gonadal BM upon knockdown of *unc-52*. Magenta dashed lines demarcate body wall muscle tissue where EMB-9::mRuby2 is produced. Scale bar represents 25 µm. Additional supporting images available on figshare: https://doi.org/10.6084/m9.figshare.c.5662348. (**E**) Sub-network highlighting diverse groups of human BM zone proteins that share domains with papilin.

Fig. S8

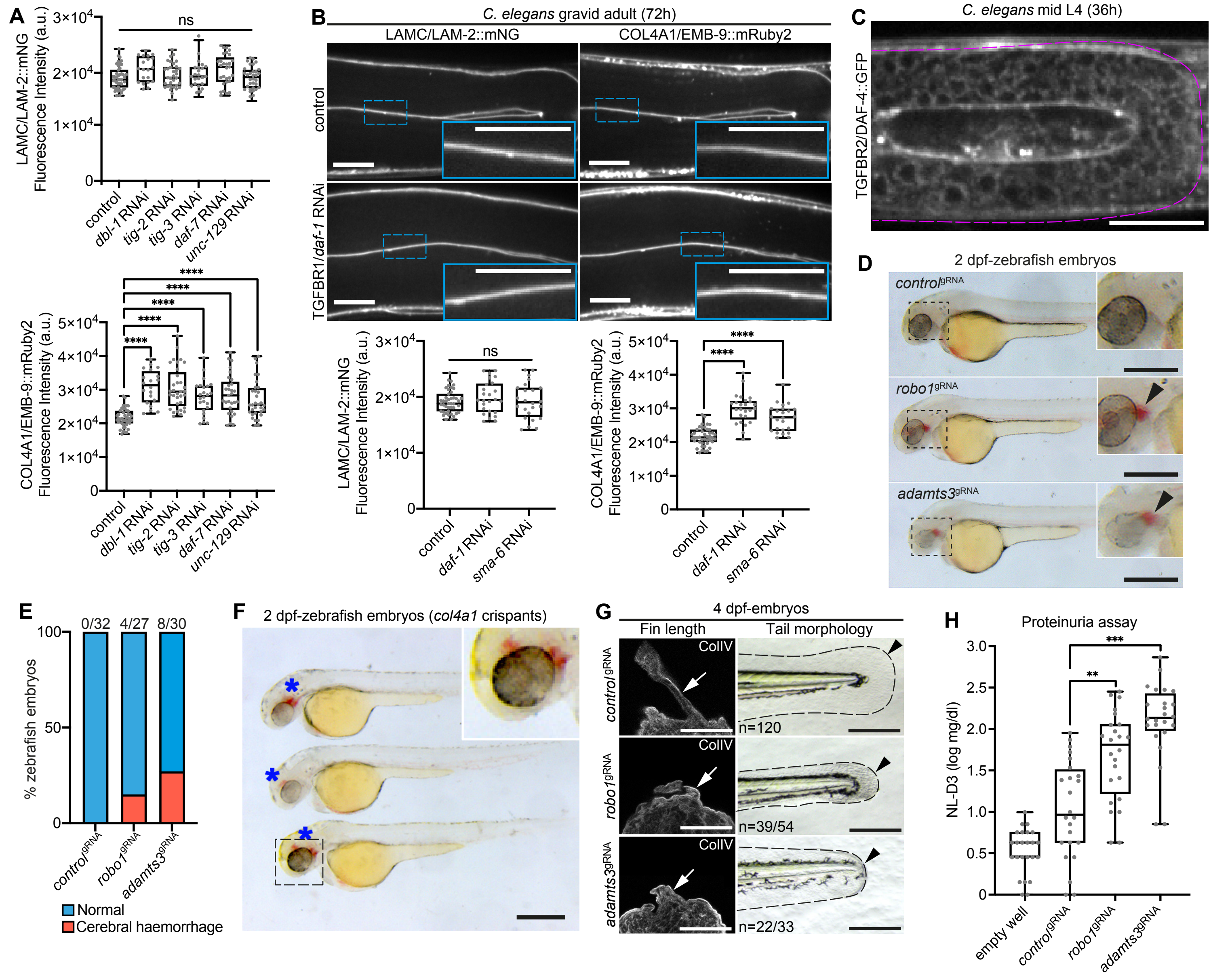

Fig. S8. TGFβ regulates BM collagen IV levels in *C. elegans*, and depletion of ADAMTS3 and ROBO impairs development and kidney BM function in zebrafish. (**A**) Quantification of LAMC/LAM-2::mNG and COL4A1/EMB-9::mRuby2 gonadal BM fluorescence intensity upon knockdown of TGF-β ligand genes in *C. elegans* ($n \geq 20$ each). (**B**) Confocal z-slices of LAMC/LAM-2::mNG and COL4A1/EMB-9::mRuby2 (top) and quantification of fluorescence intensity (bottom) upon knockdown of TGF-β type I receptor genes ($n \geq 20$ each). Note that the sole type II receptor gene *daf-4* could not be examined as loss of DAF-4 results in early larval developmental arrest (*80*). Scale bar represents 25 µm. (**C**) Confocal z-slice of TGFβ type II receptor DAF-4::GFP depicting gonadal BM zone localization (outer tissue boundary in magenta). Scale bar represents 25 µm. (**D**) Brightfield images of 2 dpf-zebrafish embryos injected with indicated gRNAs for *robo1* and *adamts3* (boxed regions magnified in insets, arrowheads indicate intracerebral haemorrhage). Scale bar represents 600 µm. (**E**) Bar chart showing the observed frequency of intracerebral haemorrhage in *robo1* and *adamts3* crispants zebrafish embryos compared to controls. (**F**) Brightfield images of 2-days post fertilization (dpf) *col4a1*^{gRNA}-injected (*col4a1* crispant) zebrafish embryos with intracerebral haemorrhage (asterisks). Scale bar represents 600 µm. (**G**) Confocal images of collagen IV immunofluorescence (left) and brightfield images (right) of tail regions (dashed lines); arrows and arrowheads indicate reduced fin length ($n = 5$ animals examined each) and fin fold extension respectively in *robo1* and *adamts3* crispants. (**H**) Assessment of proteinuria (NL-D3 levels) in *robo1* and *adamts3* crispants ($n = 24$ each). For all boxplots, ****$p < 0.0001$,***$p < 0.001$, **$p < 0.01$, ns—not significant; one-way ANOVA with post-hoc Dunnett's test; edges indicate the 25th and 75th percentiles, the line in the box represents the median, and whiskers mark the minimum and maximum values.

Fig. S9

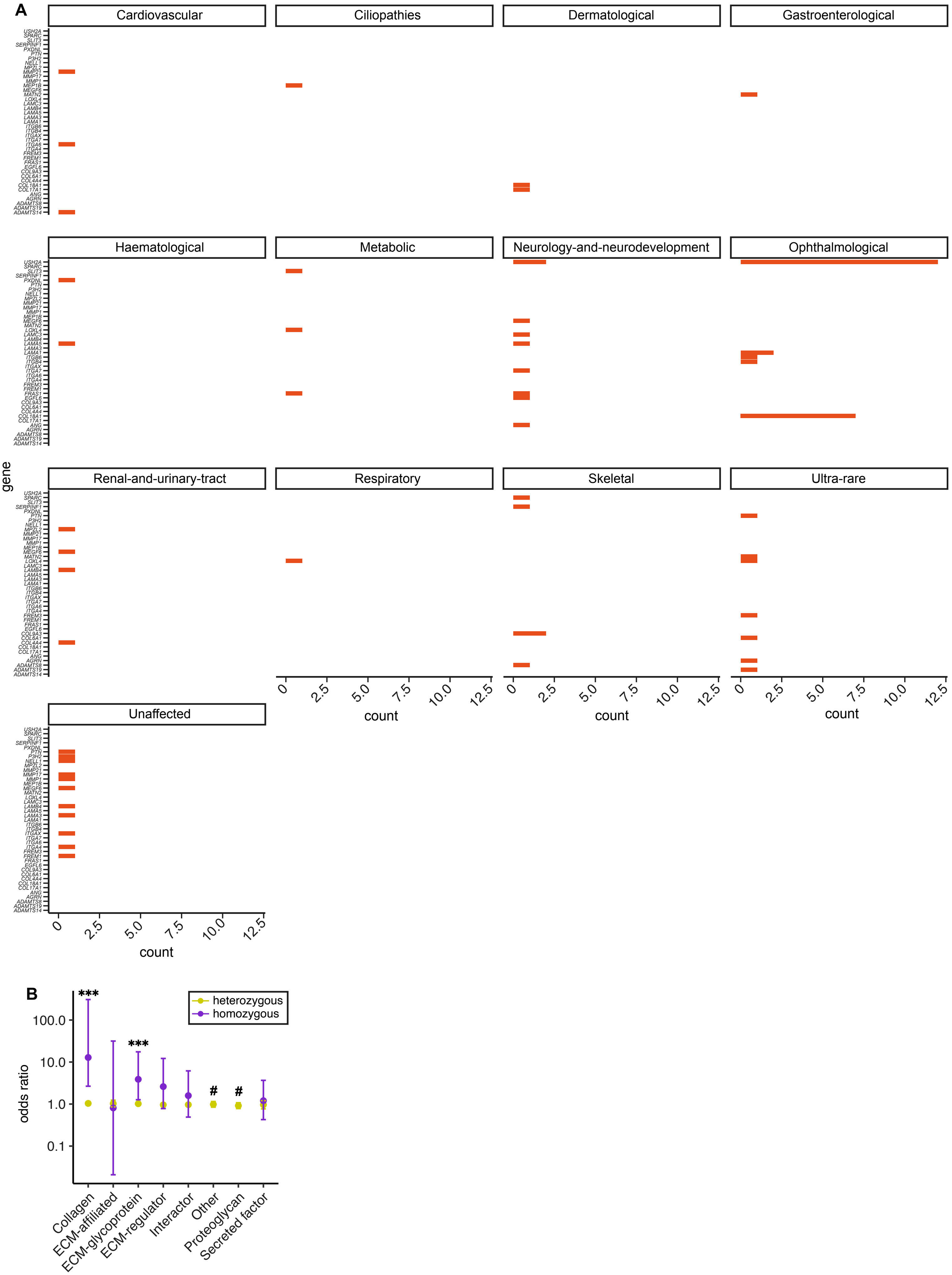

Fig. S9. Landscape of predicted loss-of-function variants in BM zone genes within 100KGP cohort.
(**A**) Classification of homozygous pLoF variants identified in 40 BM zone genes according to type of rare disease presented in associated 100KGP individuals. Count indicates number of variants observed in the respective genes. (**B**) Odds ratio (OR) plots (comparing individuals with disease to unaffected relatives) for heterozygous and homozygous predicted loss-of-function (pLoF) variants in BM zone genes grouped by their matrisome classification. *****p* < 0.0001, Fisher's Exact test. #ORs for these homozygous pLoF variants could not be computed as there were no matched controls in 100KGP.

Fig. S10

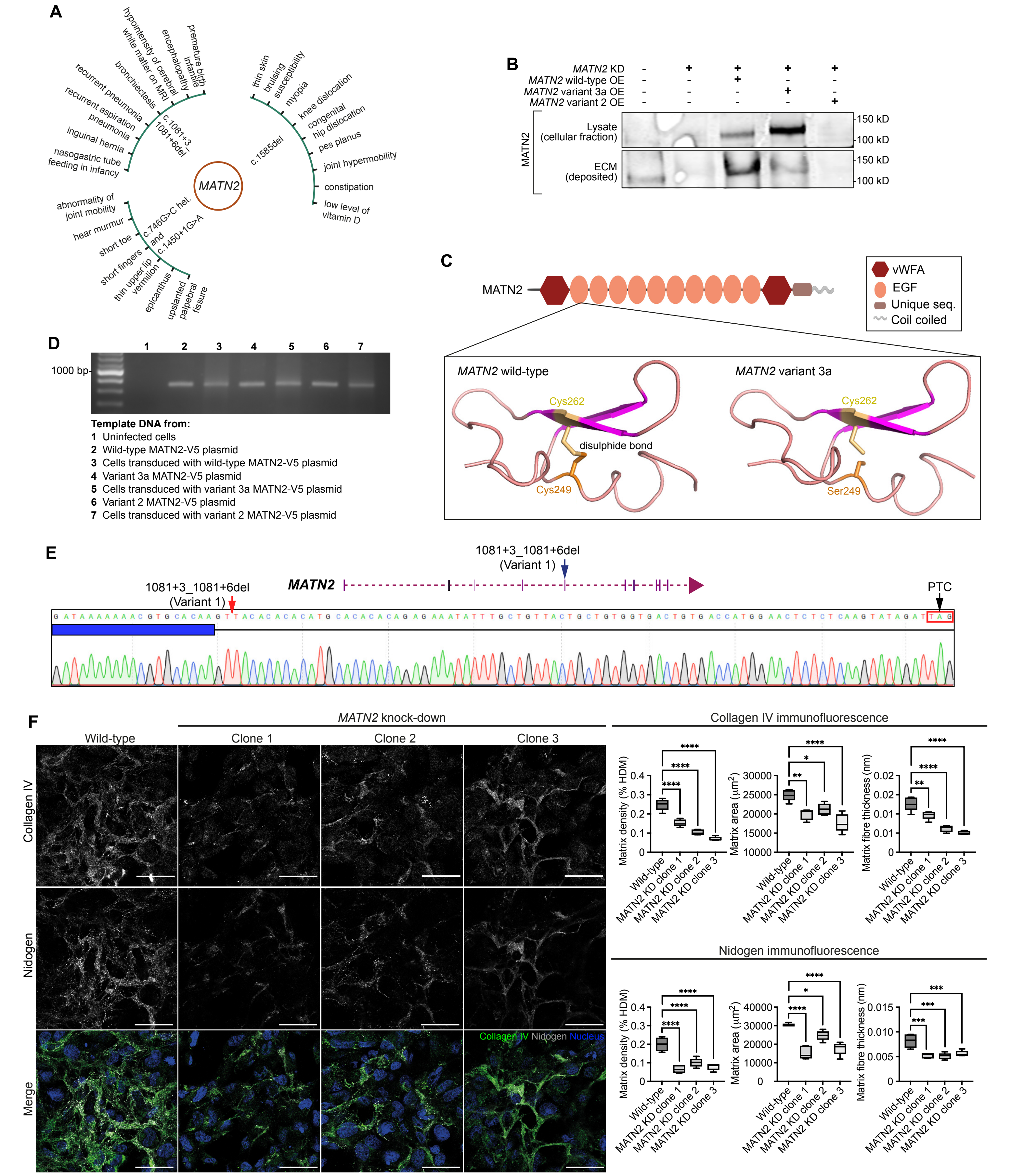

Fig. S10. Functional analyses of *MATN2* variants.
(**A**) Phenotypes observed in 100KGP individuals carrying *MATN2* pLoF variants. (**B**) Western blots of MATN2 in lysate and ECM fractions derived from endogenous MATN2-depleted human podocytes over-expressing V5-tagged wild-type or variant *MATN2*. Immunoblotting source data available on figshare: https://doi.org/10.6084/m9.figshare.c.5662348. (**C**) Top, human MATN2 protein domain structure. Bottom, a 3D model of the MATN2 EGF1 domain indicating disruption of the highlighted disulfide bond in the *MATN2*$^{\text{c.746G>C, p.Cys249Ser}}$ missense variant. (**D**) PCR amplification of the V5 tag using DNA extracted from podocytes transduced with the listed MATN2 constructs. PCR products amplified from the respective construct plasmids are shown as positive controls. No product was amplified from uninfected cells (negative control). (**E**) *In vitro* splicing assay with a minigene for *MATN2* variant 1 indicating aberrant splicing and the introduction of a premature stop codon (PTC). (**F**) Left, type IV collagen and nidogen immunofluorescence in podocyte-derived matrix from wild-type cells and three clonal populations of MATN2-depleted podocytes. Podocyte nuclei were visualized by DAPI staining (shown in blue). Scale bar represents 50 µm. Full z-stacks of immunofluorescence images available on figshare: https://doi.org/10.6084/m9.figshare.c.5662348. Right, quantification of immunofluorescence signal in podocyte-derived matrix using matrix density, area, and fiber thickness metrics (**Materials** and **Methods**). ****$p < 0.0001$, ***$p < 0.001$, **$p < 0.01$, *$p < 0.05$, ns—not significant; one-way ANOVA with post-hoc Dunnett's test. For boxplots, edges indicate the 25th and 75th percentiles, the line in the box represents the median, and whiskers mark the minimum and maximum values.

Fig. S11

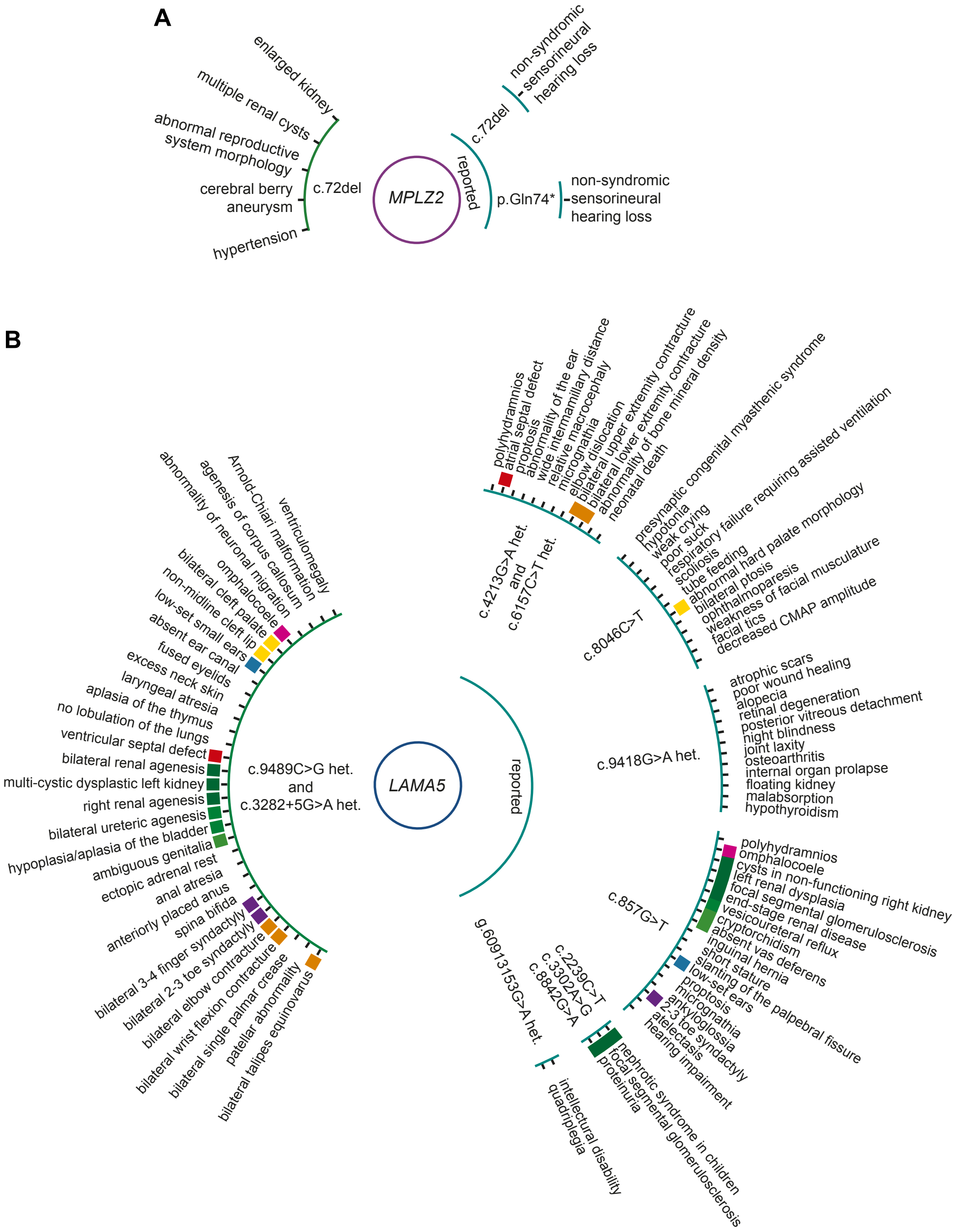

Fig. S11. Disease phenotypes observed in 100KGP individuals carrying (A) *MPZL2* and (B) *LAMA5* pLoF variants. Note that previously reported phenotypes are also shown. The color coding in panel **B** indicates matching phentypes.

REFERENCES AND NOTES

1. A. Pozzi, P. D. Yurchenco, R. V. Iozzo, The nature and biology of basement membranes. *Matrix Biol.* **57–58**, 1–11 (2017).

2. P. D. Yurchenco, Basement membranes: Cell scaffoldings and signaling platforms. *Cold Spring Harb. Perspect. Biol.* **3**, a004911 (2011).

3. R. Jayadev, Q. Chi, D. P. Keeley, E. L. Hastie, L. C. Kelley, D. R. Sherwood, α-Integrins dictate distinct modes of type IV collagen recruitment to basement membranes. *J. Cell Biol.* **218**, 3098–3116 (2019).

4. S. Li, Y. Qi, J. Liu, K. Mckee, Integrin and dystroglycan compensate each other to mediate laminin-dependent basement membrane assembly and epiblast polarization. *Matrix Biol.* **57-58**, 272–284 (2017).

5. R. Jayadev, D. R. Sherwood, Basement membranes. *Curr. Biol.* **27**, R207–R211 (2017).

6. X. Wang, R. E. Harris, L. J. Bayston, H. L. Ashe, Type IV collagens regulate BMP signalling in *Drosophila*. *Nature* **455**, 72–77 (2008).

7. D. R. Sherwood, Basement membrane remodeling guides cell migration and cell morphogenesis during development. *Curr. Opin. Cell Biol.* **72**, 19–27 (2021).

8. A. Nyström, O. Bornert, T. Kühl, Cell therapy for basement membrane-linked diseases. *Matrix Biol.* **57–58**, 124–139 (2017).

9. M. H. Foster, Basement membranes and autoimmune diseases. *Matrix Biol.* **57–58**, 149–168 (2017).

10. A. Naba, K. R. Clauser, C. A Whittaker, S. A. Carr, K. K. Tanabe, R. O. Hyne, Extracellular matrix signatures of human primary metastatic colon cancers and their metastases to liver. *BMC Cancer* **14**, 518 (2014).

11. E. C. Tsilibary, Microvascular basement membranes in diabetes mellitus. *J. Pathol.* **200**, 537–546 (2003).

12. M. Randles, F. Lausecker, Q. Kong, H. Suleiman, G. Reid, M. Kolatsi-Joannou, P. Tian, S. Falcone, B. Davenport, P. Potter, T. Van Agtmael, J. Norman, D. Long, M. Humphries, J. Miner, R. Lennon, Identification of an altered matrix signature in kidney aging and disease. *J. Am. Soc. Nephrol.* **32**, 1713–1732 (2021).

13. M. J. Randles, M. J. Humphries, R. Lennon, Proteomic definitions of basement membrane composition in health and disease. *Matrix Biol.* **57–58**, 12–28 (2017).

14. A. C. Teuscher, E. Jongsma, M. N. Davis, C. Statzer, J. M. Gebauer, A. Naba, C. Y. Ewald, The in-silico characterization of the *Caenorhabditis elegans* matrisome and proposal of a novel collagen classification. *Matrix Biol. Plus* **1**, 100001 (2019).

15. R. O. Hynes, A. Naba, Overview of the matrisome—An inventory of extracellular matrix constituents and functions. *Cold Spring Harb. Perspect. Biol.* **4**, a004903 (2012).

16. P. Nauroy, S. Hughes, A. Naba, F. Ruggiero, The in-silico zebrafish matrisome: A new tool to study extracellular matrix gene and protein functions. *Matrix Biol.* **65**, 5–13 (2018).

17. H. Suleiman, L. Zhang, R. Roth, J. E. Heuser, J. H. Miner, A. S. Shaw, A. Dani, Nanoscale protein architecture of the kidney glomerular basement membrane. *eLife* **2**, e01149 (2013).

18. M. Uhlén, L. Fagerberg, B. M. Hallström, C. Lindskog, P. Oksvold, A. Mardinoglu, Å. Sivertsson, C. Kampf, E. Sjöstedt, A. Asplund, I. M. Olsson, K. Edlund, E. Lundberg, S. Navani, C. Al-Khalili Szigyarto, J. Odeberg, D. Djureinovic, J. O. Takanen, S. Hober, T. Alm, P.-H. Edqvist, H. Berling, H. Tegel, J. Mulder, J. Rockberg, P. Nilsson, J. M. Schwenk, M. Hamsten, K. von Feilitzen, M. Forsberg, L. Persson, F. Johansson, M. Zwahlen, G. von Heijne, J. Nielsen, F. Pontén, Proteomics. Tissue-based map of the human proteome. *Science* **347**, 1260419 (2015).

19. K. Tsutsui, H. Machida, A. Nakagawa, K. Ahn, R. Morita, K. Sekiguchi, J. H. Miner, H. Fujiwara, Mapping the molecular and structural specialization of the skin basement membrane for inter-tissue interactions. *Nat. Commun.* **12**, 2577 (2021).

20. R. Manabe, K. Tsutsui, T. Yamada, M. Kimura, I. Nakano, C. Shimono, N. Sanzen, Y. Furutani, T. Fukuda, Y. Oguri, K. Shimamoto, D. Kiyozumi, Y. Sato, Y. Sado, H. Senoo, S. Yamashina, S. Fukuda, J. Kawai, N. Sugiura, K. Kimata, Y. Hayashizaki, K. Sekiguchi, Transcriptome-based systematic identification of extracellular matrix proteins. *Proc. Natl. Acad. Sci. U.S.A.* **105**, 12849–12854 (2008).

21. D. P. Keeley, E. Hastie, R. Jayadev, L. C. Kelley, Q. Chi, S. G. Payne, J. L. Jeger, B. D. Hoffman, D. R. Sherwood, Comprehensive endogenous tagging of basement membrane components reveals dynamic movement within the matrix scaffolding. *Dev. Cell* **54**, 60–74.e7 (2020).

22. M. Caulfield, J. Davies, M. Dennys, L. Elbahy, T. Fowler, S. Hill, T. Hubbard, L. Jostins, N. Maltby, J. Mahon-Pearson, G. M. Vean, K. Nevin-Ridley, M. Parker, V. Parry, A. Rendon, L. Riley, C. Turnbull, K. Woods, The national genomics research and healthcare knowledgebase. *Figshare* doi:10.6084/m9.figshare.4530893.v5 (2019).

23. The Gene Ontology Consortium, The gene ontology resource: Enriching a gold mine. *Nucleic Acids Res.* **49**, D325–D334 (2021).

24. D. Szklarczyk, A. L. Gable, D. Lyon, A. Junge, S. Wyder, J. Huerta-Cepas, M. Simonovic, N. T. Doncheva, J. H. Morris, P. Bork, L. J. Jensen, C. von Mering, STRING v11: Protein-protein association networks with increased coverage, supporting functional discovery in genome-wide experimental datasets. *Nucleic Acids Res.* **47**, D607–D613 (2019).

25. M. A. Wouters, I. Rigoutsos, C. K. Chu, L. L. Feng, D. B. Sparrow, S. L. Dunwoodie, Evolution of distinct EGF domains with specific functions. *Protein Sci.* **14**, 1091–1103 (2005).

26. M. Costell, E. Gustafsson, A. Aszódi, M. Mörgelin, W. Bloch, E. Hunziker, K. Addicks, R. Timpl, R. Fässler, Perlecan maintains the integrity of cartilage and some basement membranes. *J. Cell Biol.* **147**, 1109–1122 (1999).

27. T. M. Rogalski, B. D. Williams, G. P. Mullen, D. G. Moerman, Products of the unc-52 gene in *Caenorhabditis elegans* are homologous to the core protein of the mammalian basement membrane heparan sulfate proteoglycan. *Genes Dev.* **7**, 1471–1484 (1993).

28. J. C. Pastor-Pareja, T. Xu, Shaping cells and organs in *Drosophila* by opposing roles of fat body-secreted collagen IV and perlecan. *Dev. Cell* **21**, 245–256 (2011).

29. A. J. Isabella, S. Horne-Badovinac, Dynamic regulation of basement membrane protein levels promotes egg chamber elongation in *Drosophila*. *Dev. Biol.* **406**, 212–221 (2015).

30. R. S. Wu, I. I. Lam, H. Clay, D. N. Duong, R. C. Deo, S. R. Coughlin, A rapid method for directed gene knockout for screening in G0 zebrafish. *Dev. Cell* **46**, 112–125.e4 (2018).

31. T. J. Carney, N. M. Feitosa, C. Sonntag, K. Slanchev, J. Kluger, D. Kiyozumi, J. M. Gebauer, J. C. Talbot, C. B. Kimmel, K. Sekiguchi, R. Wagener, H. Schwarz, P. W. Ingham, M. Hammerschmidt, Genetic analysis of fin development in zebrafish identifies furin and hemicentin1 as potential novel fraser syndrome disease genes. *PLOS Genet.* **6**, e1000907 (2010).

32. J. H. Suh, J. H. Miner, The glomerular basement membrane as a barrier to albumin. *Nat. Rev. Nephrol.* **9**, 470–477 (2013).

33. R. W. Naylor, E. Lemarie, A. J.-Crawford, J. Bernard Davenport, A. Mironov, M. Lowe, R. Lennon, A novel nanoluciferase transgenic reporter to measure proteinuria in zebrafish. bioRxiv 10.1101/2021.07.19.452884 [**Preprint**]. 2021.

34. J. B. Skeath, B. A. Wilson, S. E. Romero, M. J. Snee, Y. Zhu, H. Lacin, The extracellular metalloprotease AdamTS-A anchors neural lineages in place within and preserves the architecture of the central nervous system. *Development* **144**, 3102–3113 (2017).

35. S. Köhler, M. Gargano, N. Matentzoglu, L. C. Carmody, D. L.-Smith, N. A. Vasilevsky, D. Danis, G. Balagura, G. Baynam, A. M. Brower, T. J. Callahan, C. G. Chute, J. L. Est, P. D. Galer, S. Ganesan, M. Griese, M. Haimel, J. Pazmandi, M. Hanauer, N. L. Harris, M. J. Hartnett, M. Hastreiter, F. Hauck, Y. He, T. Jeske, H. Kearney, G. Kindle, C. Klein, K. Knoflach, R. Krause, D. Lagorce, J. A. Mc Murry, J. A. Miller, M. C. M.-Torres, R. L. Peters, C. K. Rapp, A. M. Rath, S. A. Rind, A. Z. Rosenberg, M. M. Segal, M. G. Seidel, D. Smedley, T. Talmy, Y. Thomas, S. A. Wiafe, J. Xian, Z. Yüksel, I. Helbig, C. J. Mungall, M. A. Haendel, P. N. Robinson, The human phenotype ontology in 2021. *Nucleic Acids Res.* **49**, D1207–D1217 (2021).

36. A. R. Martin, E. Williams, R. E. Foulger, S. Leigh, L. C. Daugherty, O. Niblock, I. U. S. Leong, K. R. Smith, O. Gerasimenko, E. Haraldsdottir, E. Thomas, R. H. Scott, E. Baple, A. Tucci, H. Brittain, A. de Burca, K. Ibañez, D. Kasperaviciute, D. Smedley, M. Caulfield, A. Rendon, E. M. McDonagh, PanelApp crowdsources expert knowledge to establish consensus diagnostic gene panels. *Nat. Genet.* **51**, 1560–1565 (2019).

37. J. S. Amberger, C. A. Bocchini, F. Schiettecatte, A. F. Scott, A. Hamosh, OMIM.org: Online Mendelian Inheritance in Man (OMIM), an online catalog of human genes and genetic disorders. *Nucleic Acids Res.* **43**, D789–D798 (2015).

38. K. J. Karczewski, L. C. Francioli, G. Tiao, B. B. Cummings, J. Alföldi, Q. Wang, R. L. Collins, K. M. Laricchia, A. Ganna, D. P. Birnbaum, L. D. Gauthier, H. Brand, M. Solomonson, N. A. Watts, D. Rhodes, M. S.-Berk, E. M. England, E. G. Seaby, J. A. Kosmicki, R. K. Walters, K. Tashman, Y. Farjoun, E. Banks, T. Poterba, A. Wang, C. Seed, N. Whiffin, J. X. Chong, K. E. Samocha, E. Pierce-Hoffman, Z. Zappala, A. H. O'Donnell-Luria, E. V. Minikel, B. Weisburd, M. Lek, J. S. Ware, C. Vittal, I. M. Armean, L. Bergelson, K. Cibulskis, K. M. Connolly, M. Covarrubias, S. Donnelly, S. Ferriera, S. Gabriel, J. Gentry, N. Gupta, T. Jeandet, D. Kaplan, C. Llanwarne, R. Munshi, S. Novod, N. Petrillo, D. Roazen, V. Ruano-Rubio, A. Saltzman, M. Schleicher, J. Soto, K. Tibbetts, C. Tolonen, G. Wade, M. E. Talkowski; Genome Aggregation Database Consortium, B. M. Neale, M. J. Daly, D. G. MacArthur, The mutational constraint spectrum quantified from variation in 141,456 humans. *Nature* **581**, 434–443 (2020).

39. A. Byron, M. J. Randles, J. D. Humphries, A. Mironov, H. Hamidi, S. Harris, P. W. Mathieson, M. A. Saleem, S. C. Satchell, R. Zent, M. J. Humphries, R. Lennon, Glomerular cell cross-talk influences composition and assembly of extracellular matrix. *J. Am. Soc. Nephrol.* **25**, 953–966 (2014).

40. H. B. Thomas, K. A. Wood, W. A. Buczek, C. T. Gordon, V. Pingault, T. Attié-Bitach, K. E. Hentges, V. C. Varghese, J. Amiel, W. G. Newman, R. T. O'Keefe, EFTUD2 missense variants disrupt protein function and splicing in mandibulofacial dysostosis Guion-Almeida type. *Hum. Mutat.* **41**, 1372–1382 (2020).

41. G. Ramena, Y. Yin, Y. Yu, V. Walia, R. C. Elble, CLCA2 interactor EVA1 is required for mammary epithelial cell differentiation. *PLOS ONE* **11**, e0147489 (2016).

42. M. Wesdorp, S. M.-Cuesta, T. Peters, A.M. Celaya, A. Oonk, M. Schraders, J. Oostrik, E. G.-Rosas, A.J. Beynon, B.P. Hartel, K. Okkersen, H.J.P.M. Koenen, J. Weeda, S. Lelieveld, N.C. Voermans, I. Joosten, C.B. Hoyng, P. Lichtner, H.P.M. Kunst, I. Feenstra, S.E. de Bruijn; DOOFNL Consortium, R.J.C. Admiraal, H.G. Yntema, E. van Wijk, I. del Castillo, P. Serra, I. Varela-Nieto, R.J.E. Pennings, H. Kremer, MPZL2, Encoding the epithelial junctional protein myelin protein zero-like 2, is essential for hearing in man and mouse. *Am. J. Hum. Genet.* **103**, 74–88 (2018).

43. H. Colognato, P. D. Yurchenco, Form and function: The laminin family of heterotrimers. *Dev. Dyn.* **218**, 213–234 (2000).

44. L. Ritié, C. Spenlé, J. Lacroute, A.-Laure B.-Bellemin, O. Lefebvre, C. B.-Feysot, B. Jost, A. Klein, C. Arnold, M. Kedinger, D. Bagnard, G. Orend, P. S.-Assmann, Abnormal Wnt and PI3Kinase signaling in the malformed intestine of lama5 deficient mice. *PLOS ONE* **7**, e37710 (2012).

45. L. K. Jones, R. Lam, K. K. Mc Kee, M. Aleksandrova, J. Dowling, S. I. Alexander, A. Mallawaarachchi, D. L. Cottle, K. M. Short, L. Pais, J. H. Miner, A. J. Mallett, C. Simons, H. M. Carthy, P. D. Yurchenco, I. M. Smyth, A mutation affecting laminin alpha 5

polymerisation gives rise to a syndromic developmental disorder. *Development* **147**, dev189183 (2020).

46. S. Sampaolo, F. Napolitano, A. Tirozzi, M. G. Reccia, L. Lombardi, O. Farina, A. Barra, F. Cirillo, M. A. B. Melone, F. Gianfrancesco, G. D. Iorio, T. Esposito, Identification of the first dominant mutation of LAMA5 gene causing a complex multisystem syndrome due to dysfunction of the extracellular matrix. *J. Med. Genet.* **54**, 710–720 (2017).

47. Y. Wu, G. Ge, Complexity of type IV collagens: From network assembly to function. *Biol. Chem.* **400**, 565–574 (2019).

48. A. Woods, J. R. Couchman, Proteoglycan isolation and analysis. *Curr. Protoc. Cell Biol.* **80**, e59 (2018).

49. A. G. Campbell, L. I. Fessler, T. Salo, J. H. Fessler, Papilin: A *Drosophila* proteoglycan-like sulfated glycoprotein from basement membranes. *J. Biol. Chem.* **262**, 17605–17612 (1987).

50. P. D. Yurchenco, B. L. Patton, Developmental and pathogenic mechanisms of basement membrane assembly. *Curr. Pharm. Des.* **15**, 1277–1294 (2009).

51. Y. Matsubayashi, A. Louani, A. Dragu, B. J. Sánchez-Sánchez, E. Serna-Morales, L. Yolland, A. Gyoergy, G. Vizcay, R. A. Fleck, J. M. Heddleston, T.-L. Chew, D. E. Siekhaus, B. M. Stramer, A moving source of matrix components is essential for de novo basement membrane formation. *Curr. Biol.* **27**, 3526–3534.e4 (2017).

52. M. A. Morrissey, D. R. Sherwood, An active role for basement membrane assembly and modification in tissue sculpting. *J. Cell Sci.* **128**, 1661–1668 (2015).

53. Y. Kubota, K. Nagata, A. Sugimoto, K. Nishiwaki, Tissue architecture in the *Caenorhabditis elegans* gonad depends on interactions among fibulin-1, type IV collagen and the ADAMTS extracellular protease. *Genetics* **190**, 1379–1388 (2012).

54. P. Brouillard, L. Dupont, R. Helaers, R. Coulie, G. E. Tiller, J. Peeden, A. Colige, M. Vikkula, Loss of ADAMTS3 activity causes Hennekam lymphangiectasia-lymphedema syndrome 3. *Hum. Mol. Genet.* **26**, 4095–4104 (2017).

55. S. Bunt, C. Hooley, N. Hu, C. Scahill, H. Weavers, H. Skaer, Hemocyte-secreted type IV collagen enhances BMP signaling to guide renal tubule morphogenesis in *Drosophila*. *Dev. Cell* **19**, 296–306 (2010).

56. J. Crest, A. Diz-Muñoz, D.-Y. Chen, D. A. Fletcher, D. Bilder, Organ sculpting by patterned extracellular matrix stiffness. *eLife* **6**, e24958 (2017).

57. J. S. Harunaga, A. D. Doyle, K. M. Yamada, Local and global dynamics of the basement membrane during branching morphogenesis require protease activity and actomyosin contractility. *Dev. Biol.* **394**, 197–205 (2014).

58. M. S. Thomsen, L. J. Routhe, T. Moos, The vascular basement membrane in the healthy and pathological brain. *J. Cereb. Blood Flow Metab.* **37**, 3300–3317 (2017).

59. J. H. Miner, Type IV collagen and diabetic kidney disease. *Nat. Rev. Nephrol.* **16**, 3–4 (2020).

60. O. Uspenskaia, M. Liebetrau, J. Herms, A. Danek, G. F. Hamann, Aging is associated with increased collagen type IV accumulation in the basal lamina of human cerebral microvessels. *BMC Neurosci.* **5**, 37 (2004).

61. T. Xiao, W. Staub, E. Robles, N. J. Gosse, G. J. Cole, H. Baier, Assembly of lamina-specific neuronal connections by slit bound to type IV collagen. *Cell* **146**, 164–176 (2011).

62. D. Piecha, C. Wiberg, M. Mörgelin, D. P. Reinhardt, F. Deák, P. Maurer, M. Paulsson, Matrilin-2 interacts with itself and with other extracellular matrix proteins. *Biochem. J.* **367**, 715–721 (2002).

63. D. F. Barker, S. L. Hostikka, J. Zhou, L. T. Chow, A. R. Oliphant, S. C. Gerken, M. C. Gregory, M. H. Skolnick, C. L. Atkin, K. Tryggvason, Identification of mutations in the COL4A5 collagen gene in Alport syndrome. *Science* **248**, 1224–1227 (1990).

64. J. H. Miner, J. Cunningham, J. R. Sanes, Roles for laminin in embryogenesis: Exencephaly, syndactyly, and placentopathy in mice lacking the laminin alpha5 chain. *J. Cell Biol.* **143**, 1713–1723 (1998).

65. I. Papatheodorou, P. Moreno, J. Manning, A. M.-Pomer Fuentes, N. George, S. Fexova, N. A. Fonseca, A. Füllgrabe, M. Green, N. Huang, L. Huerta, H. Iqbal, M. Jianu, S. Mohammed, L. Zhao, A. F. Jarnuczak, S. Jupp, J. Marioni, K. Meyer, R. Petryszak, C. A. Prada Medina, C. T.-López, S. Teichmann, J. A. Vizcaino, A. Brazma, Expression atlas update: From tissues to single cells. *Nucleic Acids Res.* **48**, D77–D83 (2020).

66. P. Shannon, A. Markiel, O. Ozier, N. S. Baliga, J. T. Wang, D. Ramage, N. Amin, B. Schwikowski, T. Ideker, Cytoscape: A software environment for integrated models of biomolecular interaction networks. *Genome Res.* **13**, 2498–2504 (2003).

67. Y. Assenov, F. Ramírez, S.-E. Schelhorn, T. Lengauer, M. Albrecht, Computing topological parameters of biological networks. *Bioinformatics* **24**, 282–284 (2008).

68. T. Stiernagle, Maintenance of C. elegans. *WormBook* **11**, 1–11 (2006).

69. C. B. Kimmel, W. W. Ballard, S. R. Kimmel, B. Ullmann, T. F. Schilling, Stages of embryonic development of the zebrafish. *Dev. Dyn.* **203**, 253–310 (1995).

70. J.-F. Rual, J. Ceron, J. Koreth, T. Hao, A.-S. Nicot, T. Hirozane-Kishikawa, J. Vandenhaute, S. H. Orkin, D. E. Hill, S. van den Heuvel, M. Vidal, Toward improving *Caenorhabditis elegans* phenome mapping with an ORFeome-based RNAi library. *Genome Res.* **14**, 2162–2168 (2004).

71. R. S. Kamath, A. G. Fraser, Y. Dong, G. Poulin, R. Durbin, M. Gotta, A. Kanapin, N. le Bot, S. Moreno, M. Sohrmann, D. P. Welchman, P. Zipperlen, J. Ahringer, Systematic functional analysis of the *Caenorhabditis elegans* genome using RNAi. *Nature* **421**, 231–237 (2003).

72. R. S. Kamath, A. G. Fraser, Y. Dong, G. Poulin, R. Durbin, M. Gotta, A. Kanapin, N. L. Bot, S. Moreno, M. Sohrmann, D. P. Welchman, P. Zipperlen, J. Ahringer, Kidney organoids recapitulate human basement membrane assembly in health and disease. *eLife* **11**, e73486 (2022).

73. A. Edelstein, N. Amodaj, K. Hoover, R. Vale, N. Stuurman, Computer control of microscopes using µManager. *Curr. Protoc. Mol. Biol.* **14**, Unit14.20 (2010).

74. J. Schindelin, I. A.-Carreras, E. Frise, V. Kaynig, M. Longair, T. Pietzsch, S. Preibisch, C. Rueden, S. Saalfeld, B. Schmid, Fiji: An open-source platform for biological-image analysis. *Nat. Methods* **9**, 676–682 (2012).

75. M. J. Randles, S. Collinson, T. Starborg, A. Mironov, M. Krendel, E. Königshausen, L. Sellin, I. S. D. Roberts, K. E. Kadler, J. H. Miner, R. Lennon, Three-dimensional electron microscopy reveals the evolution of glomerular barrier injury. *Sci. Rep.* **6**, 35068 (2016).

76. E. Wershof, D. Park, D. J. Barry, R. P. Jenkins, A. Rullan, A. Wilkins, K. Schlegelmilch, I. Roxanis, K. I. Anderson, P. A. Bates, E. Sahai, A FIJI macro for quantifying pattern in extracellular matrix. *Life Sci. Alliance.* **4**, e202000880 (2021).

77. M. H. Guo, L. Plummer, Y.-M. Chan, J. N. Hirschhorn, M. F. Lippincott, Burden testing of rare variants identified through exome sequencing via publicly available control data. *Am. J. Hum. Genet.* **103**, 522–534 (2018).

78. D. Smedley, J. O. B. Jacobsen, M. Jäger, S. Köhler, M. Holtgrewe, M. Schubach, E. Siragusa, T. Zemojtel, O. J. Buske, N. L. Washington, W. P. Bone, M. A. Haendel, P. N. Robinson, Next-generation diagnostics and disease-gene discovery with the Exomiser. *Nat. Protoc.* **10**, 2004–2015 (2015).

79. L. A. Kelley, S. Mezulis, C. M. Yates, M. N. Wass, M. J. E. Sternberg, The Phyre2 web portal for protein modeling, prediction and analysis. *Nat. Protoc.* **10**, 845–858 (2015).

80. E. A. Malone, J. H. Thomas, A screen for nonconditional dauer-constitutive mutations in Caenorhabditis elegans. *Genetics* **136**, 879–886 (1994).
